# Supplementary material for: Defining bottlenecks and opportunities for Lassa virus neutralization by structural profiling of vaccine-induced polyclonal antibody responses
Source: Cell Rep. 2024 Sep 6;43(9):114708. doi: 10.1016/j.celrep.2024.114708 (PMC11422484; doi:10.1016/j.celrep.2024.114708)
Supplement: Document S1. Figures S1–S7 and Tables S1–S3 [file mmc1.pdf]

Cell Reports, Volume *43*

Supplemental information

Defining bottlenecks and opportunities for Lassa virus neutralization by structural profiling of vaccine-induced polyclonal antibody responses

Philip J.M. Brouwer, Hailee R. Perrett, Tim Beaumont, Haye Nijhuis, Sabine Kruijer, Judith A. Burger, Ilja Bontjer, Wen-Hsin Lee, James A. Ferguson, Martin Schauflinger, Helena Müller-Kräuter, Rogier W. Sanders, Thomas Strecker, Marit J. van Gils, and Andrew B. Ward

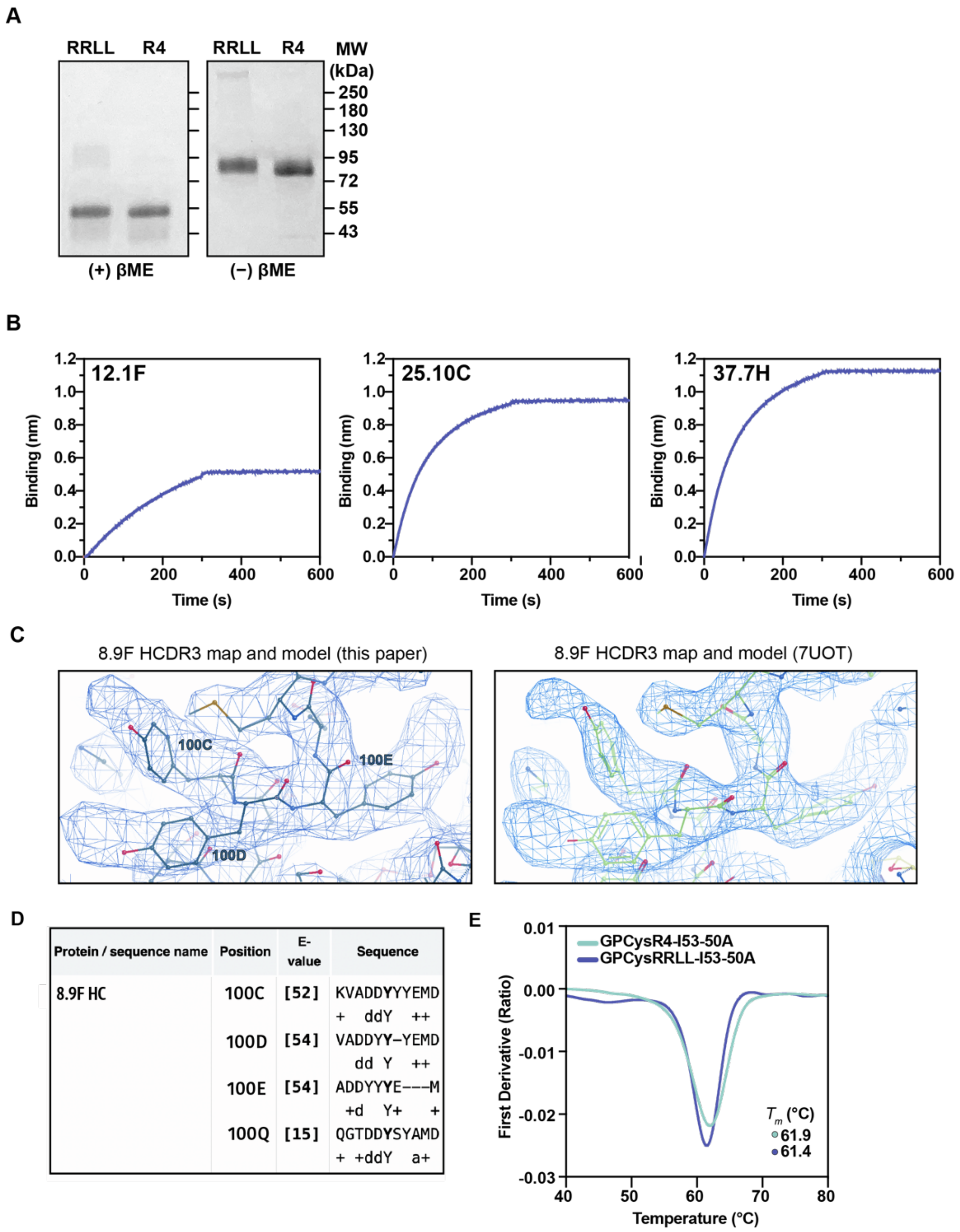

Figure S1. Characterization and comparison of GPCysRRLL-I53-50A and its interaction with 8.9F, related to Figure 1. (A) SDS-PAGE comparison of cleavage between GPCysRRLL-I53-50A co-transfected with S1P and GPCysR4-I53-50A co-transfected with furin in reducing (left) and non-reducing (right) conditions. Coomassie staining was used for protein visualization. **(B)** BLI sensorgrams indicating immobilized 12.1F, 25.10C, and 37.7H IgGs binding to GPCysRRLL-I53-50A at a concentration of 120 nM. **(C)** Close up of the model and maps described here (left) and 7UOT[1] (right), focusing on the HCDR3 of 8.9F around residues 100C, 100D, and 100E. **(D)** Result from Expasy's Sulfinator**.**[2] Presence of sulfated tyrosines is predict based on four different Hidden Markov models (HMM).[2] The position of predicted sulfated tyrosines are depicted in the second column. Lower case letters in the fourth column indicate direct matches with the HMM

chain and pluses mark positions with a positive score. **(E)** Thermostability of GPCysRRLL-I53-50A and GPCysR4-I53-50A assessed by nanoDSF. Melting temperatures (T_m) are calculated as the inflection point (circles) of the ratio of signal at 350 and 330 nM. Each melting curve is a representative of triplicate curves with T_m within ±0.1°C.

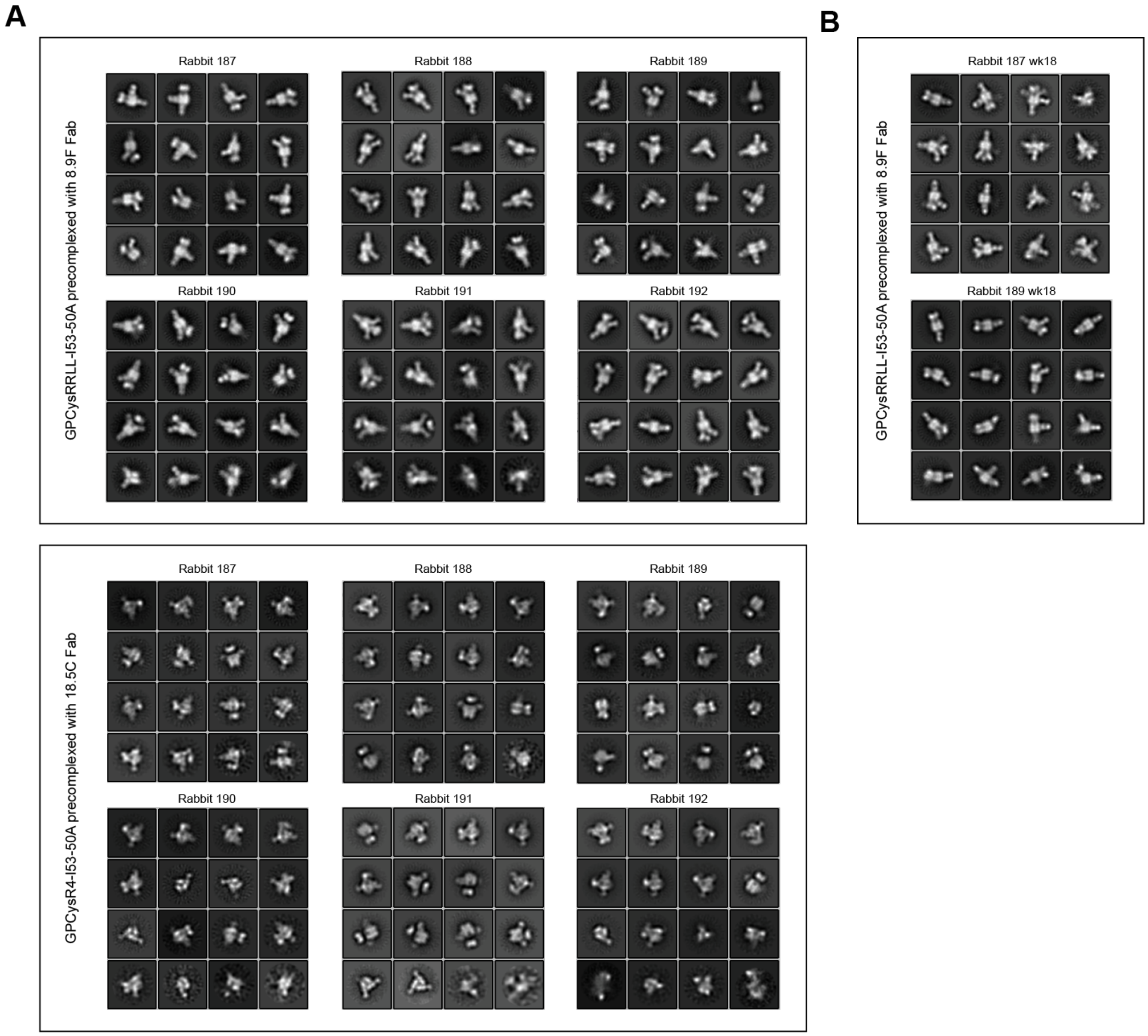

Figure S2. 2D classes of selected particles for the generation of 3D maps, related to Figure 2. (A) 2D class averages of nsEMPEM experiments with week 30 pAbs from rabbit 187-192 using GPCysRRLL-I53-50A pre-complexed with 8.9F (top) and GPCysR4-I53-50A pre-complexed with 18.5C (bottom). Classes shown are the selected classes that were selected for 3D classification, reclassified to 16 classes. **(B)** 2D class averages of nsEMPEM experiments with week 18 pAbs from rabbit 187 and 189 using GPCysRRLL-I53-50A pre-complexed with 8.9F. Classes shown are the selected classes that were selected for 3D classification, reclassified to 16 classes.

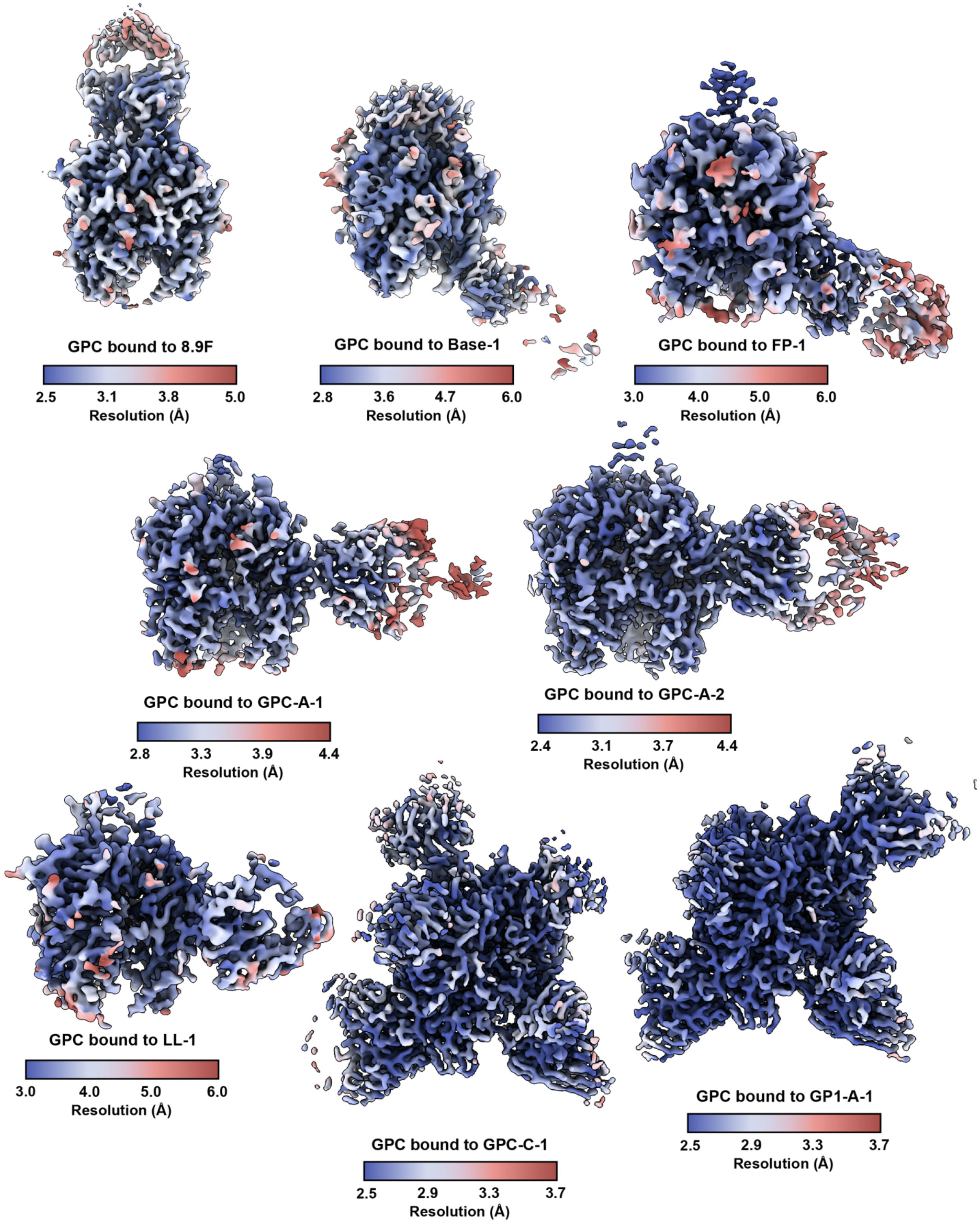

Figure S3. Local resolution for EM maps of LASV GPC and 8.9F, Base-1, FP-1, GPC-A-1, GPC-A-2, LL-1, GPC-C-1, and GP1-A-1, related to Figures 3, 4, and 6. Local resolutions calculated using cryoSPARC v4.5.3[3] using an FSC threshold of 0.143. Maps visualized in ChimeraX.[4]

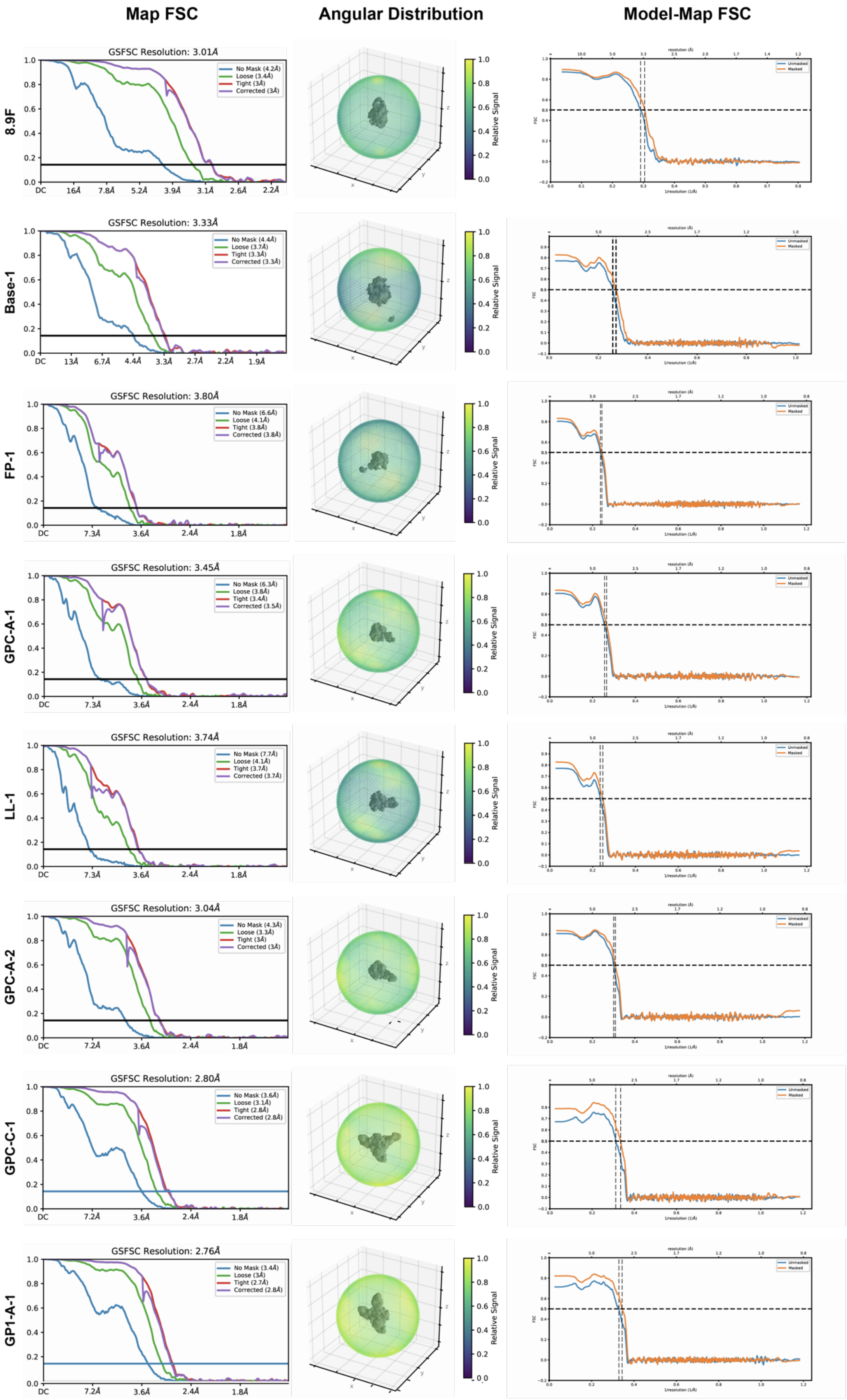

Map FSC
Angular Distribution
Model-Map FSC
8.9F
GSFSC Resolution: 3.01Å
Base-1
GSFSC Resolution: 3.33Å
FP-1
GSFSC Resolution: 3.80Å
GPC-A-1
GSFSC Resolution: 3.45Å
LL-1
GSFSC Resolution: 3.74Å
GPC-A-2
GSFSC Resolution: 3.04Å
GPC-C-1
GSFSC Resolution: 2.80Å
GP1-A-1
GSFSC Resolution: 2.76Å
Relative Signal

Figure S4. FSC plots for cryo-EM maps, angular distribution plots and model-map FSC plots for the maps that were used model building, related to Figures 3, 4, and 6. Reported resolutions of maps coincide with an FSC cutoff of 0.143. FSCs are presented from local refinement jobs except for GPC-C-1 and GP1-A-1 which come from a global refinement. FSC plots for maps (left) and angular distribution plots (center) were generated using cryoSPARC v4.5.3.[3] The model-map FCS plots (right) were generated using Phenix 1.21.[5]

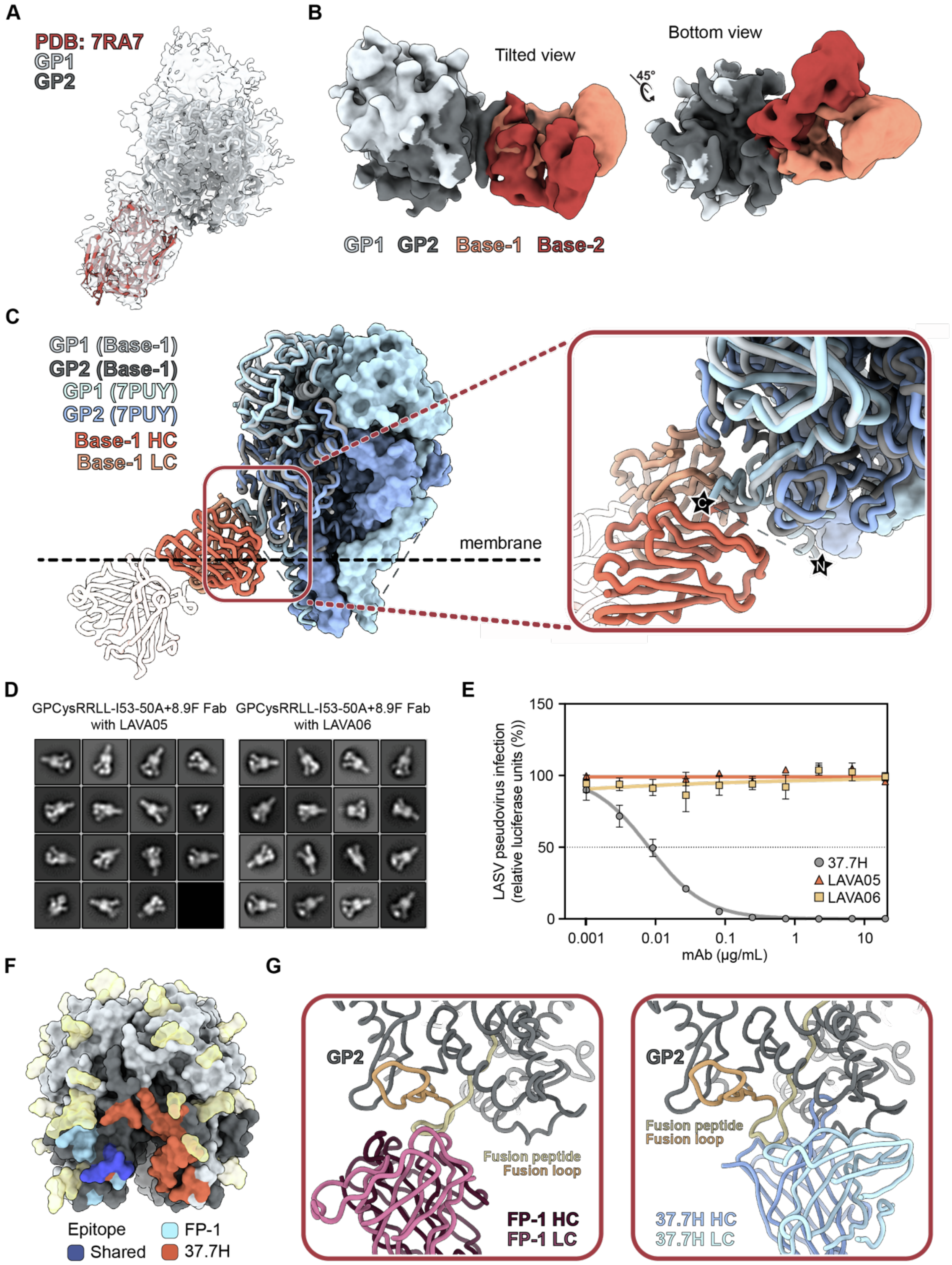

Figure S5. Structural differences between Base-1 and Base-2 responses, their inability to bind membrane-embedded GPC, neutralization of LAVA05 and LAVA06, and structural characterization of the FP-1 response, related to Figure 3. (A) Cryo-EM map of Base-2 with a rabbit Fab (PDB: 7RA7) docked in the pFab density. **(B)** Overlaid Gaussian-filtered maps of base antibodies demonstrating the distinct angles of approach taken by Base-1 (orange) and

Base-2 (red) to engage the GPC. **(C)** Overlay of full-length GPC (PDB: 7PUY[6]) with the Base-1 model revealing accessibility issues for Base-1 binding by the membrane and/or SSP. The constant region of the Fab (transparent) was generated by docking a model of a full rabbit Fab (PDB: 7RA7) into the density and removing the variable domain. The inset shows the unmodelled part of the SSP (displayed as pseudobond), referred to by Katz et al.[6] as "hydrophobic 2" (residues 36-58), with its N terminus (right) and C terminus (left) depicted as stars.[6] It is probable that the 22 amino acid "hydrophobic 2" structure resides in this area, sterically blocking binding of base-targeting pAb responses. **(D)** 2D class averages of nsEMPEM experiments with LAVA05 and LAVA05 using GPCysRRLL-I53-50A pre-complexed with 8.9F. Classes shown are the selected classes that were selected for 3D classification, reclassified to 16 classes. **(E)** Pseudovirus neutralization of LAVA05 and LAVA06. The mAb 37.7H was used as a positive control. Shown are the mean and SD of three technical replicates. **(F)** Comparison of the epitope footprint of FP-1 with prototypical GPC-B mAb 37.7H. To determine the footprint of 37.7H, all GPC residues within a radius of 4 Å from 37.7H were highlighted. For the footprint of FP-1, all GPC residues within a radius of 6 Å from FP-1 were highlighted to compensate for the absence of modeled sidechains past Cβ. **(G)** Close up of GP2 engagement by FP-1 (left) and 37.7H (right), illustrating the difference in fusion loop/fusion peptide engagement.

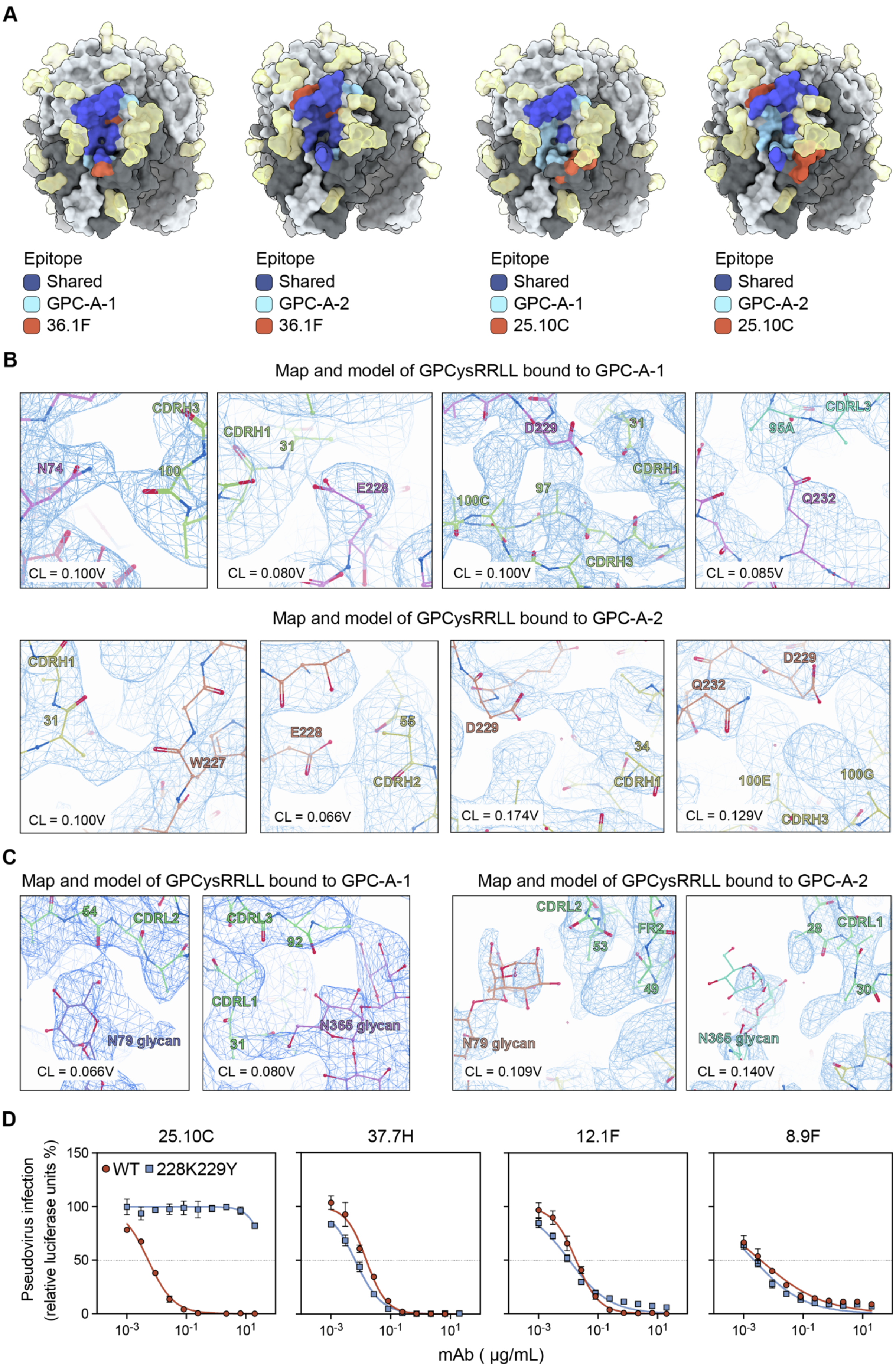

A
Epitope
Shared
GPC-A-1
36.1F
Epitope
Shared
GPC-A-2
36.1F
Epitope
Shared
GPC-A-1
25.10C
Epitope
Shared
GPC-A-2
25.10C
B
Map and model of GPCysRRLL bound to GPC-A-1
CDRH3
N74
100
CL = 0.100V
CDRH1
31
E228
CL = 0.080V
D229
31
CDRH1
100C
97
CDRH3
CL = 0.100V
CDRL3
95A
Q232
CL = 0.085V
Map and model of GPCysRRLL bound to GPC-A-2
CDRH1
31
W227
CL = 0.100V
E228
55
CDRH2
CL = 0.066V
D229
34
CDRH1
CL = 0.174V
D229
Q232
100E
100G
CDRH3
CL = 0.129V
C
Map and model of GPCysRRLL bound to GPC-A-1
54
CDRL2
N79 glycan
CL = 0.066V
CDRL3
92
CDRL1
31
N365 glycan
CL = 0.080V
Map and model of GPCysRRLL bound to GPC-A-2
CDRL2
FR2
53
49
N79 glycan
CL = 0.109V
CDRL1
28
30
N365 glycan
CL = 0.140V
D
25.10C
37.7H
12.1F
8.9F
WT
228K229Y
Pseudovirus infection
(relative luciferase units %)
150
100
50
0
10^-3
10^-1
10^1
mAb (μg/mL)

Figure S6. Characterization of the GPC-A-1 and GPC-A-2 response and effect of the 228K229Y mutation on the neutralization potency of representative NAbs from the various competition clusters, related to Figure 4. (A) Epitope footprints of GPC-A-1 and GPC-A-2 compared to NAbs 36.1F and 25.10C.[7] . To determine the footprint of 36.1F and 25.10C, all GPC residues within a radius of 4 Å from the mAb were highlighted. For the footprint of GPC-A-1 and GPC-A-2, all GPC residues within a radius of 6 Å from the pAb were highlighted to compensate for the absence of modeled sidechains past Cβ. **(B)** Close up of map and model where the density suggests contacts for GPC-A-1 (top) and GPC-A-2 (bottom) which are also shared contact residues for 36.1F and 25.10C on GPC. Contour level (CL) is indicated in each panel. **(C)** Close up of map and model where the density demonstrates interactions between glycans on GPC and the pAb GPC-A-1 (left) and GPC-A-2 (right). Contour level (CL) is indicated in each panel**. (D)** Neutralization curves for mAbs 25.10C (GPC-A competition cluster), 37.7H (GPC-B competition cluster), 12.1F (GP1-A competition cluster), and 8.9F (GPC-C competition cluster), against Josiah pseudovirus (WT) or Josiah pseudovirus with the 228K229Y mutation. Shown are the mean and SD of three technical replicates.

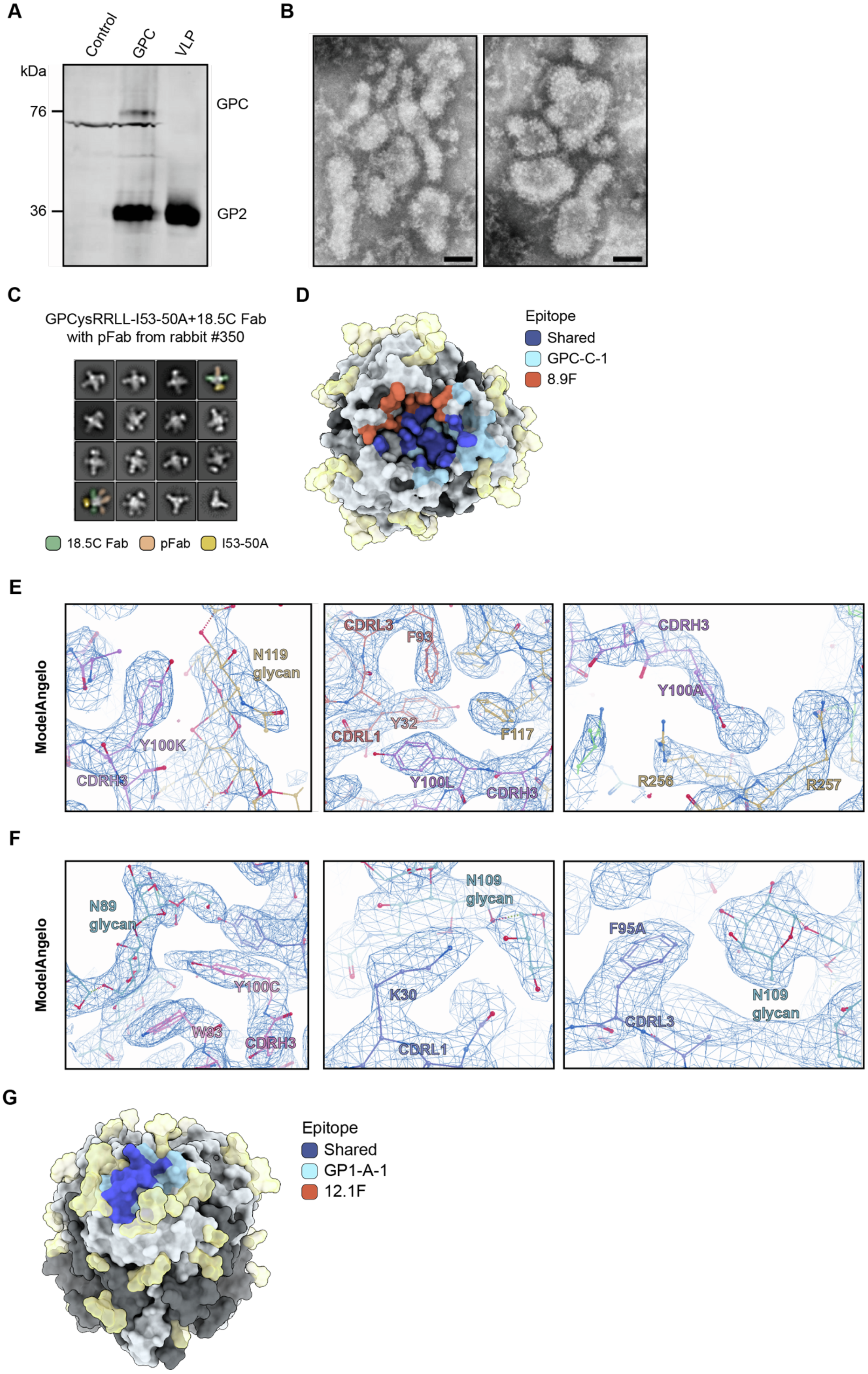
A
Control
GPC
VLP
kDa
76
36
GPC
GP2
B
C
GPCysRRLL-I53-50A+18.5C Fab
with pFab from rabbit #350
18.5C Fab
pFab
I53-50A
D
Epitope
Shared
GPC-C-1
8.9F
E
ModelAngelo
N119
glycan
Y100K
CDRH3
CDRL3
F93
Y32
CDRL1
F117
Y100L
CDRH3
CDRH3
Y100A
R256
R257
F
ModelAngelo
N89
glycan
Y100C
W93
CDRH3
N109
glycan
K30
CDRL1
F95A
N109
glycan
CDRL3
G
Epitope
Shared
GP1-A-1
12.1F

Figure S7. Characterization of a GPC-derived VLP and the humoral immune responses it elicited in rabbit 350, related to Figure 6. (A) Western blot analysis of GPC-derived VLPs purified from the cell culture supernatant of MDCK II cells stably expressing GPC. GPC/GP2 were stained with a rabbit anti-LASV GP2 antibody (α4) and an anti-rabbit antibody conjugated with Alexa Fluor 680 and visualized using the Odyssey Infrared Imaging System (LI-COR Biosciences). As a control untransfected cells were used (Control; lane 1), as well as cell lysates stably expressing GPC (GPC; lane 2). **(B)** Transmission electron microscopy was performed with purified GPC VLPs after fixation with 1% PFA for 1 h. VLP suspension was applied to formvar-coated nickel grids and incubated for 10 min. After brief washing in water, the samples were negatively stained with 1% phosphotungstic acid. Micrographs were acquired at 120 kV using a JEOL JEM1400 equipped with a TVIPS TemCam F416 camera. Scale bar indicates 50 nm. **(C)** 2D class averages of nsEMPEM experiments with pAbs from rabbit 350 using GPCysRRLL-I53-50A pre-complexed with 18.5C. Classes shown are the selected classes from two iterations of 2D classification that have been reclassified to 16 classes. Two classes are pseudo-colored according to the legend below to indicate the stabilizing Fab (18.5C Fab), pFab and I53-50A. **(D)** Epitope footprints of GPC-C-1 and NAb 8.9F.[1] To determine the footprint of 8.9F, all GPC residues within a radius of 4 Å from 8.9F were highlighted. For the footprint of GPC-C-1, all GPC residues within a radius of 6 Å from GPC-C-1 were highlighted to compensate for the absence of modeled sidechains past Cβ. **(E)** Close up of map and model highlighting presumed contacts between the CDRH3 of GPC-C-1 and GPC. The highest probability residue as determined by ModelAngelo was modeled in the poly-alanine backbone and indicated with a label. **(F)** Same as (E) but now for GP1-A-1. **(G)** Epitope footprints of GP1-A-1 and NAb 12.1F.[1] To determine the footprint of 12.1F, all GPC residues within a radius of 4 Å from 12.1F were highlighted. For the footprint of GP1-A-1, all GPC residues within a radius of 6 Å from GP1-A-1 were highlighted to compensate for the absence of modeled sidechains past Cβ.

Table S1. 8.9F antibody interactions with GP1, related to Figure 1. Amino acid interactions at the 8.9F epitope-paratope region were determined using the online-based Epitope-Analyzer platform[8]. Since Epitope-Analyzer does not consider heteroatoms, interactions between sulfated tyrosines (TYS) and GPC were inferred from the structure using an overall distance cutoff of <4.0 Å and a distance cutoff of <3.5 Å for hydrogen bond interactions in particular.

Residue # (GPC)	Chain	Amino acid	Atom	Residue # (8.9F)	Chain	Amino acid	Distance (Å)	Predicted interaction
117	A	PHE	CE1-OH	100E	H	TYS	3.7	Van-der-Waals
119	A	ASN	OD1-CE2	100E	H	TYS	3.6	Van-der-Waals
121	A	SER	CB-CB	100D	H	TYS	3.7	Van-der-Waals
125	A	LYS	NZ-OD1	100A	H	ASP	2.4	Salt-Bridge
125	A	LYS	NZ-OD2	100A	H	ASP	3.3	Salt-Bridge
150	A	TYR	CE2-O	100H	H	ASP	3.9	Van-der-Waals
150	A	TYR	CE1-CG2	100I	H	VAL	3.9	Hydr-Phbc
256	A	ARG	NH1-OE1	100L	H	GLN	3.1	Hydro-Bond
256	A	ARG	NH1-CE	100G	H	MET	3.2	Van-der-Waals
150	B	TYR	OH-OD2	100O	H	ASP	3.4	Hydro-Bond
256	B	ARG	NE-OH	100C	H	TYS	3.5	Hydro-Bond
256	B	ARG	NH2-O1	100C	H	TYS	3.1	Salt-Bridge
256	B	ARG	NE-O1	100C	H	TYS	3.6	Salt-Bridge
256	B	ARG	CD-CE2	100D	H	TYS	3.9	Van-der-Waals
257	B	ARG	N-O1	100D	H	TYS	2.6	Hydro-Bond
257	B	ARG	NE-O1	100D	H	TYS	3.8	Salt-Bridge
257	B	ARG	NE-O2	100D	H	TYS	3.0	Salt-Bridge
257	B	ARG	NH2-O2	100D	H	TYS	2.9	Salt-Bridge
258	B	LEU	CB-CE2	100D	H	TYS	3.9	Hydr-Phbc
258	B	LEU	CB-CZ	100D	H	TYS	3.9	Hydr-Phbc
258	B	LEU	N-OH	100D	H	TYS	3.5	Hydro-Bond
150	C	TYR	OH-OD1	100A	H	ASP	2.4	Hydro-Bond
150	C	TYR	CE1-CB	100	H	ALA	3.5	Hydr-Phbc
150	C	TYR	CZ-CB	100	H	ALA	3.9	Hydr-Phbc
256	C	ARG	CG-OD1	100H	H	ASP	3.2	Van-der-Waals
256	C	ARG	CD-CB	100G	H	MET	3.3	Van-der-Waals
257	C	ARG	N-OD2	100H	H	ASP	2.6	Hydro-Bond
114	C	ASN	O-CA	30A	L	GLY	3.23	Van-der-Waals
114	C	ASN	ND2-CB	30	L	ILE	3.44	Van-der-Waals
115	C	HIS	CB-OD1	31	L	ASN	3.72	Van-der-Waals
116	C	LYS	N-CB	93	L	SER	3.95	Van-der-Waals
117	C	PHE	CZ-CE2	91	L	TYR	3.43	Hydr-Phbc
117	C	PHE	CZ-CD2	91	L	TYR	3.46	Hydr-Phbc
117	C	PHE	CE1-CE2	91	L	TYR	3.58	Hydr-Phbc
117	C	PHE	CE2-ND2	31	L	ASN	3.67	Van-der-Waals
117	C	PHE	CE2-CB	93	L	SER	3.72	Van-der-Waals
117	C	PHE	CZ-CE2	32	L	PHE	3.84	Hydr-Phbc
117	C	PHE	CE1-CE2	32	L	PHE	3.93	Hydr-Phbc
117	C	PHE	CE1-CD2	91	L	TYR	3.97	Hydr-Phbc

Table S2: Electron Microscopy Data Bank deposition information, related to Figures 2 and 3. Maps are accessible at emdataresource.org using the listed codes. Additional maps can be found on the "Download" tab of each entry.

	EMDB ID	Time point	Specificity	# particles (composite)	Map	Microscope	Pixel size
**Rabbit 187**	EMD-43174	30	Base	7,212	additional map	Tecnai Spirit	2.06
	EMD-43174	30	FP	10,038	additional map	Tecnai Spirit	2.06
	EMD-43174	30	GPC-A	16,689	main map	Tecnai Spirit	2.06
	EMD-43175	18	Base	8,882	additional map	Tecnai Spirit	2.06
	EMD-43175	18	FP	8,905	additional map	Tecnai Spirit	2.06
	EMD-43175	18	GPC-A	12,945	main map	Tecnai Spirit	2.06
**Rabbit 188**	EMD-43176	30	Base	9,484	main map	Tecnai Spirit	2.06
**Rabbit 189**	EMD-43177	30	Base	12,037	additional map	Tecnai Spirit	2.06
	EMD-43177	30	GPC-A	13,250	main map	Tecnai Spirit	2.06
	EMD-43178	18	Base	5,061	additional map	Tecnai Spirit	2.06
	EMD-43178	18	GPC-A	2,937	main map	Tecnai Spirit	2.06
**Rabbit 190**	EMD-43179	30	Base	6,805	additional map	Tecnai Spirit	2.06
	EMD-43179	30	FP	11,371	main map	Tecnai Spirit	2.06
**Rabbit 191**	EMD-43180	30	Base	11,393	main map	Tecnai Spirit	2.06
**Rabbit 192**	EMD-43181	30	Base	15,413	additional map	Tecnai Spirit	2.06
	EMD-43181	30	FP	15,136	main map	Tecnai Spirit	2.06
**LAVA05**	EMD-43182	30	Base	17,515	main map	Tecnai Spirit	2.06
**LAVA06**	EMD-43183	30	Base	19,440	main map	Tecnai Spirit	2.06

Table S3: Cryo-EM data collection and atomic model refinement, related to Figures 3, 4, 5, and 6. Model refinement and validation was performed in Phenix 1.21.[5]

	GPC bound to mAb 8.9F	GPC bound to pAb GPC-A-2 (r189)	GPC bound to pAb GPC-A-1 (r187)	GPC bound to pAb LL-1	GPC bound to pAb Base-1
**Access codes**					
PDB	9CJ7	9CK8	9CK7	9CJ8	8TYC
EMDB	EMD-45624	EMD-45644	EMD-45643	EMD-45625	EMD-41713
Genbank	NP_694870.1	NP_694870.1	NP_694870.1	NP_694870.1	NP_694870.1
**Data collection and processing**					
Microscope	Titan Krios	TFS Glacios II	TFS Glacios	TFS Glacios	Titan Krios
Magnification	130,000	190,000	190,000	190,000	105,000
Voltage (kV)	300	200	200	200	300
Electron exposure (e$^-$/Å^2)	50.0	41.21	43.68	43.68	50.35
Defocus range (μm)	-0.7 to -2.0	-0.8 to -2.0	-0.8 to -2.0	-0.8 to -2.0	-0.7 to -2.0
Camera	Gatan K2 Summit	TFS Falcon 4i	TFS Falcon 4	TFS Falcon 4	Gatan K3 Bioquantum
Pixel size (Å)	1.045	0.718	0.725	0.725	0.833
Imposed Symmetry	C1	C1	C1	C1	C1
Final particle number	73,222	86,915 (symmetry expanded)	53,020 (symmetry expanded)	22,783 (symmetry expanded)	61,338 (symmetry expanded)
Map resolution (Å)	3.0	3.0	3.5	3.7	3.3
FSC Threshold	0.143	0.143	0.143	0.143	0.143
Map sharpening B-factor (Å^2)	-35	-69.9	-79.8	-57.5	-60
**Model refinement and validation**					
Total Residues	1389	1284	1293	1258	1353
Amino-acids	1300	1225	1235	1210	1273
Carbohydrates	89	59	58	48	80
RMSD Bonds	0.01	0.01	0.01	0.01	0.01
RMSD Angles	0.95	0.84	0.91	1.03	1.00
**Ramachandran**					
Outliers (%)	0	0	0	0	0
Allowed (%)	4.18	4.53	4.83	4.69	4.48
Favored (%)	95.82	95.47	95.17	95.31	95.52
Rotamer outliers (%)	0	0	0.11	0	0
Clash score	2.9	3.5	4.7	4.0	2.8
Molprobity score	1.4	1.5	1.6	1.5	1.4
FSC model (0/0.143/0.5)	2.7/3.0/3.3	2.9/3.0/3.2	3.3/3.4/3.8	3.6/3.7/4.0	3.0/3.3/3.7
EMRinger score	4.17	4.82	3.38	2.04	2.83

	GPC bound to pAb Base-2	GPC bound to pAb FP-1	GPC monomer bound to pAbs Int-1 and Int-2	GPC bound to pAb GPC-C-1	GPC bound to pAb GP1-A-1
**Access codes**					
PDB	NA	8TYE	N/A	8VCV	8VE8
EMDB	EMD-45905	EMD-41715	EMD-41716	EMD-43141	EMD-43168
Genbank	NP_694870.1	NP_694870.1	NP_694870.1	NP_694870.1	NP_694870.1
**Data collection and processing**					
Microscope	Titan Krios	TFS Glacios	TFS Glacios	TFS Glacios II	TFS Glacios II
Magnification	105,000	190,000	190,000	190,000	190,000
Voltage (kV)	300	200	200	200	200
Electron exposure (e$^-$/Å^2)	50.35	43.3	49.5	40.7	40.7
Defocus range (μm)	-0.7 to -2.0	-0.8 to -2.0	-0.8 to -1.4	-0.8 to -2.0	-0.8 to -2.0
Camera	Gatan K3 Bioquantum	TFS Falcon 4	TFS Falcon 4	TFS Falcon 4i	TFS Falcon 4i
Pixel size (Å)	0.833	0.725	0.725	0.718	0.718
Imposed Symmetry	C1	C1	C1	C1	C1
Final particle number	14,690 (symmetry expanded)	59,236 (symmetry expanded)	20,839	89,195	146,722
Map resolution (Å)	3.8	3.8	8.1	2.8	2.8
FSC Threshold	0.143	0.143	0.143	0.143	0.143
Map sharpening B-factor (Å^2)	-80	-80	-896	-58	-64
**Model refinement and validation**					
Total Residues		1273		1345	1329
Amino-acids		1212		1270	1260
Carbohydrates		61		75	69
RMSD Bonds		0.01		0.01	0.01
RMSD Angles		0.98		0.85	0.88
**Ramachandran**					
Outliers (%)	N/A	0	N/A	0	0
Allowed (%)		4.92		2.56	3.39
Favored (%)		95.08		97.44	96.61
Rotamer outliers (%)		0		0	0
Clash score		5.9		2.9	2.3
Molprobity score		1.7		1.2	1.2
FSC model (0/0.143/0.5)		3.7/3.8/4.1		2.7/2.8/3.0	2.7/2.7/2.9
EMRinger score		2.53		6.06	6.08

References

1. Li, H., Buck, T., Zandonatti, M., Yin, J., Moon-Walker, A., Fang, J., Koval, A., Heinrich, M.L., Rowland, M.M., Diaz Avalos, R., et al. (2022). A cocktail of protective antibodies subverts the dense glycan shield of Lassa virus. Sci. Transl. Med. *14*, eabq0991.
2. Monigatti, F., Gasteiger, E., Bairoch, A., Jung, E. (2002). The Sulfinator: predicting tyrosine sulfation sites in protein sequences. Bioinformatics *18*, 769-770
3. Punjani, A., Rubinstein, J.L., Fleet, D.J., and Brubaker, M.A. (2017). cryoSPARC: algorithms for rapid unsupervised cryo-EM structure determination. Nat. Methods *14*, 290–296.
4. Pettersen, E.F., Goddard, T.D., Huang, C.C., Meng, E.C., Couch, G.S., Croll, T.I., Morris, J.H., and Ferrin, T.E. (2021). UCSF ChimeraX: Structure visualization for researchers, educators, and developers. Protein Sci. *30*, 70–82.
5. Liebschner, D., Afonine, P.V., Baker, M.L., Bunkóczi, G., Chen, V.B., Croll, T.I., Hintze, B., Hung, L.W., Jain, S., McCoy, A.J., et al. (2019). Macromolecular structure determination using X-rays, neutrons and electrons: recent developments in Phenix. Acta Crystallogr D Struct Biol *75*, 861–877.
6. Katz, M., Weinstein, J., Eilon-Ashkenazy, M., Gehring, K., Cohen-Dvashi, H., Elad, N., Fleishman, S.J., and Diskin, R. (2022). Structure and receptor recognition by the Lassa virus spike complex. Nature *603*, 174–179.
7. Enriquez, A.S., Buck, T.K., Li, H., Norris, M.J., Moon-Walker, A., Zandonatti, M.A., Harkins, S.S., Robinson, J.E., Branco, L.M., Garry, R.F., et al. (2022). Delineating the mechanism of anti-Lassa virus GPC-A neutralizing antibodies. Cell Rep. *39*, 110841.
8. Montiel-Garcia, D., Rojas-Labra, O., Santoyo-Rivera, N., and Reddy, V.S. (2022). Epitope-Analyzer: A structure-based webtool to analyze broadly neutralizing epitopes. J. Struct. Biol. *214*, 107839.
