# Supplementary material for: Defining bottlenecks and opportunities for Lassa virus neutralization by structural profiling of vaccine-induced polyclonal antibody responses
Source: Cell Rep. 2024 Sep 6;43(9):114708. doi: 10.1016/j.celrep.2024.114708 (PMC11422484; doi:10.1016/j.celrep.2024.114708)
Supplement: Document S2. Article plus supplemental information [file mmc2.pdf]

Cell Reports

Article

Defining bottlenecks and opportunities for Lassa virus neutralization by structural profiling of vaccine-induced polyclonal antibody responses

Graphical abstract

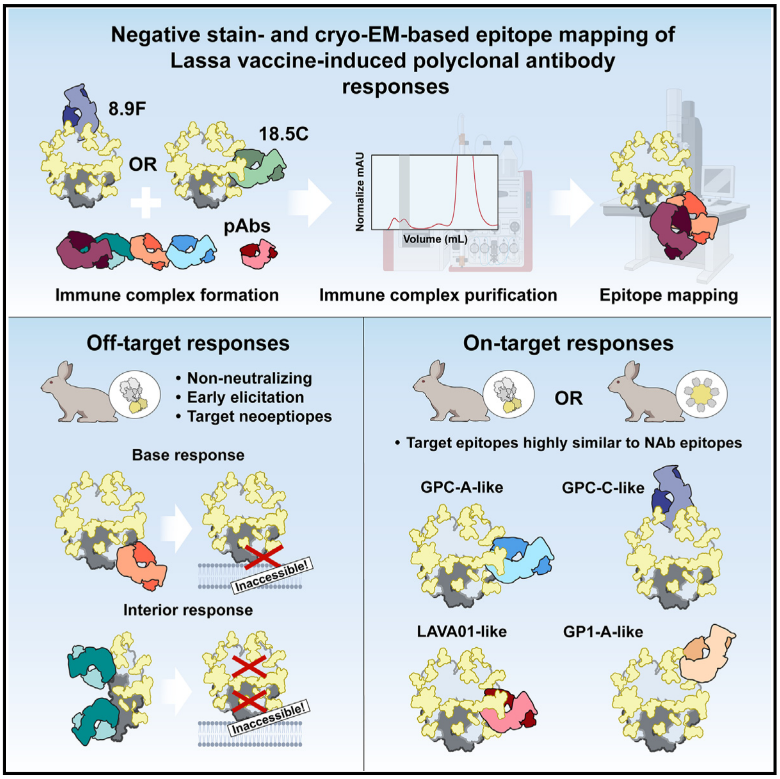

Highlights

- Tyrosine sulfation is employed by mAb 8.9F to target the GPC apex
- Polyclonal epitopes of immunized rabbits are mapped using electron microscopy
- Recombinant GPC elicits off-target responses aimed at the trimer base and interior
- VLP-embedded GPC elicits responses closely resembling neutralizing mAbs

Authors

Philip J.M. Brouwer, Hailee R. Perrett, Tim Beaumont, ..., Thomas Strecker, Marit J. van Gils, Andrew B. Ward

Correspondence

andrew@scripps.edu

In brief

Brouwer et al. develop a pipeline to map polyclonal antibody responses to the Lassa virus glycoprotein using electron microscopy. By applying this to serum from rabbits vaccinated with different vaccine modalities, they reveal and structurally characterize vaccine-specific off- and on-target antibody responses to the Lassa virus glycoprotein.

Check for updates

Brouwer et al., 2024, Cell Reports *43*, 114708
September 24, 2024 © 2024 The Author(s). Published by Elsevier Inc.
https://doi.org/10.1016/j.celrep.2024.114708

CellPress

Cell Reports

CellPress OPEN ACCESS

Article

Defining bottlenecks and opportunities for Lassa virus neutralization by structural profiling of vaccine-induced polyclonal antibody responses

Philip J.M. Brouwer,[1,5] Hailee R. Perrett,[1,5] Tim Beaumont,[2] Haye Nijhuis,[2] Sabine Kruijer,[2] Judith A. Burger,[2] Ilja Bontjer,[2] Wen-Hsin Lee,[1] James A. Ferguson,[1] Martin Schauflinger,[3] Helena Müller-Kräuter,[3] Rogier W. Sanders,[2,4] Thomas Strecker,[3] Marit J. van Gils,[2] and Andrew B. Ward[1,6,*]

[1]Department of Integrative Structural and Computational Biology, Scripps Research, La Jolla, CA 92037, USA

[2]Department of Medical Microbiology and Infection Prevention, Amsterdam University Medical Center, Location AMC, University of Amsterdam, Amsterdam Infection & Immunity Institute, 1105 AZ Amsterdam, the Netherlands

[3]Institute of Virology, Philipps University Marburg, 35043 Marburg, Germany

[4]Department of Microbiology and Immunology, Weill Medical College of Cornell University, New York, NY 10021, USA

[5]These authors contributed equally

[6]Lead contact

*Correspondence: andrew@scripps.edu

https://doi.org/10.1016/j.celrep.2024.114708

SUMMARY

Lassa fever continues to be a major public health burden in West Africa, yet effective therapies or vaccines are lacking. The isolation of protective neutralizing antibodies against the Lassa virus glycoprotein complex (GPC) justifies the development of vaccines that can elicit strong neutralizing antibody responses. However, Lassa vaccine candidates have generally been unsuccessful at doing so, and the associated antibody responses to these vaccines remain poorly characterized. Here, we establish an electron microscopy-based epitope mapping workflow that enables high-resolution structural characterization of polyclonal antibodies to the GPC. By applying this method to rabbits vaccinated with a recombinant GPC vaccine and a GPC-derived virus-like particle, we reveal determinants of neutralization that involve epitopes of the GPC-A competition cluster. Furthermore, by identifying undescribed immunogenic off-target epitopes, we expose the challenges that recombinant GPC vaccines face. By enabling detailed polyclonal antibody characterization, our work ushers in a next generation of more rational Lassa vaccine design.

INTRODUCTION

Lassa fever, a viral hemorrhagic fever caused by the Lassa virus (LASV), is a persistent public health and socioeconomic burden to affected countries in West Africa. LASV is predominantly transmitted to humans from its natural reservoir *Mastomys natalensis*[1] (though person-to-person transmission does occur[2–4]), infecting an estimated 500,000–900,000 people each year and resulting in approximately 5,000 deaths.[5,6] While many people infected with LASV have asymptomatic or mild infections, those that present with clinical symptoms suffer from fever, encephalitis, respiratory distress, facial swelling, bleeding from orifices, and multiorgan failure, which, without early treatment, may result in death.[7] Even in cases of mild Lassa fever, approximately one-third of those infected experience sensorineural hearing loss, which results in socioeconomic strain in endemic regions.[8–10] Further, current estimates suggest that those affected by the disease while pregnant have almost 3-fold higher fatality rates and that 80% of these patients experience intrauterine fetal deaths.[11] With no effective and approved therapeutic or vaccine, the World Health Organization has classified Lassa fever as a priority disease.[12] The development of a vaccine that can protect against this devastating pathogen would be a major public health advancement.

Lassa vaccine efforts focus primarily on the LASV glycoprotein complex (GPC). Not only does the GPC harbor numerous T cell epitopes,[13,14] it is also the sole mediator for host cell infection and presents all known neutralizing antibody (NAb) epitopes.[15] The GPC resides on the viral surface as a trimer of GP heterotrimers, with each GPC protomer comprised of the receptor-binding subunit GP1, the transmembrane-spanning subunit GP2, and the stable signal peptide (SSP), which remains non-covalently attached to GPC after it is cleaved from the GPC precursor.[16–19] The prefusion GPC facilitates the infection of host cells by engaging with its primary extracellular receptor, matriglycan.[20–22] After internalization and trafficking to the endosome via macropinocytosis,[23] GP1 undergoes a conformational change in response to the endosome's acidic pH, which enables its binding to the endosomal receptor, lysosomal-associated membrane protein 1 (LAMP-1).[24,25] LAMP-1 binding facilitates the GPC's conformational shift from a pre- to a post-fusion state and promotes viral and host membrane fusion.[26] The

This is an open access article under the CC BY license (http://creativecommons.org/licenses/by/4.0/).

conformational lability of GPCs, required to undergo these dramatic structural changes, has frustrated the development of stable recombinant prefusion GPCs for many years. The introduction of prefusion-stabilizing GPCysR4 mutations[27]—R207C and G360C to introduce a disulfide bond between GP1 and GP2, the helix-breaking E329P, and L258R and L259R to replace the site-1 protease (S1P) cleavage site with a furin cleavage site—combined with the complexing of a trimer-stabilizing monoclonal Ab (mAb), 37.7H, finally enabled the generation and high-resolution structural characterization of prefusion GPC trimers.[27,28] Since then, methods have been described to stabilize the trimeric conformation of the prefusion GPC in the absence of a trimer-stabilizing mAb.[29–31] We previously demonstrated that prefusion GPC trimers are stabilized by genetic fusion to the trimeric scaffold I53-50A.[30,31] These fusion proteins, known as GPC-I53-50A, have not only proven to be a useful reagent for characterizing and isolating GPC-specific Abs but were also able to induce NAb responses in rabbits.[30,31]

Protective immunity to LASV infection has been attributed to cell-mediated responses,[5] with severe infections often associated with poor T cell responses to the GPC and nucleoprotein.[14,32,33] NAb responses appear to play a limited role in curbing acute infection as they generally appear months after viral clearance.[34] The GPC's dense glycan shield and the elicitation of non-NAbs against the post-fusion or uncleaved conformations of the GPC likely hinder the induction of NAbs.[35–38] In addition, by a mechanism that remains unknown, LASV delays immunoglobulin (Ig) class switching of IgM to IgG, further impeding the maturation of NAb responses.[39] Nevertheless, numerous potent NAbs have been isolated from convalescent individuals, and the mechanism of action of several of these NAbs has been elucidated.[15,27–29,31,40,41] Cocktails of isolated NAbs have shown great promise as therapeutics in animal models. For example, Arevirumab-3, a cocktail of NAbs 8.9F, 37.2D, and 12.1F, shows protection in non-human primates after LASV challenge.[42,43] These early successes justify pursuing the development of vaccines that are capable of inducing potent NAb responses.

Recent efforts in Lassa vaccine design include an mRNA vaccine; a DNA-based vaccine[44]; measles-, rabies-, adenovirus-, and vesicular stomatitis virus (VSV)-based vector vaccines[45–50]; a nanoparticle-based protein vaccine[30]; recombinant protein vaccines[29,30,51]; and virus-like particle (VLP) vaccines.[52,53] While many of these have shown efficacy in animal models, the induction of NAbs, in most cases, was absent or required numerous boosting immunizations. The reasons for this may vary between vaccine platforms but may include the possible presentation of irrelevant GPC conformations to B cells. Nevertheless, careful and robust assessments of vaccine-induced Ab responses have been lacking. As a result, it remains unclear which epitopes are being targeted on the highly glycosylated GPC. Identifying the vaccine-induced on- and off-target responses and understanding their place in the immunodominance hierarchy will be an important first step in designing next-generation LASV vaccines that induce more potent NAb responses.

In addition to traditional assays to assess humoral immune responses, electron microscopy (EM)-based polyclonal epitope mapping (EMPEM) has been used successfully to add low- and/or high-resolution structural definition to polyclonal Ab (pAb) immune responses across several pathogens.[54–58] In short, serum Abs from immunized or challenged individuals can be purified, digested to Fab, complexed with antigen of interest, and directly visualized using EM. This methodology is valuable for determining the presence and immunodominance of responses across time. Here, we describe the development and in-depth characterization of a next-generation GPC-I53-50A trimer and use it to establish an EMPEM workflow for LASV. We then apply this method to investigate the pAb responses from rabbits previously immunized with GPC-I53-50A as well as a GPC-derived VLP.[30,53] Our analysis identifies a range of epitopes that were targeted by pAbs, including neutralizing epitopes of the GPC-A competition group. In addition, we describe off-target, non-neutralizing epitopes at the interior and base of the recombinant GPC trimer. By generating high-resolution structural models of the epitope-paratope interfaces of vaccine-induced Ab responses, we provide a detailed landscape of the immunogenic sites on GPCs in different vaccine contexts. Our EMPEM work not only reveals the challenges that recombinant LASV vaccines face in inducing NAb responses but also informs the design of the next iteration that may overcome them.

RESULTS

Development and characterization of recombinant LASV GPC trimers that bind 8.9F

We recently showed that fusing GPCysR4 to I53-50A resulted in the generation of prefusion GPC trimers that present epitopes of the GP1-A, GPC-A, and GPC-B competition clusters; however, these immunogens still lacked the ability to bind the 8.9F mAb, the sole known member of the GPC-C cluster. This highly potent broadly NAb (bNAb) directly blocks matriglycan binding by receptor mimicry and requires the native cleavage site to engage with the GPC.[16,29] Therefore, to generate a trimeric GPC immunogen that presents all currently known bNAb epitopes, we reverted the RRRR site of GPCysR4 to the native RRLL sequence. The resulting construct, GPCysRRLL-I53-50A, was expressed in Freestyle 293F cells by co-transfection with S1P and purified by subsequent Strep-Tactin XT affinity chromatography and size-exclusion chromatography (SEC) steps (Figure 1A). Sodium dodecyl sulfate polyacrylamide gel electrophoresis (SDS-PAGE) demonstrated that these proteins were efficiently cleaved (Figure S1A), while negative stain EM (nsEM) confirmed that GPCysRRLL-I53-50A forms trimers (Figure 1B). Furthermore, biolayer interferometry (BLI) showed GPCysRRLL-I53-50A binding to representative bNAbs of the GP1-A (12.1F), GPC-A (25.10C), and GPC-B (37.7H) epitope clusters (Figure S1B) while restoring its ability to bind 8.9F (Figure 1C).

Next, to characterize GPCysRRLL-I53-50A in molecular detail, we used single-particle cryoelectron microscopy (cryo-EM) to solve a 3.0 Å structure of GPCysRRLL-I53-50A in complex with the 8.9F Fab (Figure 1D). Our GPC model shows a near-identical structural homology to that of a previously described detergent-solubilized full-length native GPC complexed with 37.2D and 8.9F Fab, as measured by the root-mean-square deviation

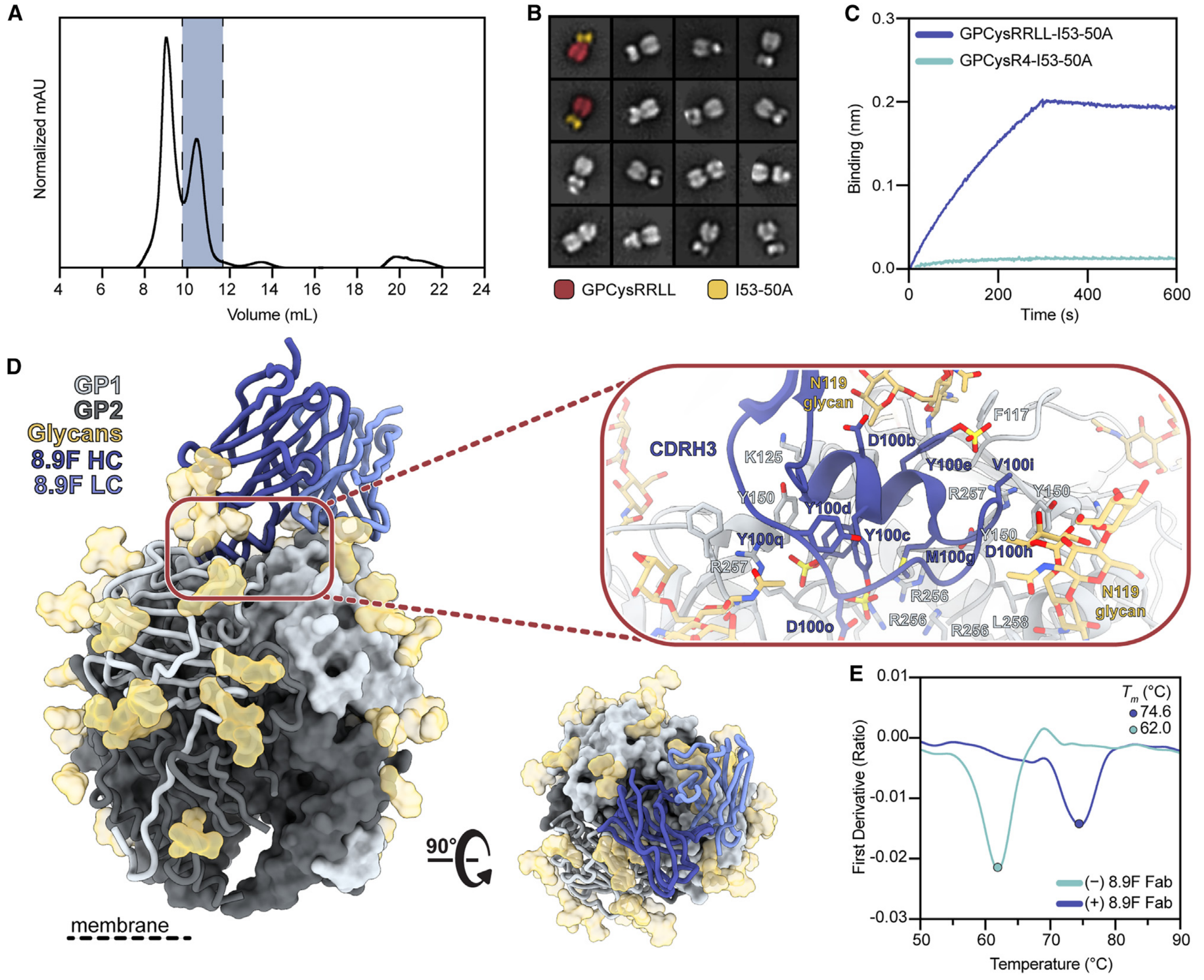

Figure 1. Development and characterization of GPCysRRLL-I53-50A and binding to 8.9F

(A) Representative SEC of GPCysRRLL-I53-50A. Fractions containing trimers are shown in gray.

(B) 2D class averages from nsEM of the GPCysRRLL-I53-50A. The far left GPCysRRLL-I53-50As are pseudocolored and show the GPCysRRLL (red) and I53-50A (gold) scaffolds.

(C) Representative BLI sensorgram comparing binding of immobilized 8.9F IgG to GPCysRRLL-I53-50A (blue) and GPCysR4-I53-50A (cyan). Single curves are shown that are representative of three technical replicates.

(D) Atomic model of GPCysRRLL-I53-50A bound to 8.9F Fab (blue) determined by cryo-EM. Inset depicts key interactions between GP1 and 8.9F at the epitope-paratope interface. Glycan N119, which makes extensive contact with the heavy and light chains, is shown in yellow. More details on the epitope-paratope interactions are presented in Table S1.

(E) Thermostability of GPCysRRLL-I53-50A with and without 8.9F Fab bound assessed by nanoDSF. Melting temperatures (T_m) are calculated as the inflection point (circles) of the first derivative of the ratios of signal at 350 and 330 nM. Each melting curve is a representative of triplicate curves with T_m within ±0.1°C.

See also Figure S1 and Table S1.

(RMSD; 0.53 Å among the full 8.9F sequence and 190 pruned atom pairs per GP1; 1.0 Å overall fit across all sequence-aligned pairs with PDB: 7UOT[29]). Consistent with earlier observations, 8.9F targets the trimer apex, making contact with all three protomers while using its 31 aa CDRH3 to penetrate the 3-fold axis in between the α1 helix and the complex-type glycans at position N119 (Figure 1D; Table S1). Meanwhile, the light chain (LC) interacts with the neighboring protomer, principally making contacts with the N119 glycan. Despite clear overlap between the two models, we note a key difference. Closer inspection of both maps revealed well-resolved additional density beyond the hydroxyl group of tyrosines 100C, 100D, and 100E (Kabat numbering scheme), suggesting the presence of sulfate groups (Figure S1C). The presence of sulfated tyrosines at these positions was corroborated by running the 8.9F heavy-chain (HC) sequence through Sulfinator, a bioinformatics tool that predicts tyrosine sulfation in protein sequences (Figure S1D).[59] Thus, in contrast to the previously described structure, these post-translational modifications are present in our 8.9F

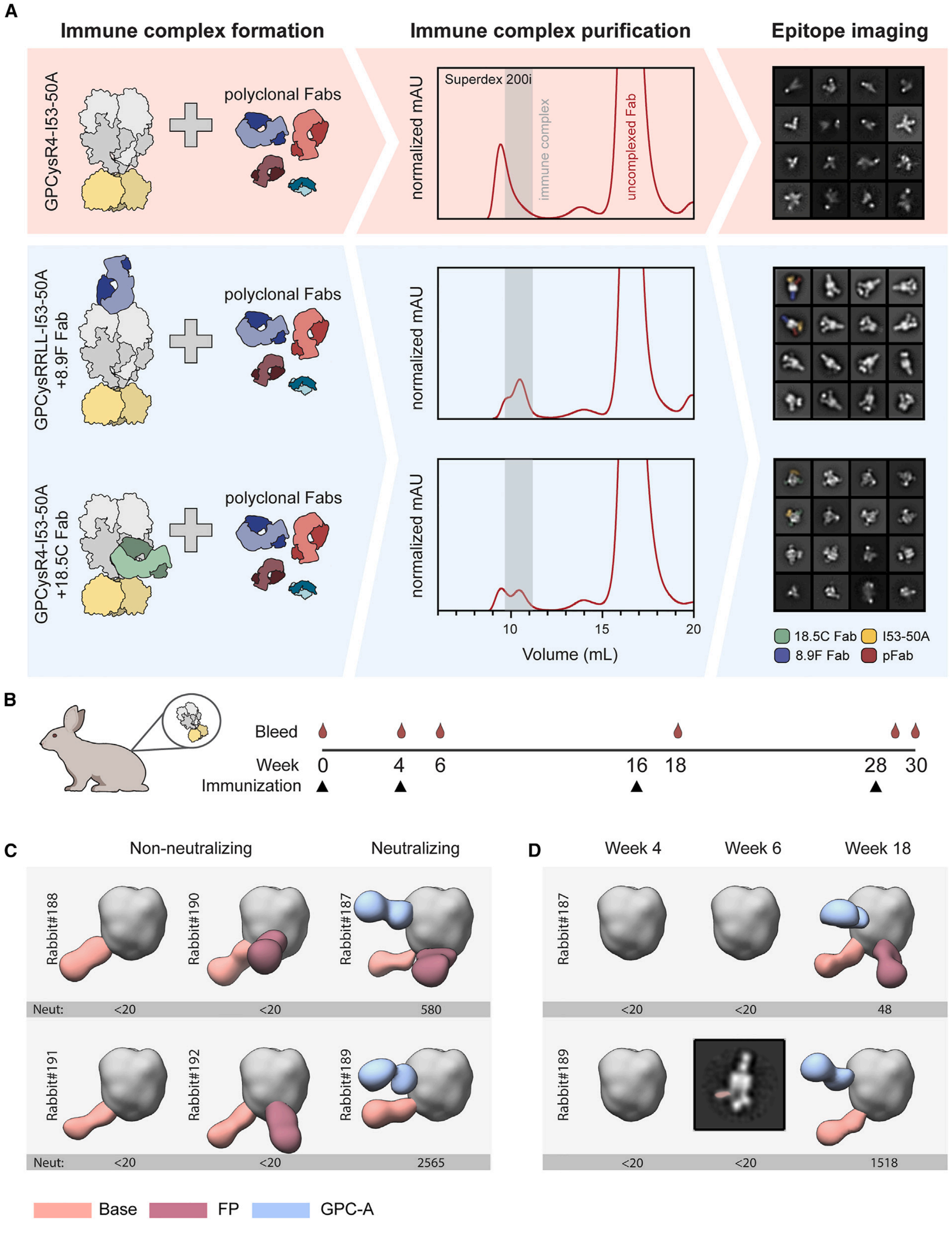

(legend on next page)

model. An additional difference between the models was at residues 204–214 in GP1, where the presence of the introduced prefusion-stabilizing disulfide bond in GPCysRRLL-I53-50A leads to an alternative conformation of this flexible loop.

Considering the way 8.9F engages with the RRLL sequence on all three protomers of GPC, we hypothesized that 8.9F may have a stabilizing effect on recombinant GPC trimers. Indeed, nano differential scanning fluorimetry (nanoDSF) studies showed that the thermostability of GPCysRRLL-I53-50A increases by 12°C when complexed with 8.9F (melting temperature [T_m] = 62°C for GPCysRRLL-I53-50A and 74°C for GPCysRRLL-I53-50A-8.9F Fab; Figure 1E). Interestingly, in contrast to earlier suggestions that the leucines of the S1P cleavage site stabilize the trimeric interface,[16] we did not observe an increase in thermostability of GPCysRRLL-I53-50A compared to GPCysR4-I53-50A (Figure S1E). Overall, we conclude that we have developed a new generation of prefusion-stabilized trimeric GPC-I53-50A with an improved antigenic profile.

Development of an EMPEM protocol for LASV GPC trimers

EMPEM is a powerful method to map antigen-specific pAb responses induced by infection or vaccination.[54] While EMPEM studies of HIV-1,[54,55,58,60–63] influenza,[57,64] and coronavirus[56] vaccine-induced Ab responses have boosted further vaccine development, the Ab profile induced by Lassa vaccines remains ill-defined. For our initial attempts to map GPC-specific polyclonal responses, we used pools of purified polyclonal Fabs (pFabs) from rabbits that were previously immunized with GPCysR4-I53-50A trimers.[30] Overnight incubations of GPCysR4-I53-50A with an excess of rabbit Fabs resulted in the formation of immune complexes, as shown by the shift in the SEC trace of GPCysR4-I53-50A. However, when the corresponding fractions were pooled, imaged by nsEM, and subjected to single-particle extraction and 2D classification, only classes of GPC monomers (i.e., a splayed-open GPC held together by the I53-50A scaffold) were discernible-. To assess if this issue could be mitigated by incubating for a shorter time period, we generated complexes and incubated for 4 h. Again, 2D class averaging of extracted single particles did not generate classes that represented trimeric GPC-pFab complexes (Figure 2A, top right). These results suggest that pAbs cause the disassembly of our recombinant GPC trimers.

Considering 8.9F's effect on the thermostability of GPC trimers, we rationalized that complexes of 8.9F Fab with GPCysRRLL-I53-50A may be able to withstand the Fab-induced disassembly and prove to be a useful reagent for EMPEM studies. Indeed, when we performed single-particle analysis from nsEM studies with GPCysRRLL-I53-50A-8.9F complexed with rabbit pFabs, we were able to obtain a sufficient amount of 2D class averages that represented Fab-bound GPC trimers (Figure 2A, middle right). As using GPCysRRLL-I53-50A-8.9F complexes to probe pAb responses to GPC occludes potential apex-targeting pAbs, we also performed EMPEM with the same pFabs using GPCysR4-I53-50A complexed with the trimer-stabilizing Fab 18.5C (Figure 2A, bottom). Here, GPCysR4-I53-50A was chosen instead of GPCysRRLL-I53-50A so that it matched the vaccine sequence. Although in this context, pFab binding was not observed, the trimers clearly withstood pFab-induced disassembly. We thus present a two-pronged approach for LASV EMPEM: (1) precomplexing GPCysRRLL-I53-50A with the 8.9F Fab to sample pFab responses excluding those targeting the apex and (2) precomplexing with the 18.5C Fab to sample pFabs that target the apex (Figure 2A).

Analysis of recombinant LASV GPC-induced Ab responses: The base epitope

Having established an EMPEM protocol, we continued to apply it to all six rabbits that were previously immunized with GPCysR4-I53-50A.[30] These rabbits received an intramuscular immunization of GPCysR4-I53-50A formulated in squalene emulsion adjuvant at weeks 0, 4, 16, and 28 (Figure 2B).[30] Two of these rabbits induced pseudovirus neutralization titers and four did not (Figure 2C), enabling us to correlate neutralization with mapped epitopes. The resulting 3D representations of the GPC with bound pFabs from week 30 revealed a remarkably consistent response targeting the GPC base (Figures 2C and S2A; Table S2). These Abs were present in all rabbits, regardless of whether they induced neutralizing responses, and thus may represent a common but non-neutralizing response to recombinant GPC immunogens. To understand the evolution of these responses during the prime-boosting strategy, we performed EMPEM experiments with week 4, 6, and 18 serum samples from rabbits 187 and 189 (Figures 2D and S2B; Table S2). Base-targeting responses were the only responses discernible at week 6 (after secondary immunization), albeit at very low levels and in only one out of two rabbits.

To obtain more detailed structural information on the epitope-paratope interface of these base-targeting responses, we performed single-particle cryo-EM on GPC-pFab complexes from rabbit 190. We reconstructed two maps of structurally unique

Figure 2. Stabilizing Abs facilitate LASV GPC EMPEM and generation of epitope landscapes

(A) Schematic of LASV GPC EMPEM development. In the absence of stabilizing Fabs, GPC undergoes pFab-facilitated disassembly, which leads to aggregation as determined by SEC and splayed open GPC trimers as visualized by nsEM (red box). In the presence of stabilizing Fabs 8.9F and 18.5C, immune complexes are formed, and the pFab response against GPC trimers can be visualized, enabling the mapping of the pAb landscape (blue box).

(B) Immunization scheme of the six rabbits immunized at week 0, 4, 16, and 28 with GPCysR4-I53-50A antigen, as recently described.[30]

(C and D) Composite figures of pFab responses of each individual rabbit as determined by nsEMPEM. To the left of each image, the rabbit identifier is shown, and the previously described ID_{50} of LASV pseudovirus serum neutralization is depicted on the bottom.[30] A titer of <20 represents no neutralization. pFabs are color-coded based on their apparent epitope as shown in the legend. For simplicity, only a single Fab is shown and consistently projected on the same protomer. (C) Binding of pFabs at week 30. (D) Binding of pFabs at weeks 4, 6, and 18 for rabbits 187 and 189. Because of limited numbers of particles that contain a base response, we were unable to reconstruct a 3D map for the week 6 pFab response in rabbit 189. Instead, a 2D class is shown, with the base pFab highlighted by pseudocoloring.

See also Figure S2 and Table S2.

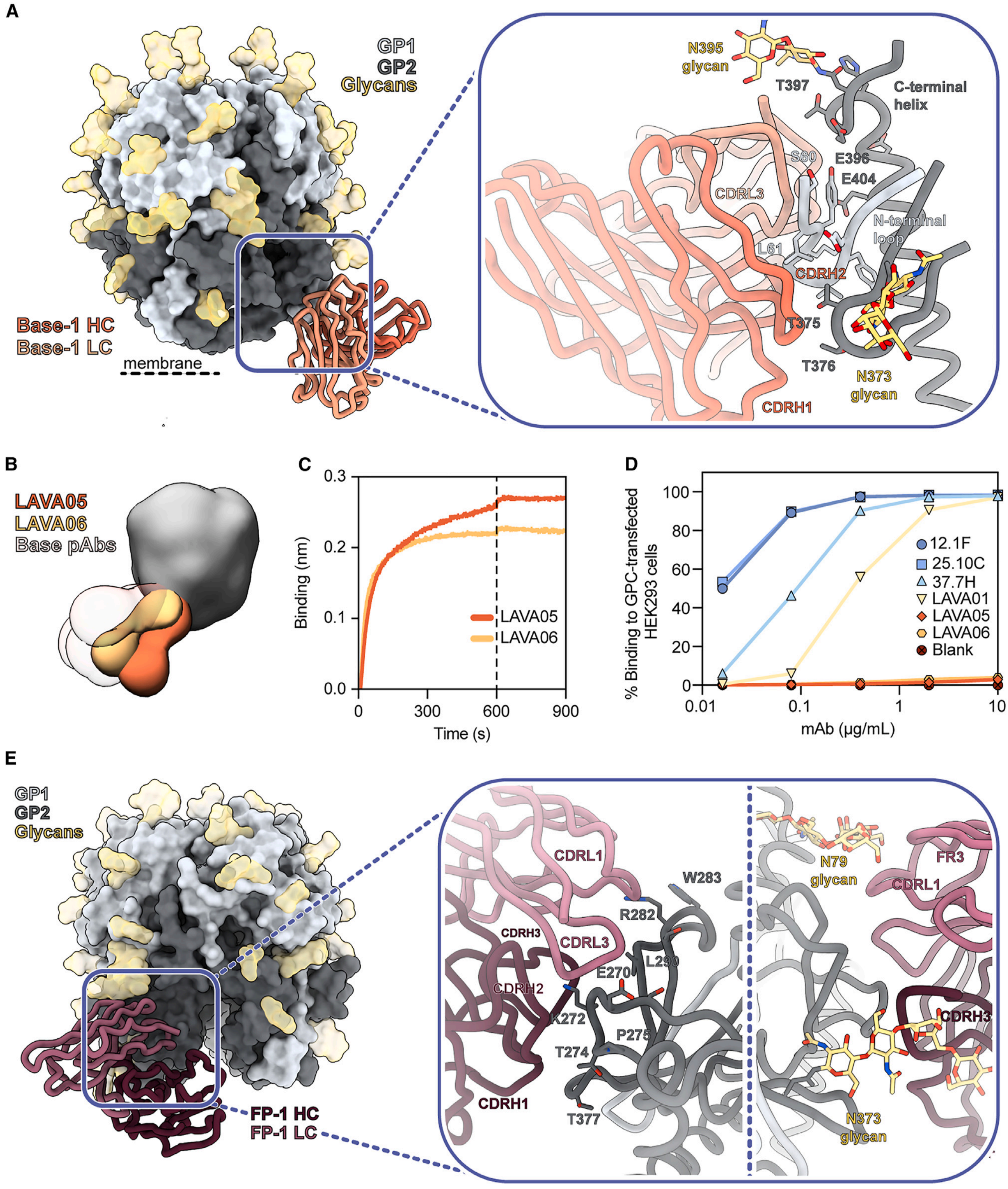

Figure 3. Recombinant LASV GPC immunization in rabbits induces Ab responses to the GPC base and fusion peptide

(A) Atomic model of Base-1 pFab bound to GPCysRRLL-I53-50A as determined by cryo-EM. Inset depicts presumed interactions between the modeled poly-alanine pFab model and the GPC. Residues depicted as sticks on the GPC are within 6 Å of the poly-alanine chain and, collectively, are considered the epitope footprint.

(legend continued on next page)

GPC-pFab complexes, with each map revealing a pFab targeting the GPC base (Figures S3 and S4; Table S3). Of the two maps, corresponding to pFabs we named Base-1 and Base-2, the former had sufficient resolution to guide the initial placement of conserved amino acids. This enabled us to relax previously deposited rabbit Fab models and define CDR loops with greater confidence.[30,65] After initial placement and relaxation, the models' amino acids were redefined as poly-alanine to account for our lack of sequence information (Figure 3A). Our model showed that Base-1 targets the N-terminal loop of GP1 (aa 59–67) and several residues at the beginning of the C-terminal helix of GP2 (aa 395–419). The N-terminal loop is engaged by the pFabs' CDRH1, CDRH2, CDRH3, and CDRL1, whereas the C-terminal helix is targeted using its CDRL1 and CDRL2. Furthermore, Base-1 seemed to make contact with both N373 and N395 glycans. Despite insufficient resolution of the Base-2 map for model building, a docked rabbit Fab model (PDB: 7RA7) in the pFab density revealed a clearly distinct angle of approach compared to base-1 (Figure S5A). With respect to the C-terminal helix, Base-1 approaches the GPC in a near-horizontal plane, while Base-2 is tilted approximately 80° and binds at a much steeper angle (Figure S5B). These differences imply that the two pFabs originate from different germline B cells, which aligns with the relative immunogenicity of this epitope.

Superimposition of the models of Base-1 with a model of a full-length GPC (PDB: 7PUY[16]) suggested that this base-targeting response may not be elicited when the GPC is embedded in the membrane. This response would likely be blocked by the lipid bilayer in the context of native virions. Additionally, if the membrane-embedded GPCs were able to tilt, then binding of this pFab to the base epitope could be sterically hindered by the non-covalently bound SSP (Figure S5C). To investigate if the base epitope is accessible on the membrane-embedded GPC, we isolated two mAbs that shared the same HC sequence: LAVA05 and LAVA06. These mAbs bound the GPC in a very similar fashion to the base-targeting responses observed in rabbit sera (Figures 3B and S5D; Table S2). Despite their strong binding to the GPC (Figure 3C), LAVA05 and LAVA06 did not neutralize the LASV pseudovirus (Figure S5E), consistent with our EMPEM observations that base responses do not correlate with neutralization (Figure 2C). Furthermore, while rabbit NAb LAVA01 and human NAbs 12.1F, 25.10C, and 37.7H showed binding to the GPC embedded on the membrane of transfected HEK293 cells, LAVA05 and LAVA06 did not (Figure 3D). These data, together with our EMPEM structures, suggest that the base constitutes a highly immunogenic neoepitope that is made accessible when generating recombinant GPC trimers.

Analysis of recombinant LASV GPC-induced Ab responses: The fusion peptide epitope

Cryo-EM analysis of the polyclonal response in rabbit 190 enabled us to provide higher-resolution detail of a response observed in 3 out of 6 rabbits that targeted an epitope on the opposite side of the GPC relative to Base-1 (Figures 3E, S3, and S4; Table S3). Here, the pFab, designated FP-1, makes contact with parts of the fusion peptide (aa 260–275) and fusion loop (aa 276–295; Figure 3E). Recent studies have shown that multiple conformations of the fusion peptide are possible, with the fusion peptide in unbound GPCs (absence of matriglycan or Abs) residing flexibly in the space between the HR1a helix, HR1d helix, and HR2 loop.[30,31] We note that binding by FP-1 seems to promote the fusion peptide conformation where residues 260–268 extend toward the interior trimeric interface, as has been observed when the GPC is bound by other NAbs, such as 25.10C and 37.7H. Our map and model suggests FP-1 targets residues 271–273 through interactions with each CDR of FP1's HC, while the LC seems to contact residues 270 and 271 through its CDRL3 and 281, 282, 283, and 290 through its CDRL1. While its peptidic interactions are localized to the GP2 subunit, the N79 glycan seemingly has numerous points of contact with the LC framework region (FR)3, with contacts likely extending to the CDRL1 and CDRL2. Other putative glycan contacts are observed between the N373 glycan and the CDRH3 loop. FP-1 binds the GPC in a loosely comparable way to GPC-B-specific Abs such as 37.7H and would undoubtedly fall in the GPC-B competition cluster (Figure S5F). However, FP-1 only targets one protomer and focuses primarily on the fusion peptide and fusion loop. In contrast, GPC-B Abs typically have a footprint that covers both protomers and engage the fusion peptide rather than the fusion loop, keeping it "locked" into the pocket formed by the C-terminal helix, the HR1d helix, and the flexible loop upstream of β11 (Figure S5G).[27,28] These differences may provide a structural explanation to why pAbs from serum 190—unlike GPC-B-specific mAbs 37.7H, 18.5C, and 25.6A—did not induce measurable neutralization.

Analysis of recombinant LASV GPC-induced Ab responses: The GPC-A and LL epitopes

Beyond the omnipresent base responses, Fab densities targeting equatorial epitopes were noticeable from the week 30 nsEMPEM data (Figure 2C). Interestingly, these responses were only present in the two rabbits that generated NAbs (rabbits 187 and 189), suggesting that these pAbs might be neutralizing (Figure 2C). Longitudinal EMPEM analyses with serum from weeks 4, 6, and 18 revealed that Abs against these equatorial epitopes are only being elicited after three immunizations at week 18

(B) Composite figure of LAVA05 and LAVA06 Fab binding to GPC as determined by nsEM. Base-targeting pFab responses from rabbits 187–192 are presented with transparent coloring to display the overlap between the mAbs and pAbs.

(C) Representative sensorgrams from BLI experiments measuring binding of LAVA05 and LAVA06 to GPCysRRLL-I53-50A captured by His-tagged 8.9F Fab immobilized on Ni-NTA biosensors.

(D) Binding of mAbs to full-length native GPC expressed on HEK293 cells. MAbs bound to GPC-expressing cells were detected by flow cytometry using PE-labeled anti-rabbit/human IgG. The percentages of fluorescently labeled cells are depicted as a function of the mAb concentration.

(E) Atomic model of FP-1 Fab bound to GPCysRRLL-I53-50A as determined by cryo-EM. Inset depicts presumed interactions between the modeled poly-alanine pFab model and the GPC. Peptidic contacts are shown on the left and glycan contacts on the right of the inset. Residues depicted as sticks on the GPC are within 6 Å of the poly-alanine chain and, collectively, are considered the epitope footprint.

See also Figures S3–S5 and Tables S2 and S3.

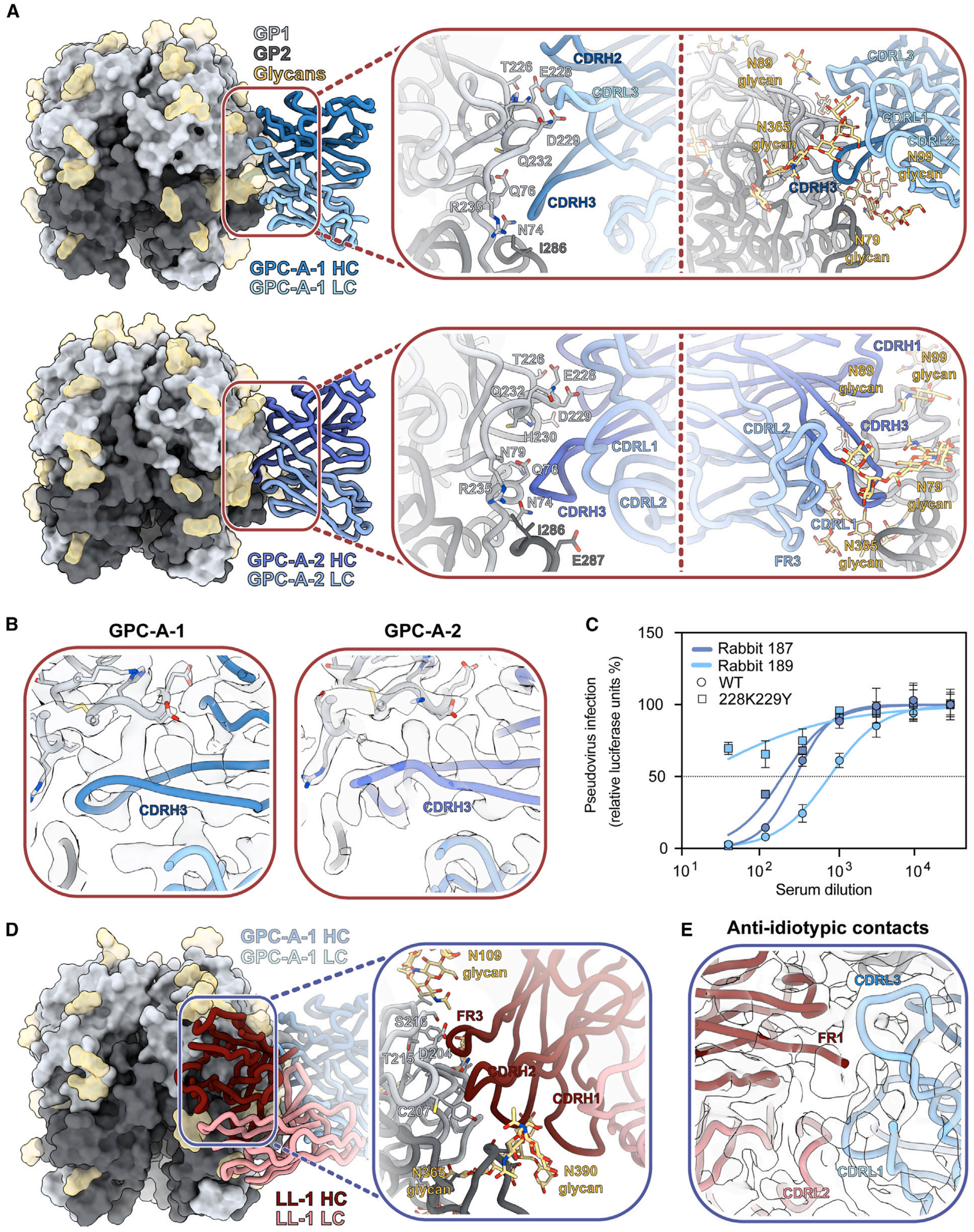

Figure 4. Recombinant LASV GPC immunization in rabbits induces Ab responses to the GPC-A and LL epitope

(A) Atomic model of GPC-A-1 pFab (top) and GPC-A-2 pFab (bottom) bound to GPCysRRLL-I53-50A determined by cryo-EM.

(B) Closeup of the map and model of GPC-A-1 (left) and GPC-A-2 (right), illustrating the similarity between their respective CDRH3s.

(legend continued on next page)

(Figure 2D). Week 18 is also when we see the emergence of pseudovirus NAb titers (Figure 2D), further strengthening the correlation between these equatorial responses and neutralization.

Cryo-EM analyses of immune complexes from rabbits 187 and 189 week 30 serum (Figures 4A, S3, and S4; Table S3) resulted in maps of two pFabs, which we named GPC-A-1 (3.0 Å) and GPC-A-2 (3.4 Å), from rabbits 187 and 189, respectively. Models of these pFabs show that they engage the GPC in a manner reminiscent of GPC-A-specific NAbs 36.1F and, to a lesser extent, 25.10C (Figures 4A and S6A).[40] Similar to 36.1F, the CDRH3 loops of these pFabs (17 aa in rabbit 187, 19 aa in rabbit 189) protrude in between glycans N79 and N365 to reach residue N74 in η1 while making extensive contact with residues in the flexible loop between residues 226 and 235. We note a marked similarity between the CDRH3 loops of GPC-A-1 and GPC-A-2, suggesting a convergent strategy to breach the dense glycan shield of the GPC (Figure 4B). The loop between residues 226 and 235 is further engaged by the CDRH1, CDRH2, and CDRL3 in both pFabs. Clear interactions were observed in the electron density maps with N74, W227, E228, D229, and Q232, which constitute contacted residues shared by the GPC-A-targeting NAbs 36.1F and 25.10C (Figure S6B).[40] Beyond peptidic contacts, GPC-A-1 and GPC-A-2 engage glycans N365 and N79 (Figure S6C). Interactions with N89 glycan seem likely as well, although the density was too diffuse to model past the primary N-acetyl glucosamine.

To confirm that GPC-A pAbs drive neutralization in these rabbits, we generated a Josiah pseudovirus mutant with GPC mutations E228K and D229Y. These mutations fully knocked out 25.10C neutralization while having no negative effect on the neutralization potency of mAbs from the other competition clusters (Figure S6D). Furthermore, serum neutralization by rabbit 189 was almost fully lost with this pseudovirus mutant, confirming that GPC-A-2 is indeed the primary neutralizing response in this rabbit (Figure 4C). For rabbit 187, only a minor decrease in neutralization was observed, suggesting that either our mutant was largely ineffective at destabilizing the epitope-paratope interface or that another response is driving neutralization. For example, an additional equatorial response that we were unable to reconstruct by nsEM but that was clearly noticable in a subset of particle classes from cryo-EM (Figures S3 and S4; Table S3) may have been responsible for the neutralization. This pFab targets an epitope that is similar to that of the previously isolated rabbit NAb, LAVA01, and is aptly designated LAVA01-like-1 (LL-1). LL-1 uses its CDRH1, CDRH2, and HC FR3 to interact with parts of the loop (aa 200–207 and 214–217) that connect η4 with β8 (Figure 4D).[30] In addition, this pFab likely engages residues 90, 223, and 391, as well as glycans N109 and N390. Superimposing the models of LL-1 and GPC-A-1 indicated that the two pFabs may interact with one another. Indeed, a map generated to include both pFabs showed clear interactions between the HC FR1 and CDRL2 regions of LL-1 and the CDRL3 and CDRL1 regions of GPC-A-1, respectively (Figure 4E). Interestingly, while 3D classification revealed classes of GPCs with GPC-A-1 alone, we were only able to observe the density for LL-1 in the presence of GPC-A-1, suggesting that the latter is required for LL-1 to engage the GPC. Thus, LL-1 falls under the previously described group of class II anti-immune complex Abs.[66] Unfortunately, sample limitations and the low mutability of the putative LL-1 epitope prohibited us from characterizing the neutralization potential of this response.[67]

Analysis of recombinant LASV GPC-induced Ab responses: The interior epitopes

Considering that GPCysR4-I53-50A trimers destabilize in the presence of pAbs (Figure 2A, right), it is conceivable that the same would happen *in vivo*. This would result in the trimer exposing its internal and non-neutralizing epitopes on GP1 and GP2. As they represent a considerable peptidic surface on a highly glycosylated protein, these internal sites would likely induce a dominant Ab response. To investigate if these interior epitopes were being targeted, we performed EMPEM experiments using a pool of week 30 pFabs from rabbits 187–192, which we incubated with monomeric GPC precomplexed with the 25.10C Fab (Figure 5A). This 25.10C Fab was used as a fiducial marker to aid in determining the orientation and alignment of the stalky, featureless monomer. 2D classifications of extracted single particles revealed classes with two equatorial Fabs binding opposite sides of the monomer. In most classes, an additional Fab targeted the top or bottom of the GPC monomer. Considering one of the two opposing equatorial Fabs includes 25.10C, the 2D classes suggest that interior-targeting responses were elicited in these rabbits.

We used single-particle cryo-EM to provide more detail on these presumed interior-targeting pFabs (Table S3). While the overall resolution of our map was limited due to the small size of the GPC monomer and the sample's vast heterogeneity, we were able to generate a map with adequate resolution to confidently dock in a GPC monomer bound to 25.10C (PDB: 7TYV [40]). As hypothesized, we observed two interior binding pFabs, which we designated Int-1 and Int-2, targeting surfaces that would be inaccessible in the context of trimeric GPCs (Figure 5B). Int-1 binds near the apex of the GPC monomer and makes clear contact with the α1 helix (aa 120–127), with contact likely extending to α2 (aa 130–144). Int-2 is presumably the equatorial response we observed by nsEMPEM and resides opposite the 25.10C epitope. It appears to predominantly engage the HR1d helix (aa 347–358) but likely also contacts the loop just downstream of HR1d (aa 359–362) and the beginning of the

(C) Serum neutralization of rabbits 187 and 189 to unmutated Josiah pseudovirus (wild type [WT]) and Josiah pseudovirus containing the E228K and D229Y mutations. Shown are the mean and standard deviation of three technical replicates.

(D) Atomic model of LL-1 pFab bound to GPCysRRLL-I53-50A as determined by cryo-EM, with GPC-A-1 in the background.

(A and D) Inset depicts presumed interactions between the modeled poly-alanine Fabs and the GPC. For (A), the inset is split up in a panel focusing on putative peptidic (left) and glycan (right) contacts. Residues depicted as sticks on the GPC are within 6 Å of the poly-alanine chain and, collectively, are considered the epitope footprint.

(E) Closeup of the map and model of LL-1 (red) and GPC-A-1 (blue), illustrating anti-idiotypic contacts between the two pFabs.

See also Figures S3, S4, and S6 and Table S3.

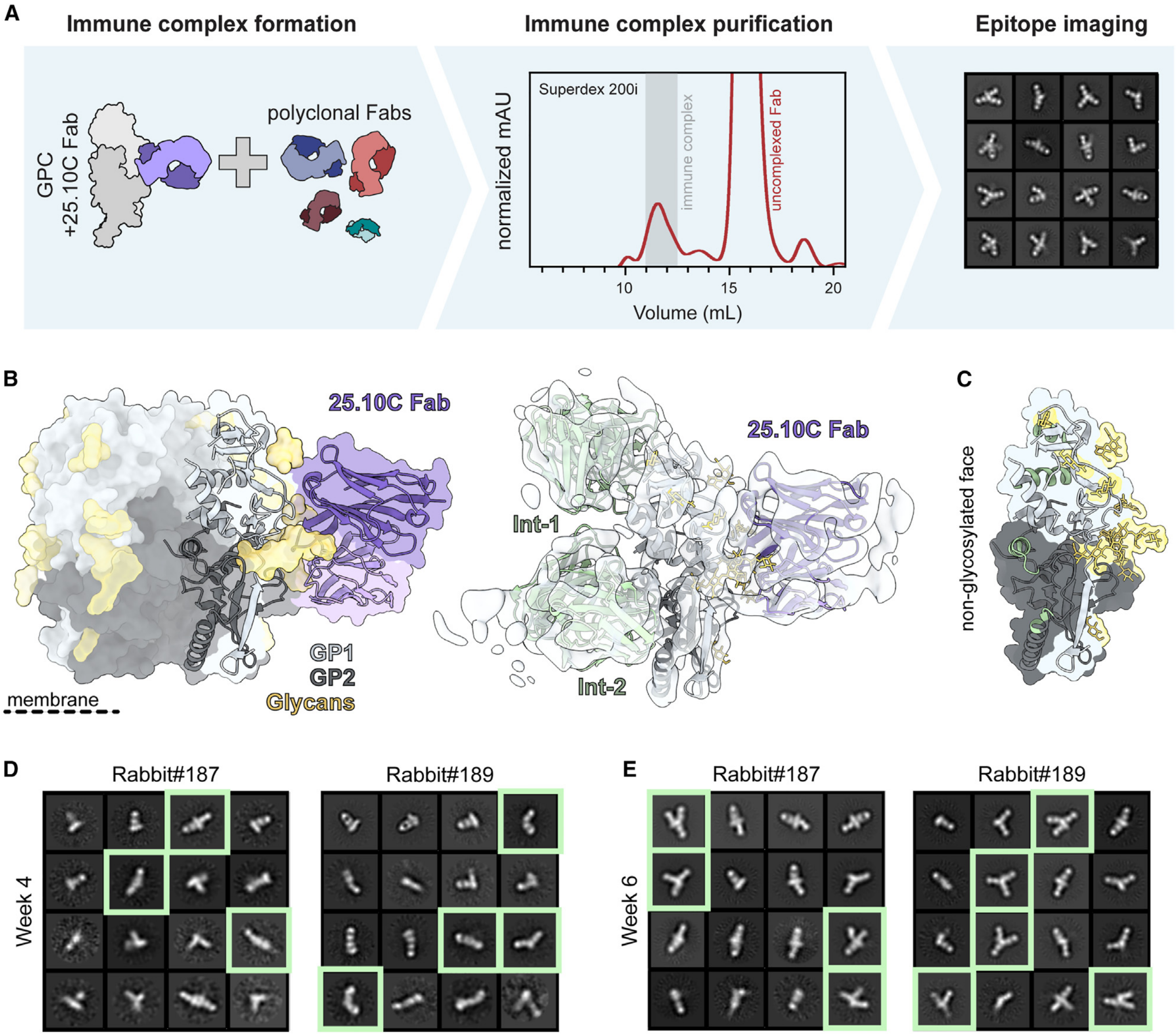

Figure 5. EMPEM experiments with GPC monomers reveals responses targeting the trimer interior

(A) Schematic of monomeric GPC EMPEM employing 25.10C Fab as a fiducial marker.

(B) Monomeric GPC cryo-EMPEM reveals the presence of interior binding pFabs Int-1 and Int-2. The GPC monomer with the 25.10C Fab (PDB: 7TYV[40]) is shown in a trimeric context (left). The cryo-EM density map enabled the docking of models of the GPC monomer (PDB: 7SGF[30]) with the 25.10C Fab (PDB: 7TYV[40]) and additional rabbit Fabs (PDB: 7RA7).

(C) Putative epitope footprints of Int-1 and Int-2 mapped onto GPC monomer show their preference for the interior, non-glycosylated GPC surface.

(D and E) 2D class averages of nsEMPEM experiments with monomeric GPC precomplexed with 25.10C Fab. Classes shown are the selected classes from two iterations of 2D classification that have been reclassified to 16 classes.

(D) 2D class averages of monomeric GPC nsEMPEM experiments with week 4 serum from rabbits 187 and 189. Classes with clear interior-binding pFabs are highlighted with green squares.

(E) 2D class averages of monomeric GPC nsEMPEM experiments with week 6 serum from rabbits 187 and 189. Classes with clear interior-binding pFabs are highlighted with green squares.

See also Table S3.

C-terminal helix HR2 (aa 398–402). Both pFabs show specificity for the non-glycosylated face of the exposed GPC interior surface (Figure 5C). Interestingly, the interior-binding pFabs targeted similar epitopes to those putatively described for non-NAbs elicited by healthy LASV-seropositive human survivors from Sierra Leone and Nigeria.[15] Int-1 shares its epitope with GP1-B Abs, which were originally thought to target residues between 119 and 134 while Int-2 engages with residues belonging to the presumptive GP2-B class of Abs.

Having confirmed the identity of these pAb responses by cryo-EM, we continued to perform nsEMPEM experiments with bleeds from earlier time points (Figures 5D and 5E). Complexes

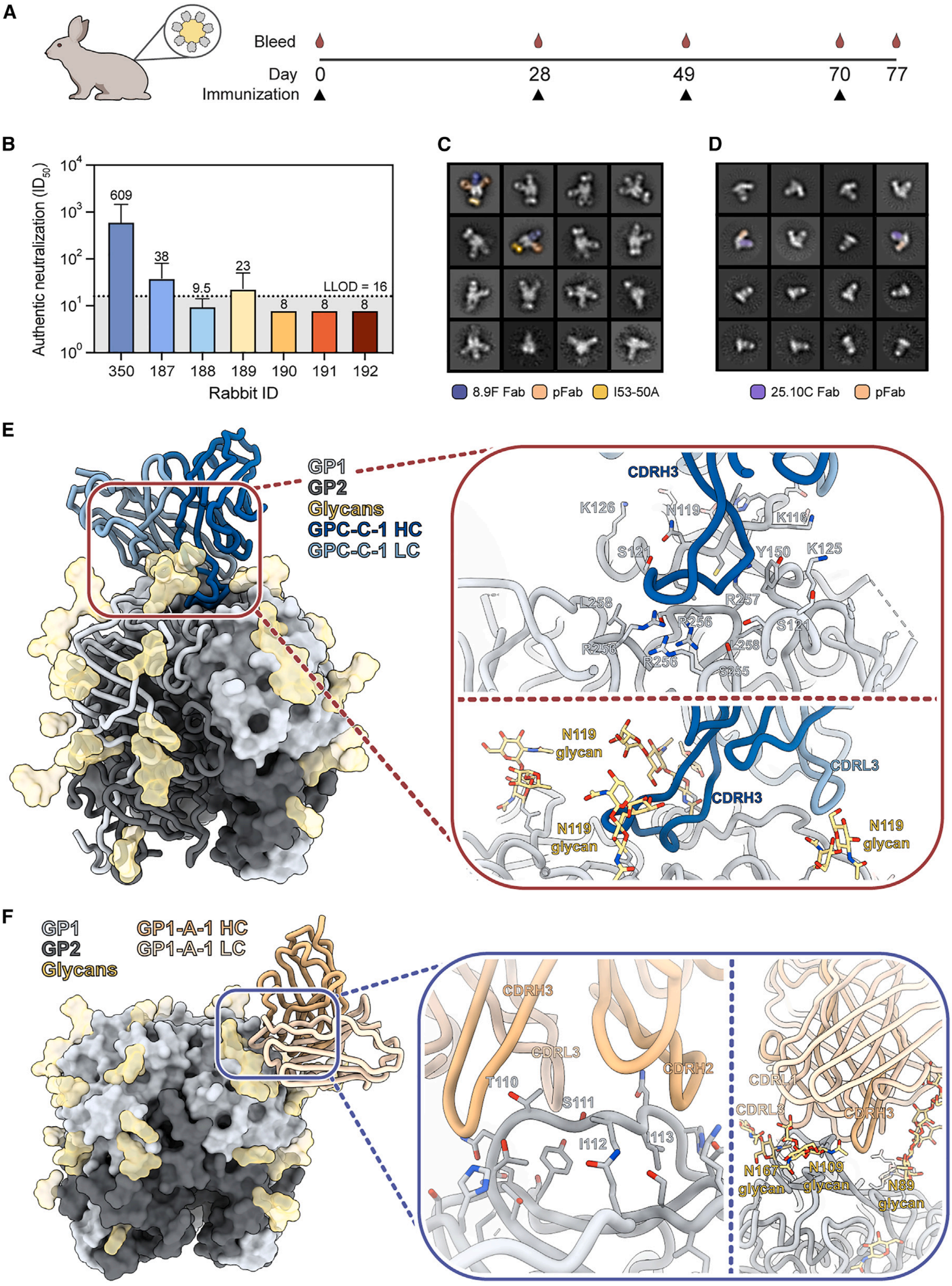

(legend on next page)

of the GPC monomer and 25.10C Fab were incubated with week 4 and 6 pFabs from rabbits 187 and 189 and imaged. At week 6, substantial Int-1 and Int-2 responses were observed in both rabbits, signified by the typical Y-shaped complexes in the 2D classes (Figure 5E). Even for the week 4 pFabs, for which we observed no responses to the trimeric GPC, complexes of Int-2 pFabs and monomeric GPC-25.10C Fabs were discernible among the 2D classes (Figure 5D). Together, these data suggest that interior-targeting responses dominate the immune response to GPCysR4-I53-50A trimers, showing up several immunizations before the elicitation of responses targeting neutralizing epitopes.

Analysis of Ab responses to a GPC-derived VLP

Following our investigation of the pAb responses to rabbits vaccinated with the recombinant GPC, we extended our EMPEM analysis to a potential vaccine candidate where the GPC is embedded in the membrane. A previously described GPC-derived VLP, generated by Madin-Darby canine kidney (MDCK) cells stably expressing a full-length GPC, elicited remarkably strong autologous neutralization titers after four immunizations in a two-rabbit animal study (Figure 6A).[53] Of note, these VLPs, which are generated by spontaneous budding of GPC-containing vesicles, present fully cleaved trimers only as opposed to the GPC expressed on the surface of infected or transfected cells, which presents both fully cleaved and uncleaved trimers (Figures S7A and S7B).[68] Our efforts focused on rabbit 350, which demonstrated the highest neutralization titers of the two and exhibited prophylactic and therapeutic *in vivo* efficacy in a lethal LASV infection model.[53,69] In a direct comparison with sera of GPCysR4-I53-50A-immunized rabbits, rabbit 350 induced an approximately 15-fold higher authentic neutralization titer than rabbit 187, the best neutralizer of the six rabbits (Figure 6B). In agreement with our findings that the base epitope is inaccessible on membrane-embedded GPC, we observed no base-targeting responses in nsEMPEM experiments with pFabs from rabbit 350 (Figure 6C). Rather, we observed what seemed to be several responses targeting the trimer apex (Figure S7C). Furthermore, nsEMPEM with the GPC monomer revealed a pAb response bound to the exterior of the GPC (likely one of the apex-targeting pFabs) but no clear interior-targeting pFabs (Figure 6D).

Given the absence of base-proximate responses, we performed cryo-EM using GPCysRRLL-I53-50A precomplexed with the 18.5C Fab. This led to the generation of two maps with a resolution of 2.8 Å each: one map highlighting a GPC-C response and the other a GP1-A response (Figures S3 and S4; Table S3). Upon closer inspection of the reconstructed model of the former, it became evident that the pFab, which we designate GPC-C-1, interacts with the GPC in a manner strikingly similar to the bNAb 8.9F (Figures 6E and S7D). Firstly, GPC-C-1 employs an unusually long CDRH3 (26 aa) to reach into the apical cavity of the trimer, establishing contact with all three protomers. Secondly, interactions with the glycan at position N119 were observed. Lastly, like 8.9F, the LC makes contact with only one of the three protomers, also engaging the N119 glycan. The high resolution of this map made it particularly suitable for automated atomic model building using ModelAngelo.[70] This machine-learning approach integrates data from the cryo-EM map and predicted amino acid probabilities to objectively build a structure with no prior sequence information. Consequently, ModelAngelo enabled informed speculation on the nature of certain GPC-pFab interactions where the pFab side chains were well resolved (Figure S7E). We note, for instance, a tyrosine at position 100K in the CDRH3, making contact with glycan N119. Additional aromatics can be observed at positions 100L in the CDRH3, 32 in the CDRL1, and 93 in the CDRL3, forming hydrophobic interactions with F117 in the GPC. Furthermore, ModelAngelo gave the highest probability for a tyrosine at position 100A in the CDRH3, which, considering the additional density, is presumably sulfated and makes electrostatic interactions with R256 and R257 in the GPC (Figure S7E). Based on our model, we suggest that GPC-C-1 played a substantial role in the observed neutralization in this rabbit by directly inhibiting the binding of the GPC to matriglycan.

The generated model of the GP1-A-like response revealed that the pFab, designated GP1-A-1, is positioned in between glycans N89, N109, 119, N167, and 224 and uses both its HC and LC to engage GP1. Besides several surrounding putative contacts with S91, D156, and K161, GP1-A-1 mainly targets the loop that extends over β5-β8 (aa 107–115) using its CDRH2, CDRH3, and CDRL3 (Figure 6F). Beyond these peptidic contacts, there seem to be extensive interactions between GP1-A-1 and glycans N89

Figure 6. GPC-derived VLP immunization in rabbits induces Ab responses to the GPC-C and GP1-A epitopes

(A) Immunization scheme of the rabbits immunized with a GPC-derived VLP showing a prime at day 0 and boosts at days 28, 49, and 70, as recently described.[53]

(B) Autologous neutralization titers to authentic LASV from GPCysR4-I53-50A-immunized rabbits (187–192) and a rabbit immunized with a GPC-derived VLP (rabbit 350). The virus neutralization titer is calculated as the geometric mean titer (GMT) of the reciprocal value of the last serum dilution at which inhibition of the cytopathic effect on infected Vero E6 cells is detectable. The initial dilution was 1:16 (lower limit of detection [LLOD]), so a titer of 8 was noted when no inhibition was observed. Shown is the geometric mean titer of four technical replicates with geometric standard deviation.

(C) 2D class averages of nsEMPEM experiments with pFabs from rabbit 350 using GPCysRRLL-I53-50A precomplexed with 8.9F. Classes shown are the selected classes from two iterations of 2D classification that have been reclassified to 16 classes. Two classes are pseudocolored according to the legend on the bottom to indicate the stabilizing Fab (8.9F Fab), pFab, and I53-50A.

(D) 2D class averages of monomeric GPC nsEMPEM experiments with pFab from rabbit 350. Classes shown are the selected classes from two iterations of 2D classification that have been reclassified to 16 classes. Two classes are pseudocolored according to the legend on the bottom to indicate the fiducial (25.10C Fab) and pFab.

(E) Atomic model of GPC-C-1 pFab bound to GPCysRRLL-I53-50A as determined by cryo-EM.

(F) Atomic model of GP1-A-1 pFab bound to GPCysRRLL-I53-50A as determined by cryo-EM.

(E and F) Inset depicts presumed interactions between the modeled poly-alanine pFab and the GPC. The inset is split up in a panel focusing on putative peptidic (top or left) and glycan (bottom or right) contacts. Residues depicted as sticks on the GPC are within 6 Å of the poly-alanine chain and, collectively, are considered the epitope footprint.

See also Figures S3, S4, and S7 and Table S3.

and, to a lesser extent, N109. ModelAngelo, for instance, predicted putative aromatics in CDRH3 making contact with glycan N89, as well as a lysine and phenylalanine in the CDRL1 and CDRL3, respectively, engaging with glycan N109 (Figure S7F). The way GP1-A-1 binds the GPC is remarkably similar to the previously characterized NAb 12.1F, and this is emphasized by the clear overlap in their epitope footprints (Figure S7G).[31] 12.1F also primarily engages residues between aa 107 and 115 and makes contact with glycans N89 and N109.[29,31] As a result, 12.1F neutralizes LASV by inhibiting the conformational change that GP1 undergoes at late endosomal pH (5.5) that is required for LAMP-1 binding. Thus, unlike GPC-C-1, GP1-A-1 would need to withstand a low-pH environment to confer neutralization, complicating speculations about its neutralizing potential based on the epitope alone.

DISCUSSION

The isolation of highly potent and protective NAbs from Lassa fever survivors demonstrates that the humoral immune response is capable of targeting sites of vulnerability on LASV's highly glycosylated GPC, providing promise for the development of LASV vaccines aimed at inducing NAb responses. Nevertheless, the various LASV vaccine efforts have failed to induce or only poorly induced NAb responses, and their elicited Ab responses are poorly understood. Detailed characterization of vaccine-induced Ab responses may be an important step in finding the bottlenecks for NAb development and designing more effective LASV vaccines. Serum ELISAs have been the standard to characterize vaccine-induced humoral responses, but they have generally been performed with non-stabilized, heterogeneous populations of GPCs. Furthermore, as we show here, even with prefusion-stabilized GPC trimers, pFab-induced disassembly of GPCs confounds interpretation. To address these shortcomings, we presented an EMPEM workflow for LASV that enables highly detailed structural characterization of responses to both trimeric and monomeric forms of prefusion GPCs. By testing our pipeline on sera from rabbit(s) previously vaccinated with GPC-I53-50A and a GPC-derived VLP, we identified several on- and off-target responses, including previously uncharacterized base and fusion peptide responses. These insights have implications for the development of next-generation recombinant GPC immunogens and may fuel the generation of vaccine candidates that have eliminated the exposure of undesired epitopes, paving the way for subdominant neutralizing responses to emerge.

EMPEM with sera from rabbits vaccinated with a recombinant GPC protein revealed that pAb responses consistently target a previously undescribed epitope that is located at the C-terminal helix and N-terminal loop. This base epitope is part of a larger surface at the bottom of the trimer that is close to the membrane and, consequentially, lacks glycans. EMPEM and neutralization assays with mAbs indicated that these base-targeting responses are non-neutralizing. We note a clear parallel to the base responses elicited by recombinant prefusion-stabilized HIV-1 envelope (Env) trimers like the prototypic BG505 SOSIP.664. Over the years, EMPEM studies have shown the consistency and immunodominance of base responses and guided the redesign of Env trimers that attempt to shield this irrelevant neoepitope.[54,58,62,71–74] The inability of base-targeting mAbs to bind membrane-embedded GPCs as well as the absence of base-targeting responses in the VLP-immunized rabbit suggest that the base epitope constitutes a neoepitope generated by truncation of the transmembrane domain on recombinant GPCs. This reveals a clear shortcoming of recombinant GPC vaccines. Still, future EMPEM studies on more VLP-immunized subjects, as well as those immunized with other vaccine modalities that use membrane-embedded GPCs (i.e., mRNA-, DNA-, or vector-based vaccines), will need to be performed to affirm if this neoepitope is merely a disadvantage of recombinant GPC vaccines. Nevertheless, informed by our details on the epitope-paratope interface, this highly immunogenic off-target epitope should be ameliorated in the next iteration of recombinant GPC immunogens. For example, introduction of glycans in the C-terminal helix or N-terminal loop could enhance shielding of this epitope. Alternatively, as trimeric GPC ectodomains currently require stabilization domains, adjusting the rigidity and length of the C-terminal linkers to their trimerization scaffolds may occlude accessibility of the base.

Another off-target response we observed exploits the interior of the GPC trimer. As this epitope would be inaccessible in the trimeric conformation of prefusion GPCysR4-I53-50A, these responses indicate that either the GPC is disassembling *in vivo* or the GPC is undergoing Ab-facilitated disassembly. This latter phenomenon has been observed extensively for influenza and HIV-1, where specific Ab responses have been shown to facilitate hemagglutinin and Env protein dissolution, respectively.[75–77] Our observations that interior-targeting responses were already elicited after the first immunization suggest that the GPCysR4-I53-50A readily disassembles *in vivo* before the emergence of other responses to the exterior of the trimer. Thus, Ab-facilitated disassembly does not seem to be the cause of interior-targeting responses. According to our analyses, this interior GPC surface contains epitopes that are the dominant target during early immunizations and likely compete with B cells in germinal centers that recognize functional epitopes. Our monomer EMPEM data also correspond to previous reports of non-NAbs targeting the GP1-B and GP2-B epitopes.[15] We offer structural definition to these Ab clusters with data that strongly suggest that these epitopes are present only on disassembled, non-trimeric GPCs. The fact that these mAbs were isolated from Lassa fever survivors suggests that natural infection presents GPC interiors, though the mechanism by which this happens is still unknown. Regardless, our results imply that fusing GPC to a trimerization domain is insufficient, and further stabilization of the trimeric interface should dictate the design of the next generation of recombinant GPC vaccines.

Our EMPEM work also revealed vaccine-induced pAb responses remarkably similar to previously characterized NAbs isolated from Lassa fever survivors, supporting the use of rabbits as vaccination models. GPCysR4-I53-50A induced a response in two out of six rabbits that correlated with neutralization and which we demonstrated parallels the canonical human GPC-A NAbs. Indeed, for rabbit 189, the GPC-A response was primarily responsible for its serum neutralization, presumably by inhibiting the pH-induced conformational changes in GP1 that are necessary for LAMP-1 engagement.[40] While the FP-1 response did not fully mirror the defined GPC-B epitope, it engages with some of

the same residues that are targeted by potent GPC-B NAbs.[27,28] Focusing heavily on the fusion loop and fusion peptide, we speculate that the FP-1 response may have the potential to mature toward neutralization, perhaps by increasing its affinity and/or making it resilient to acidic pH. Interestingly, the GPC-derived VLP, which induced markedly more potent neutralizing responses than GPCysR4-I53-50A, elicited very different on-target responses. This vaccine induced responses very similar to the GPC-C and GP1-A NAbs 8.9F and 12.1F, respectively, which are two of the three NAbs that make up Arevirumab-3.[29,43] The prediction of a sulfated tyrosine in GPC-C-1, as well as the presence of multiple sulfated tyrosines in 8.9F, hints at a common strategy to target the positively charged apex of GPCs. This is analogous by apex-targeting bNAbs to HIV-1, which utilize sulfated tyrosines in their extended CDRH3s to interact with the positively charged V2 loop.[78–80] The engagement of GPC-C-1 with the native-cleavage site reinforces earlier suggestions that these residues are necessary for the elicitation of apex-targeting Abs and provides one explanation for the differences in NAb responses between the two vaccine modalities in this study.[29] Another explanation may be differences in epitope presentation, such as the valency of the immunogen display and accessibility of the epitopes. It is tempting to perceive the remarkable neutralization titer rabbit 350 generated in the context of the VLP's inability to induce off-target responses to the base and interior. It should be considered, however, that also this immunogen required an extensive prime-boost regimen to achieve its potent neutralization. Further studies are required to assess if this is merely attributable to the overall low immunogenicity of GPCs or if other factors play a role. Nevertheless, integrating other vaccine strategies such as epitope focusing or germline targeting with this VLP platform may prove essential to overcome the low immunogenicity of this densely glycosylated protein.[81]

In conclusion, we have presented direct, high-resolution visualization of vaccine-induced humoral immune responses to a prefusion GPC in its trimeric and monomeric conformational states. By identifying on- and off-target responses, we have highlighted sites of vulnerability on this densely glycosylated immunogen and revealed potential bottlenecks to the elicitation of NAb responses. Our demonstration of the immunodominance of the trimer base and interior not only has strong implications for the vaccine design of recombinant GPC immunogens but also hints at the advantage of vaccines that present GPCs on a membrane. As more attention is drawn to combatting Lassa fever, and as several vaccine candidates are moving to the clinic, the careful and robust assessment of humoral responses may be more important than ever. The work described here supports the use of our EMPEM workflow as an integral component of serological assays that may considerably advance the understanding of induced Ab responses and, concomitantly, the development of a protective Lassa vaccine.

Limitations of the study

Although EMPEM offers a rapid and rather comprehensive visual analysis of the humoral response, working at the polyclonal level comes with obvious limitations. As opposed to mAbs, the polyclonality of the Ab sample makes identifying the affinity of a certain response or its neutralization potential inherently complex. Inferring the latter based on merely the overlap with a known NAb epitope is further complicated by the requirement that the pAb remains associated at acidic pH to interfere with viral fusion in the endosome (the pAbs that target the GPC-C epitope and block matriglycan binding being the exception). Although we have been successful in showing the role GPC-A-2 plays in serum neutralization of rabbit 189 with a pseudovirus mutant, we were unable to do so for the responses in rabbits 187 and 350. Sample limitations prohibited the exhaustive search for epitope knockouts. Beyond the limitations that the polyclonality of the Abs provide, we note several technical limitations to the EMPEM pipeline. As we demonstrated with LL-1, nsEMPEM may not be adequate for identifying low-abundance pAb responses or distinguishing those residing in close proximity to one another. Orientation bias and alignment of the featureless GPC remains a challenge for 3D reconstructions of the featureless GPC, although the latter is dramatically improved with fiducials like the 8.9F Fab. Thus, while nsEMPEM provides a good baseline for capturing the broad strokes of pAb responses, the more time- and resource-intensive—yet higher-resolution—cryo-EMPEM provides detail and distinguishes responses residing in close proximity. Still, for cryo-EMPEM, issues such as orientation bias, freeze-induced denaturation, and abundance of binding pFabs may confound observations. It is therefore key to note that we cannot exclude the presence of responses other than the ones described herein. Finally, we cannot exclude that both 8.9F and 18.5C Fabs may cause allosteric effects that could preclude the binding of some pFabs. Despite these caveats, nsEMPEM and cryo-EMPEM are unique methods that provide visual and structural information on the dominant Ab specificities in polyclonal sera against GPCs.

RESOURCE AVAILABILITY

Lead contact

Further information and requests for resources and reagents should be directed to and will be fulfilled by the lead contact, Andrew B. Ward (andrew@scripps.edu).

Materials availability

All reagents will be made available on request after completion of a materials transfer agreement.

Data and code availability

- Maps generated from the cryo-EM data are deposited in the Electron Microscopy Databank (http://www.emdatabank.org/) under the accession IDs EMD-41713, EMD-41715, EMD-41716, EMD-43141, EMD-43168, EMD-45624, EMD-45625, EMD-45643, EMD-45644, and EMD-45905. Maps generated from the nsEM data are deposited in the Electron Microscopy Databank (http://www.emdatabank.org/) under the accession IDs EMD-43174, EMD-43175, EMD-43176, EMD-43177, EMD-43178, EMD-43179, EMD-43180, EMD-43181, EMD-43182, and EMD-43183. Atomic models corresponding to these maps have been deposited in the Protein Data Bank (http://www.rcsb.org/) under PDB: 9CJ7, 9CJ8, 9CK7, 9CK8, 8TYC, 8TYE, 8VCV, and 8VE8. See Tables S2 and S3 for information on the deposited maps and models. The raw data reported in this study will be shared by the corresponding author upon request.
- This paper does not report original code.
- Any additional information required to reanalyze the data reported in this work paper is available from the lead contact upon request.

CellPress
OPEN ACCESS

ACKNOWLEDGMENTS

The authors thank Bill Anderson, William Lessin, and Hannah Turner from The Scripps Research Institute for their help with EM experiments and Gabriel Ozorowski for help with cryo-EM data processing. We thank Gotthard Ludwig and Sebastian Schmidt from the biosafety level 4 facility at the Philipps-University of Marburg for technical support. We thank Robin Shattock for kindly sharing the full-length native Josiah GPC plasmid. H.R.P. is supported by a David C. Fairchild Endowed Fellowship, Achievement Rewards for College Scientists Foundation, and NIH F31 Ruth L. Kirschstein Predoctoral Award 1F31AI172358. P.J.M.B. is supported by Rubicon fellowship #452020226 from the Netherlands Organisation for Scientific Research. Further support from the Vici fellowship from the Netherlands Organisation for Scientific Research (NWO; to R.W.S.); the Fondation Dormeur, Vaduz (to R.W.S. and M.J.v.G.); the Deutsche Forschungsgemeinschaft-Projektnummer 197785619/SFB1021 (to T.S.); NIH grant R01 AI171438 (to A.B.W.); and the Bill and Melinda Gates Foundation through grant OPP1170236 (to A.B.W.) enabled this work. Biorender.com was used to make the graphical abstract and elements in Figures 2 and 6.

AUTHOR CONTRIBUTIONS

Conceptualization, P.J.M.B., H.R.P., and A.B.W.; methodology, P.J.M.B., H.R.P., T.B., J.A.F., and H.M.-K.; formal analysis, P.J.M.B., H.R.P., and T.B.; investigation, P.J.M.B., H.R.P., T.B., H.N., S.K., J.A.B., I.B., W.-H.L., M.S., and H.M.-K.; resources, R.W.S., T.S., M.J.v.G., and A.B.W.; writing—original draft, P.J.M.B., H.R.P., and A.B.W.; reviewing, editing, and other feedback, P.J.M.B., H.R.P., T.B., J.A.B., R.W.S., T.S., M.J.v.G., and A.B.W.; visualization, P.J.M.B. and H.R.P.; supervision, T.B., R.W.S., M.J.v.G., and A.B.W.; project administration, P.J.M.B., H.R.P., and A.B.W.; funding acquisition, R.W.S., T.S., M.J.v.G., and A.B.W.

DECLARATION OF INTERESTS

The authors declare no competing interests.

STAR★METHODS

Detailed methods are provided in the online version of this paper and include the following:

- KEY RESOURCES TABLE
- EXPERIMENTAL MODEL AND STUDY PARTICIPANT DETAILS
  - Cell lines
- METHOD DETAILS
  - Construct design
  - Protein expression and purification
  - Isolation of GPC-specific B cells
  - Antibody cloning
  - Antibody binding to membrane expressed GPC
  - Antibody binding by biolayer interferometry
  - SDS-PAGE analysis
  - Differential scanning fluorimetry
  - Fab preparation for EMPEM studies
  - Preparation of GPC immune complexes with polyclonal rabbit Fabs
  - Preparation of GPC immune complexes with LAVA05 and LAVA06
  - Negative stain electron microscopy
  - Negative stain electron microscopy data processing
  - Generation of LASV pseudovirus
  - Pseudovirus neutralization assay
  - Authentic neutralization assay
  - Cryo-EM grid preparation and data collection
  - Cryo-EM data processing
  - Atomic model refinement
  - ModelAngelo

SUPPLEMENTAL INFORMATION

Supplemental information can be found online at https://doi.org/10.1016/j.celrep.2024.114708.

Received: January 3, 2024
Revised: July 19, 2024
Accepted: August 19, 2024
Published: September 6, 2024

REFERENCES

1. Monath, T.P., Newhouse, V.F., Kemp, G.E., Setzer, H.W., and Cacciapuoti, A. (1974). Lassa virus isolation from Mastomys natalensis rodents during an epidemic in Sierra Leone. Science *185*, 263–265.
2. Dan-Nwafor, C.C., Ipadeola, O., Smout, E., Ilori, E., Adeyemo, A., Umeokonkwo, C., Nwidi, D., Nwachukwu, W., Ukponu, W., Omabe, E., et al. (2019). A cluster of nosocomial Lassa fever cases in a tertiary health facility in Nigeria: Description and lessons learned, 2018. Int. J. Infect. Dis. *83*, 88–94.
3. Raabe, V., and Koehler, J. (2017). Laboratory Diagnosis of Lassa Fever. J. Clin. Microbiol. *55*, 1629–1637.
4. Thielebein, A., Ighodalo, Y., Taju, A., Olokor, T., Omiunu, R., Esumeh, R., Ebhodaghe, P., Ekanem, A., Igenegbale, G., Giwa, R., et al. (2022). Virus persistence after recovery from acute Lassa fever in Nigeria: a 2-year interim analysis of a prospective longitudinal cohort study. Lancet. Microbe *3*, e32–e40.
5. Garry, R.F. (2023). Lassa fever - the road ahead. Nat. Rev. Microbiol. *21*, 87–96.
6. Basinski, A.J., Fichet-Calvet, E., Sjodin, A.R., Varrelman, T.J., Remien, C.H., Layman, N.C., Bird, B.H., Wolking, D.J., Monagin, C., Ghersi, B.M., et al. (2021). Bridging the gap: Using reservoir ecology and human serosurveys to estimate Lassa virus spillover in West Africa. PLoS Comput. Biol. *17*, e1008811.
7. McCormick, J.B., King, I.J., Webb, P.A., Johnson, K.M., O'Sullivan, R., Smith, E.S., Trippel, S., and Tong, T.C. (1987). A case-control study of the clinical diagnosis and course of Lassa fever. J. Infect. Dis. *155*, 445–455.
8. Cummins, D., McCormick, J.B., Bennett, D., Samba, J.A., Farrar, B., Machin, S.J., and Fisher-Hoch, S.P. (1990). Acute sensorineural deafness in Lassa fever. JAMA *264*, 2093–2096.
9. Liao, B.S., Byl, F.M., and Adour, K.K. (1992). Audiometric comparison of Lassa fever hearing loss and idiopathic sudden hearing loss: evidence for viral cause. Otolaryngol. Head Neck Surg. *106*, 226–229.
10. Mateer, E.J., Huang, C., Shehu, N.Y., and Paessler, S. (2018). Lassa fever-induced sensorineural hearing loss: A neglected public health and social burden. PLoS Neglected Trop. Dis. *12*, e0006187.
11. Kayem, N.D., Benson, C., Aye, C.Y.L., Barker, S., Tome, M., Kennedy, S., Ariana, P., and Horby, P. (2020). Lassa fever in pregnancy: a systematic review and meta-analysis. Trans. R. Soc. Trop. Med. Hyg. *114*, 385–396.
12. Mehand, M.S., Al-Shorbaji, F., Millett, P., and Murgue, B. (2018). The WHO R&D Blueprint: 2018 review of emerging infectious diseases requiring urgent research and development efforts. Antivir. Res. *159*, 63–67.
13. LaVergne, S.M., Sakabe, S., Momoh, M., Kanneh, L., Bond, N., Garry, R.F., Grant, D.S., de la Torre, J.C., Oldstone, M.B.A., Schieffelin, J.S., and Sullivan, B.M. (2022). Expansion of CD8+ T cell population in Lassa virus survivors with low T cell precursor frequency reveals durable immune response in most survivors. PLoS Neglected Trop. Dis. *16*, e0010882.
14. Botten, J., Alexander, J., Pasquetto, V., Sidney, J., Barrowman, P., Ting, J., Peters, B., Southwood, S., Stewart, B., Rodriguez-Carreno, M.P., et al. (2006). Identification of protective Lassa virus epitopes that are restricted by HLA-A2. J. Virol. *80*, 8351–8361.
15. Robinson, J.E., Hastie, K.M., Cross, R.W., Yenni, R.E., Elliott, D.H., Rouelle, J.A., Kannadka, C.B., Smira, A.A., Garry, C.E., Bradley, B.T., et al. (2016).

Most neutralizing human monoclonal antibodies target novel epitopes requiring both Lassa virus glycoprotein subunits. Nat. Commun. *7*, 11544.

16. Katz, M., Weinstein, J., Eilon-Ashkenazy, M., Gehring, K., Cohen-Dvashi, H., Elad, N., Fleishman, S.J., and Diskin, R. (2022). Structure and receptor recognition by the Lassa virus spike complex. Nature *603*, 174–179.
17. Eichler, R., Lenz, O., Strecker, T., and Garten, W. (2003). Signal peptide of Lassa virus glycoprotein GP-C exhibits an unusual length. FEBS Lett. *538*, 203–206.
18. Bederka, L.H., Bonhomme, C.J., Ling, E.L., and Buchmeier, M.J. (2014). Arenavirus stable signal peptide is the keystone subunit for glycoprotein complex organization. mBio *5*, e02063.
19. Rojek, J.M., Lee, A.M., Nguyen, N., Spiropoulou, C.F., and Kunz, S. (2008). Site 1 protease is required for proteolytic processing of the glycoproteins of the South American hemorrhagic fever viruses Junin, Machupo, and Guanarito. J. Virol. *82*, 6045–6051.
20. Cao, W., Henry, M.D., Borrow, P., Yamada, H., Elder, J.H., Ravkov, E.V., Nichol, S.T., Compans, R.W., Campbell, K.P., and Oldstone, M.B. (1998). Identification of alpha-dystroglycan as a receptor for lymphocytic choriomeningitis virus and Lassa fever virus. Science *282*, 2079–2081.
21. Kunz, S., Rojek, J.M., Perez, M., Spiropoulou, C.F., and Oldstone, M.B.A. (2005). Characterization of the interaction of lassa fever virus with its cellular receptor alpha-dystroglycan. J. Virol. *79*, 5979–5987.
22. Sheikh, M.O., Capicciotti, C.J., Liu, L., Praissman, J., Ding, D., Mead, D.G., Brindley, M.A., Willer, T., Campbell, K.P., Moremen, K.W., et al. (2022). Cell surface glycan engineering reveals that matriglycan alone can recapitulate dystroglycan binding and function. Nat. Commun. *13*, 3617.
23. Oppliger, J., Torriani, G., Herrador, A., and Kunz, S. (2016). Lassa Virus Cell Entry via Dystroglycan Involves an Unusual Pathway of Macropinocytosis. J. Virol. *90*, 6412–6429.
24. Israeli, H., Cohen-Dvashi, H., Shulman, A., Shimon, A., and Diskin, R. (2017). Mapping of the Lassa virus LAMP1 binding site reveals unique determinants not shared by other old world arenaviruses. PLoS Pathog. *13*, e1006337.
25. Jae, L.T., Raaben, M., Herbert, A.S., Kuehne, A.I., Wirchnianski, A.S., Soh, T.K., Stubbs, S.H., Janssen, H., Damme, M., Saftig, P., et al. (2014). Virus entry. Lassa virus entry requires a trigger-induced receptor switch. Science *344*, 1506–1510.
26. Shulman, A., Katz, M., Cohen-Dvashi, H., Greenblatt, H.M., Levy, Y., and Diskin, R. (2019). Variations in Core Packing of GP2 from Old World Mammarenaviruses in their Post-Fusion Conformations Affect Membrane-Fusion Efficiencies. J. Mol. Biol. *431*, 2095–2111.
27. Hastie, K.M., Zandonatti, M.A., Kleinfelter, L.M., Heinrich, M.L., Rowland, M.M., Chandran, K., Branco, L.M., Robinson, J.E., Garry, R.F., and Saphire, E.O. (2017). Structural basis for antibody-mediated neutralization of Lassa virus. Science *356*, 923–928.
28. Hastie, K.M., Cross, R.W., Harkins, S.S., Zandonatti, M.A., Koval, A.P., Heinrich, M.L., Rowland, M.M., Robinson, J.E., Geisbert, T.W., Garry, R.F., et al. (2019). Convergent Structures Illuminate Features for Germline Antibody Binding and Pan-Lassa Virus Neutralization. Cell *178*, 1004–1015.e14.
29. Li, H., Buck, T., Zandonatti, M., Yin, J., Moon-Walker, A., Fang, J., Koval, A., Heinrich, M.L., Rowland, M.M., Diaz Avalos, R., et al. (2022). A cocktail of protective antibodies subverts the dense glycan shield of Lassa virus. Sci. Transl. Med. *14*, eabq0991.
30. Brouwer, P.J.M., Antanasijevic, A., Ronk, A.J., Müller-Kräuter, H., Watanabe, Y., Claireaux, M., Perrett, H.R., Bijl, T.P.L., Grobben, M., Umotoy, J.C., et al. (2022). Lassa virus glycoprotein nanoparticles elicit neutralizing antibody responses and protection. Cell Host Microbe *30*, 1759–1772.e12.
31. Perrett, H.R., Brouwer, P.J.M., Hurtado, J., Newby, M.L., Liu, L., Müller-Kräuter, H., Müller Aguirre, S., Burger, J.A., Bouhuijs, J.H., Gibson, G., et al. (2023). Structural conservation of Lassa virus glycoproteins and recognition by neutralizing antibodies. Cell Rep. *42*, 112524.
32. Flatz, L., Rieger, T., Merkler, D., Bergthaler, A., Regen, T., Schedensack, M., Bestmann, L., Verschoor, A., Kreutzfeldt, M., Brück, W., et al. (2010). T cell-dependence of Lassa fever pathogenesis. PLoS Pathog. *6*, e1000836.
33. Port, J.R., Wozniak, D.M., Oestereich, L., Pallasch, E., Becker-Ziaja, B., Müller, J., Rottstegge, M., Olal, C., Gómez-Medina, S., Oyakhliome, J., et al. (2020). Severe Human Lassa Fever Is Characterized by Nonspecific T-Cell Activation and Lymphocyte Homing to Inflamed Tissues. J. Virol. *94*, e01367-20. https://doi.org/10.1128/JVI.01367-20.
34. McCormick, J.B., and Fisher-Hoch, S.P. (2002). Lassa fever. Curr. Top. Microbiol. Immunol. *262*, 75–109.
35. Sommerstein, R., Flatz, L., Remy, M.M., Malinge, P., Magistrelli, G., Fischer, N., Sahin, M., Bergthaler, A., Igonet, S., Ter Meulen, J., et al. (2015). Arenavirus Glycan Shield Promotes Neutralizing Antibody Evasion and Protracted Infection. PLoS Pathog. *11*, e1005276.
36. Watanabe, Y., Raghwani, J., Allen, J.D., Seabright, G.E., Li, S., Moser, F., Huiskonen, J.T., Strecker, T., Bowden, T.A., and Crispin, M. (2018). Structure of the Lassa virus glycan shield provides a model for immunological resistance. Proc. Natl. Acad. Sci. USA *115*, 7320–7325.
37. Hastie, K.M., and Saphire, E.O. (2018). Lassa virus glycoprotein: stopping a moving target. Curr. Opin. Virol. *31*, 52–58.
38. Lenz, O., ter Meulen, J., Klenk, H.D., Seidah, N.G., and Garten, W. (2001). The Lassa virus glycoprotein precursor GP-C is proteolytically processed by subtilase SKI-1/S1P. Proc. Natl. Acad. Sci. USA *98*, 12701–12705.
39. Branco, L.M., Grove, J.N., Boisen, M.L., Shaffer, J.G., Goba, A., Fullah, M., Momoh, M., Grant, D.S., and Garry, R.F. (2011). Emerging trends in Lassa fever: redefining the role of immunoglobulin M and inflammation in diagnosing acute infection. Virol. J. *8*, 478.
40. Enriquez, A.S., Buck, T.K., Li, H., Norris, M.J., Moon-Walker, A., Zandonatti, M.A., Harkins, S.S., Robinson, J.E., Branco, L.M., Garry, R.F., et al. (2022). Delineating the mechanism of anti-Lassa virus GPC-A neutralizing antibodies. Cell Rep. *39*, 110841.
41. Buck, T.K., Enriquez, A.S., Schendel, S.L., Zandonatti, M.A., Harkins, S.S., Li, H., Moon-Walker, A., Robinson, J.E., Branco, L.M., Garry, R.F., et al. (2022). Neutralizing Antibodies against Lassa Virus Lineage I. mBio *13*, e0127822.
42. Cross, R.W., Mire, C.E., Branco, L.M., Geisbert, J.B., Rowland, M.M., Heinrich, M.L., Goba, A., Momoh, M., Grant, D.S., Fullah, M., et al. (2016). Treatment of Lassa virus infection in outbred guinea pigs with first-in-class human monoclonal antibodies. Antivir. Res. *133*, 218–222.
43. Mire, C.E., Cross, R.W., Geisbert, J.B., Borisevich, V., Agans, K.N., Deer, D.J., Heinrich, M.L., Rowland, M.M., Goba, A., Momoh, M., et al. (2017). Human-monoclonal-antibody therapy protects nonhuman primates against advanced Lassa fever. Nat. Med. *23*, 1146–1149.
44. Cashman, K.A., Wilkinson, E.R., Shaia, C.I., Facemire, P.R., Bell, T.M., Bearss, J.J., Shamblin, J.D., Wollen, S.E., Broderick, K.E., Sardesai, N.Y., and Schmaljohn, C.S. (2017). A DNA vaccine delivered by dermal electroporation fully protects cynomolgus macaques against Lassa fever. Hum. Vaccines Immunother. *13*, 2902–2911.
45. Abreu-Mota, T., Hagen, K.R., Cooper, K., Jahrling, P.B., Tan, G., Wirblich, C., Johnson, R.F., and Schnell, M.J. (2018). Non-neutralizing antibodies elicited by recombinant Lassa-Rabies vaccine are critical for protection against Lassa fever. Nat. Commun. *9*, 4223.
46. Cross, R.W., Xu, R., Matassov, D., Hamm, S., Latham, T.E., Gerardi, C.S., Nowak, R.M., Geisbert, J.B., Ota-Setlik, A., Agans, K.N., et al. (2020). Quadrivalent VesiculoVax vaccine protects nonhuman primates from viral-induced hemorrhagic fever and death. J. Clin. Invest. *130*, 539–551.
47. Fischer, R.J., Purushotham, J.N., van Doremalen, N., Sebastian, S., Meade-White, K., Cordova, K., Letko, M., Jeremiah Matson, M., Feldmann, F., Haddock, E., et al. (2021). ChAdOx1-vectored Lassa fever vaccine elicits a robust cellular and humoral immune response and protects guinea pigs against lethal Lassa virus challenge. NPJ Vaccines *6*, 32.
48. Mateo, M., Reynard, S., Journeaux, A., Germain, C., Hortion, J., Carnec, X., Picard, C., Baillet, N., Borges-Cardoso, V., Merabet, O., et al. (2021).

CellPress
OPEN ACCESS

A single-shot Lassa vaccine induces long-term immunity and protects cynomolgus monkeys against heterologous strains. Sci. Transl. Med. *13*, eabf6348. https://doi.org/10.1126/scitranslmed.abf6348.

49. Stein, D.R., Warner, B.M., Soule, G., Tierney, K., Frost, K.L., Booth, S., and Safronetz, D. (2019). A recombinant vesicular stomatitis-based Lassa fever vaccine elicits rapid and long-term protection from lethal Lassa virus infection in guinea pigs. NPJ Vaccines *4*, 8.

50. Wang, M., Li, R., Li, Y., Yu, C., Chi, X., Wu, S., Liu, S., Xu, J., and Chen, W. (2021). Construction and Immunological Evaluation of an Adenoviral Vector-Based Vaccine Candidate for Lassa Fever. Viruses *13*, 484. https://doi.org/10.3390/v13030484.

51. Gorman, J., Cheung, C.S.-F., Duan, Z., Ou, L., Wang, M., Chen, X., Cheng, C., Biju, A., Sun, Y., Wang, P., et al. (2024). Cleavage-intermediate Lassa virus trimer elicits neutralizing responses, identifies neutralizing nanobodies, and reveals an apex-situated site-of-vulnerability. Nat. Commun. *15*, 285.

52. Branco, L.M., Grove, J.N., Geske, F.J., Boisen, M.L., Muncy, I.J., Magliato, S.A., Henderson, L.A., Schoepp, R.J., Cashman, K.A., Hensley, L.E., and Garry, R.F. (2010). Lassa virus-like particles displaying all major immunological determinants as a vaccine candidate for Lassa hemorrhagic fever. Virol. J. *7*, 279.

53. Müller, H., Fehling, S.K., Dorna, J., Urbanowicz, R.A., Oestereich, L., Krebs, Y., Kolesnikova, L., Schauflinger, M., Krähling, V., Magassouba, N. 'faly, et al. (2020). Adjuvant formulated virus-like particles expressing native-like forms of the Lassa virus envelope surface glycoprotein are immunogenic and induce antibodies with broadly neutralizing activity. NPJ Vaccines *5*, 71.

54. Bianchi, M., Turner, H.L., Nogal, B., Cottrell, C.A., Oyen, D., Pauthner, M., Bastidas, R., Nedellec, R., McCoy, L.E., Wilson, I.A., et al. (2018). Electron-Microscopy-Based Epitope Mapping Defines Specificities of Polyclonal Antibodies Elicited during HIV-1 BG505 Envelope Trimer Immunization. Immunity *49*, 288–300.e8.

55. Antanasijevic, A., Sewall, L.M., Cottrell, C.A., Carnathan, D.G., Jimenez, L.E., Ngo, J.T., Silverman, J.B., Groschel, B., Georgeson, E., Bhiman, J., et al. (2021). Polyclonal antibody responses to HIV Env immunogens resolved using cryoEM. Nat. Commun. *12*, 4817.

56. Bangaru, S., Antanasijevic, A., Kose, N., Sewall, L.M., Jackson, A.M., Suryadevara, N., Zhan, X., Torres, J.L., Copps, J., de la Peña, A.T., et al. (2022). Structural mapping of antibody landscapes to human betacoronavirus spike proteins. Sci. Adv. *8*, eabn2911.

57. Han, J., Schmitz, A.J., Richey, S.T., Dai, Y.-N., Turner, H.L., Mohammed, B.M., Fremont, D.H., Ellebedy, A.H., and Ward, A.B. (2021). Polyclonal epitope mapping reveals temporal dynamics and diversity of human antibody responses to H5N1 vaccination. Cell Rep. *34*, 108682.

58. Nogal, B., Bianchi, M., Cottrell, C.A., Kirchdoerfer, R.N., Sewall, L.M., Turner, H.L., Zhao, F., Sok, D., Burton, D.R., Hangartner, L., and Ward, A.B. (2020). Mapping Polyclonal Antibody Responses in Non-human Primates Vaccinated with HIV Env Trimer Subunit Vaccines. Cell Rep. *30*, 3755–3765.e7.

59. Monigatti, F., Gasteiger, E., Bairoch, A., and Jung, E. (2002). The Sulfinator: predicting tyrosine sulfation sites in protein sequences. Bioinformatics *18*, 769–770.

60. Cottrell, C.A., van Schooten, J., Bowman, C.A., Yuan, M., Oyen, D., Shin, M., Morpurgo, R., van der Woude, P., van Breemen, M., Torres, J.L., et al. (2020). Mapping the immunogenic landscape of near-native HIV-1 envelope trimers in non-human primates. PLoS Pathog. *16*, e1008753.

61. van Schooten, J., van Haaren, M.M., Li, H., McCoy, L.E., Havenar-Daughton, C., Cottrell, C.A., Burger, J.A., van der Woude, P., Helgers, L.C., Tomris, I., et al. (2021). Antibody responses induced by SHIV infection are more focused than those induced by soluble native HIV-1 envelope trimers in non-human primates. PLoS Pathog. *17*, e1009736.

62. Cirelli, K.M., Carnathan, D.G., Nogal, B., Martin, J.T., Rodriguez, O.L., Upadhyay, A.A., Enemuo, C.A., Gebru, E.H., Choe, Y., Viviano, F., et al. (2020). Slow Delivery Immunization Enhances HIV Neutralizing Antibody and Germinal Center Responses via Modulation of Immunodominance. Cell *180*, 206.

63. Dingens, A.S., Pratap, P., Malone, K., Hilton, S.K., Ketas, T., Cottrell, C.A., Overbaugh, J., Moore, J.P., Klasse, P.J., Ward, A.B., and Bloom, J.D. (2021). High-resolution mapping of the neutralizing and binding specificities of polyclonal sera post-HIV Env trimer vaccination. Elife *10*, e64281. https://doi.org/10.7554/eLife.64281.

64. Guthmiller, J.J., Han, J., Utset, H.A., Li, L., Lan, L.Y.-L., Henry, C., Stamper, C.T., McMahon, M., O'Dell, G., Fernández-Quintero, M.L., et al. (2022). Broadly neutralizing antibodies target a haemagglutinin anchor epitope. Nature *602*, 314–320.

65. Pan, R., Chen, Y., Vaine, M., Hu, G., Wang, S., Lu, S., and Kong, X.-P. (2015). Structural analysis of a novel rabbit monoclonal antibody R53 targeting an epitope in HIV-1 gp120 C4 region critical for receptor and coreceptor binding. Emerg. Microb. Infect. *4*, e44.

66. Brown, S., Antanasijevic, A., Sewall, L.M., Garcia, D.M., Brouwer, P.J.M., Sanders, R.W., and Ward, A.B. (2024). Anti-Immune Complex Antibodies are Elicited During Repeated Immunization with HIV Env Immunogens. Preprint at bioRxiv. https://doi.org/10.1101/2024.03.15.585257.

67. Carr, C.R., Crawford, K.H.D., Murphy, M., Galloway, J.G., Haddox, H.K., Matsen, F.A., 4th, Andersen, K.G., King, N.P., and Bloom, J.D. (2024). Deep mutational scanning reveals functional constraints and antigenic variability of Lassa virus glycoprotein complex. Preprint at bioRxiv. https://doi.org/10.1101/2024.02.05.579020.

68. Zhu, X., Liu, Y., Guo, J., Cao, J., Wang, Z., Xiao, G., and Wang, W. (2021). Effects of N-Linked Glycan on Lassa Virus Envelope Glycoprotein Cleavage, Infectivity, and Immune Response. Virol. Sin. *36*, 774–783.

69. Oestereich, L., Müller-Kräuter, H., Pallasch, E., and Strecker, T. (2023). Passive Transfer of Animal-Derived Polyclonal Hyperimmune Antibodies Provides Protection of Mice from Lethal Lassa Virus Infection. Viruses *15*, 1436. https://doi.org/10.3390/v15071436.

70. Jamali, K., Käll, L., Zhang, R., Brown, A., Kimanius, D., and Scheres, S.H.W. (2024). Automated model building and protein identification in cryo-EM maps. Nature *628*, 450–457.

71. Hu, J.K., Crampton, J.C., Cupo, A., Ketas, T., van Gils, M.J., Sliepen, K., de Taeye, S.W., Sok, D., Ozorowski, G., Deresa, I., et al. (2015). Murine Antibody Responses to Cleaved Soluble HIV-1 Envelope Trimers Are Highly Restricted in Specificity. J. Virol. *89*, 10383–10398.

72. Corrigan, A.R., Duan, H., Cheng, C., Gonelli, C.A., Ou, L., Xu, K., DeMouth, M.E., Geng, H., Narpala, S., O'Connell, S., et al. (2021). Fusion peptide priming reduces immune responses to HIV-1 envelope trimer base. Cell Rep. *35*, 108937.

73. Kulp, D.W., Steichen, J.M., Pauthner, M., Hu, X., Schiffner, T., Liguori, A., Cottrell, C.A., Havenar-Daughton, C., Ozorowski, G., Georgeson, E., et al. (2017). Structure-based design of native-like HIV-1 envelope trimers to silence non-neutralizing epitopes and eliminate CD4 binding. Nat. Commun. *8*, 1655.

74. Duan, H., Chen, X., Boyington, J.C., Cheng, C., Zhang, Y., Jafari, A.J., Stephens, T., Tsybovsky, Y., Kalyuzhniy, O., Zhao, P., et al. (2018). Glycan Masking Focuses Immune Responses to the HIV-1 CD4-Binding Site and Enhances Elicitation of VRC01-Class Precursor Antibodies. Immunity *49*, 301–311.e5.

75. Turner, H.L., Andrabi, R., Cottrell, C.A., Richey, S.T., Song, G., Callaghan, S., Anzanello, F., Moyer, T.J., Abraham, W., Melo, M., et al. (2021). Disassembly of HIV envelope glycoprotein trimer immunogens is driven by antibodies elicited via immunization. Sci. Adv. *7*, eabh2791. https://doi.org/10.1126/sciadv.abh2791.

76. Turner, H.L., Pallesen, J., Lang, S., Bangaru, S., Urata, S., Li, S., Cottrell, C.A., Bowman, C.A., Crowe, J.E., Jr., Wilson, I.A., and Ward, A.B. (2019). Potent anti-influenza H7 human monoclonal antibody induces separation of hemagglutinin receptor-binding head domains. PLoS Biol. *17*, e3000139.

77. Bangaru, S., Lang, S., Schotsaert, M., Vanderven, H.A., Zhu, X., Kose, N., Bombardi, R., Finn, J.A., Kent, S.J., Gilchuk, P., et al. (2019). A Site of Vulnerability on the Influenza Virus Hemagglutinin Head Domain Trimer Interface. Cell *177*, 1136–1152.e18.

78. Cai, C.X., Doria-Rose, N.A., Schneck, N.A., Ivleva, V.B., Tippett, B., Shadrick, W.R., O'Connell, S., Cooper, J.W., Schneiderman, Z., Zhang, B., et al. (2022). Tyrosine O-sulfation proteoforms affect HIV-1 monoclonal antibody potency. Sci. Rep. *12*, 8433.

79. Pejchal, R., Walker, L.M., Stanfield, R.L., Phogat, S.K., Koff, W.C., Poignard, P., Burton, D.R., and Wilson, I.A. (2010). Structure and function of broadly reactive antibody PG16 reveal an H3 subdomain that mediates potent neutralization of HIV-1. Proc. Natl. Acad. Sci. USA *107*, 11483–11488.

80. Lee, J.H., Andrabi, R., Su, C.-Y., Yasmeen, A., Julien, J.-P., Kong, L., Wu, N.C., McBride, R., Sok, D., Pauthner, M., et al. (2017). A Broadly Neutralizing Antibody Targets the Dynamic HIV Envelope Trimer Apex via a Long, Rigidified, and Anionic β-Hairpin Structure. Immunity *46*, 690–702.

81. Ward, A.B., and Wilson, I.A. (2020). Innovations in structure-based antigen design and immune monitoring for next generation vaccines. Curr. Opin. Immunol. *65*, 50–56.

82. Banchereau, J., de Paoli, P., Vallé, A., Garcia, E., and Rousset, F. (1991). Long-term human B cell lines dependent on interleukin-4 and antibody to CD40. Science *251*, 70–72.

83. Pettersen, E.F., Goddard, T.D., Huang, C.C., Meng, E.C., Couch, G.S., Croll, T.I., Morris, J.H., and Ferrin, T.E. (2021). UCSF ChimeraX: Structure visualization for researchers, educators, and developers. Protein Sci. *30*, 70–82.

84. Punjani, A., Rubinstein, J.L., Fleet, D.J., and Brubaker, M.A. (2017). cryoSPARC: algorithms for rapid unsupervised cryo-EM structure determination. Nat. Methods *14*, 290–296.

85. Suloway, C., Pulokas, J., Fellmann, D., Cheng, A., Guerra, F., Quispe, J., Stagg, S., Potter, C.S., and Carragher, B. (2005). Automated molecular microscopy: the new Leginon system. J. Struct. Biol. *151*, 41–60.

86. Zheng, S.Q., Palovcak, E., Armache, J.-P., Verba, K.A., Cheng, Y., and Agard, D.A. (2017). MotionCor2: anisotropic correction of beam-induced motion for improved cryo-electron microscopy. Nat. Methods *14*, 331–332.

87. Zivanov, J., Nakane, T., Forsberg, B.O., Kimanius, D., Hagen, W.J., Lindahl, E., and Scheres, S.H. (2018). New tools for automated high-resolution cryo-EM structure determination in RELION-3. Elife *7*, e42166. https://doi.org/10.7554/eLife.42166.

88. Emsley, P., and Cowtan, K. (2004). Coot: Model-building tools for molecular graphics. Acta Crystallogr. D Biol. Crystallogr. *60*, 2126–2132.

89. Liebschner, D., Afonine, P.V., Baker, M.L., Bunkóczi, G., Chen, V.B., Croll, T.I., Hintze, B., Hung, L.W., Jain, S., McCoy, A.J., et al. (2019). Macromolecular structure determination using X-rays, neutrons and electrons: recent developments in Phenix. Acta Crystallogr. D Struct. Biol. *75*, 861–877.

90. Barad, B.A., Echols, N., Wang, R.Y.-R., Cheng, Y., DiMaio, F., Adams, P.D., and Fraser, J.S. (2015). EMRinger: side chain-directed model and map validation for 3D cryo-electron microscopy. Nat. Methods *12*, 943–946.

91. Chen, V.B., Arendall, W.B., 3rd, Headd, J.J., Keedy, D.A., Immormino, R.M., Kapral, G.J., Murray, L.W., Richardson, J.S., and Richardson, D.C. (2010). MolProbity: all-atom structure validation for macromolecular crystallography. Acta Crystallogr. D Biol. Crystallogr. *66*, 12–21.

92. Lander, G.C., Stagg, S.M., Voss, N.R., Cheng, A., Fellmann, D., Pulokas, J., Yoshioka, C., Irving, C., Mulder, A., Lau, P.-W., et al. (2009). Appion: an integrated, database-driven pipeline to facilitate EM image processing. J. Struct. Biol. *166*, 95–102.

93. Leem, J., Dunbar, J., Georges, G., Shi, J., and Deane, C.M. (2016). ABodyBuilder: Automated antibody structure prediction with data-driven accuracy estimation. mAbs *8*, 1259–1268.

94. Montiel-Garcia, D., Rojas-Labra, O., Santoyo-Rivera, N., and Reddy, V.S. (2022). Epitope-Analyzer: A structure-based webtool to analyze broadly neutralizing epitopes. J. Struct. Biol. *214*, 107839.

95. Agirre, J., Iglesias-Fernández, J., Rovira, C., Davies, G.J., Wilson, K.S., and Cowtan, K.D. (2015). Privateer: software for the conformational validation of carbohydrate structures. Nat. Struct. Mol. Biol. *22*, 833–834.

96. Kwakkenbos, M.J., van Helden, P.M., Beaumont, T., and Spits, H. (2016). Stable long-term cultures of self-renewing B cells and their applications. Immunol. Rev. *270*, 65–77.

97. Kwakkenbos, M.J., Diehl, S.A., Yasuda, E., Bakker, A.Q., van Geelen, C.M.M., Lukens, M.V., van Bleek, G.M., Widjojoatmodjo, M.N., Bogers, W.M.J.M., Mei, H., et al. (2010). Generation of stable monoclonal antibody-producing B cell receptor-positive human memory B cells by genetic programming. Nat. Med. *16*, 123–128.

98. Grobben, M., van der Straten, K., Brouwer, P.J., Brinkkemper, M., Maisonnasse, P., Dereuddre-Bosquet, N., Appelman, B., Lavell, A.A., van Vught, L.A., Burger, J.A., et al. (2021). Cross-reactive antibodies after SARS-CoV-2 infection and vaccination. Elife *10*, e70330. https://doi.org/10.7554/eLife.70330.

99. McCoy, L.E., van Gils, M.J., Ozorowski, G., Messmer, T., Briney, B., Voss, J.E., Kulp, D.W., Macauley, M.S., Sok, D., Pauthner, M., et al. (2016). Holes in the Glycan Shield of the Native HIV Envelope Are a Target of Trimer-Elicited Neutralizing Antibodies. Cell Rep. *16*, 2327–2338.

100. van Haaren, M.M., McCoy, L.E., Torres, J.L., Lee, W., Cottrell, C.A., Copps, J.L., van der Woude, P., Yasmeen, A., de Taeye, S.W., Torrents de la Peña, A., et al. (2021). Antibodies from Rabbits Immunized with HIV-1 Clade B SOSIP Trimers Can Neutralize Multiple Clade B Viruses by Destabilizing the Envelope Glycoprotein. J. Virol. *95*, e0009421.

101. Brouwer, P.J.M., Antanasijevic, A., Berndsen, Z., Yasmeen, A., Fiala, B., Bijl, T.P.L., Bontjer, I., Bale, J.B., Sheffler, W., Allen, J.D., et al. (2019). Enhancing and shaping the immunogenicity of native-like HIV-1 envelope trimers with a two-component protein nanoparticle. Nat. Commun. *10*, 4272.

102. Zivanov, J., Nakane, T., and Scheres, S.H.W. (2020). Estimation of high-order aberrations and anisotropic magnification from cryo-EM data sets in -3.1. IUCrJ *7*, 253–267.

103. Punjani, A., Zhang, H., and Fleet, D.J. (2020). Non-uniform refinement: adaptive regularization improves single-particle cryo-EM reconstruction. Nat. Methods *17*, 1214–1221.

104. Punjani, A., and Fleet, D.J. (2021). 3D variability analysis: Resolving continuous flexibility and discrete heterogeneity from single particle cryo-EM. J. Struct. Biol. *213*, 107702.

105. Bepler, T., Morin, A., Rapp, M., Brasch, J., Shapiro, L., Noble, A.J., and Berger, B. (2019). Positive-unlabeled convolutional neural networks for particle picking in cryo-electron micrographs. Nat. Methods *16*, 1153–1160.

106. Emsley, P., and Crispin, M. (2018). Structural analysis of glycoproteins: building N-linked glycans with Coot. Acta Crystallogr. D Struct. Biol. *74*, 256–263.

107. Wang, R.Y.-R., Song, Y., Barad, B.A., Cheng, Y., Fraser, J.S., and DiMaio, F. (2016). Automated structure refinement of macromolecular assemblies from cryo-EM maps using Rosetta. Elife *5*, e17219. https://doi.org/10.7554/eLife.17219.

STAR★METHODS

KEY RESOURCES TABLE

REAGENT or RESOURCE	SOURCE	IDENTIFIER
**Antibodies**		
Mouse anti-rabbit IgG-PE	Southern Biotech	Cat# 4090-09
Mouse anti-human IgG Fc-PE	Southern Biotech	Cat# 9040-09
12.1F	Robinson et al.[15]	Patent WO2018106712A1
25.10C	Robinson et al.[15]	Patent WO2018106712A1
37.7H	Robinson et al.[15]	Patent WO2018106712A1
8.9F	Robinson et al.[15]	Patent WO2018106712A1
18.5C	Robinson et al.[15]	Patent WO2018106712A1
LAVA05	This study	N/A
LAVA06	This study	N/A
25.10C Fab (TwinStrep-tagged)	This study	N/A
8.9F Fab (TwinStrep- or His-tagged)	This study	N/A
18.5C Fab (His-tagged)	This study	N/A
LAVA05 Fab	This study	N/A
LAVA06 Fab	This study	N/A
Polyclonal Fab from rabbit #350	This study	N/A
Polyclonal Fab from rabbit #187-192	This study	N/A
**Bacterial and virus strains**		
NEB 5-alpha Competent E.coli (High Efficiency	New England Biolabs	Cat# C2987H
Lassa virus (Josiah strain)	Müller et al.[53]	N/A
**Biological samples**		
Rabbit serum from rabbit #187-192	Brouwer et al.[30]	N/A
Purified polyclonal IgG from rabbit #350	Müller et al.[53]	N/A
**Chemicals, peptides, and recombinant proteins**		
GPCysR4(Josiah)-I53-50A.1NT1-StrepTagII	This study	N/A
GPCysRRLL(Josiah)-I53-50A.1NT1-StrepTagII	This study	N/A
GPCysR4(Josiah)-StrepTagII	This study	N/A
GPCysRRLL(Josiah)-I53-50A.1NT1-His-Avi	This study	N/A
Streptavidin AF647	Biolegend	Cat# 405237
Streptavidin BV421	Biolegend	Cat# 405225
Interleukin-21	R&D	8879-IL
T5 exonuclease	New England Biolabs	Cat# M0363
Phusion polymerase	New England Biolabs	Cat# M0530S
TaqDNA ligase	New England Biolabs	Cat# M0208S
NAD+	New England Biolabs	Cat# B9007S
dNTPs	New England Biolabs	Cat# N0447L
PEIMax	Polysciences	Cat# 24765-2
CellTrace CFSE	Invitrogen	Cat# C34554
Tween 20	Sigma-Aldrich	Cat# P1379-500ML
Bovine Serum Albumin	Thermo Fisher Scientific	Cat# 9048-46-8
Penicillin	Sigma-Aldrich	Cat#P3032-10ML
Streptomycin	VWR	Cat# 382-EU-100G
PBS	Thermo Fisher Scientific	Cat# 10010023
TBS	Alfa Aesar	Car# J60764.K2
Uranyl formate	Electron Microscopy Sciences	Cat #D310 25 GM

(*Continued on next page*)

Continued		
REAGENT or RESOURCE	SOURCE	IDENTIFIER
Fluorinated octyl maltoside	Anatrace	Part# O310F
Lauryl maltose neopentyl glycol	Anatrace	Part# NG310
Imidazole	Sigma-Aldrich	Cat# I5513
Papain from papaya latex	Sigma-Aldrich	SKU #P4762
2-Mercaptoethanol	Gibco	Cat# 21985-023
Fugene	Promega	Cat# E5911
DEAE-dextran	Sigma-Aldrich	Cat# D9885
Saquinavir	NIH-ARP	Cat# 4658
Reporter lysis buffer	Promega	Cat# E3971
Trypan Blue	Sigma-Aldrich	T8154-100ML
Critical commercial assays		
BrightGlo Luciferase Assay System	Promega	Cat# E2620
SCRIPT HF RT-PCR Kit	Jena Bioscience	Cat# PCR-510
HotStarTaq Plus polymerase	Qiagen	Cat# 203605
Deposited data		
Maps of GPC with pAbs from rabbit 187 (nsEM)	This study	EMDB: EMD-43174 to EMD-43175
Map of GPC with pAbs from rabbit 188 (nsEM)	This study	EMDB: EMD-43176
Maps of GPC with pAbs from rabbit 189 (nsEM)	This study	EMDB: EMD-43177 to EMD-43178
Maps of GPC with pAbs from rabbit 190 (nsEM)	This study	EMDB: EMD-43179
Map of GPC with pAbs from rabbit 191 (nsEM)	This study	EMDB: EMD-43180
Maps of GPC with pAbs from rabbit 192 (nsEM)	This study	EMDB: EMD-43181
Map of GPC with mAb LAVA05 (nsEM)	This study	EMDB: EMD-43182
Map of GPC with mAb LAVA05 (nsEM)	This study	EMDB: EMD-43183
Map and model of GPC with mAb 8.9F (cryo-EM)	This study	EMDB: EMD-45624, PDB: 9CJ7
Map and model of GPC with pAb GPC-A-2 (cryo-EM)	This study	EMDB: EMD-45644, PDB: 9CK8
Map and model of GPC with pAb GPC-A-1 (cryo-EM)	This study	EMDB: EMD-45643, PDB: 9CK7
Map and model of GPC with pAb LL-1 (cryo-EM)	This study	EMDB: EMD-45625, PDB: 9CJ8
Map and model of GPC with pAb Base-1 (cryo-EM)	This study	EMDB: EMD-41713, PDB: 8TYC
Map of GPC with pAb Base-2 (cryo-EM)	This study	EMDB: EMD-45905
Map and model of GPC with pAb FP-1 (cryo-EM)	This study	EMDB: EMD-41715, PDB: 8TYE
Map of monomeric GPC with 25.10C, INT-1 and INT-2 (cryo-EM)	This study	EMDB: EMD-41716
Map and model of GPC with pAb GPC-C-1 (cryo-EM)	This study	EMDB: EMD-43141, PDB: 8VCV
Map and model of GPC with pAb GP1-A-1 (cryo-EM)	This study	EMDB: EMD-43168, PDB: 8VE8
Experimental models: Cell lines		
Irradiated mouse fibroblast expressing CD40-ligand	Banchereau et al.[82]	N/A
HEK 293T cells	ATCC	Cat# CRL-11268
FreeStyle 293F cells	Thermo Fisher Scientific	Cat# R79007
TZM-bl cells	NIH ARRRP	Cat# 8129
Vero E6 cells	ATCC	Cat# CRL-1587
Recombinant DNA		
pFUSE-rIgG-Fc	Invivogen	Cat# pfuse-rfc1
GPCysR4(Josiah)-StreptagII pPPI4 plasmid	Brouwer et al.[30]	N/A
GPCysR4(Josiah)-I53-50A.1NT1-Strep-tagII pPPI4 plasmid	Brouwer et al.[30]	N/A
GPCysRRLL(Josiah)-I53-50A.1NT1-Strep-tagII pPPI4 plasmid	Perrett et al.[31]	N/A
GPCysRRLL(Josiah)-I53-50A.1NT1-Avi-His pPPI4 plasmid	This study	N/A
GPCysR4(Josiah)-I53-50A.1NT1-Avi-His pPPI4 plasmid	This study	N/A
GPC(Josiah)_full-length pcDNA3.0 plasmid	Brouwer et al.[30]	N/A
GPC(Josiah)_full-length E228KD229Y pcDNA3.0 plasmid	This study	N/A

(*Continued on next page*)

Continued

REAGENT or RESOURCE	SOURCE	IDENTIFIER
S1P pcDNA3.0 plasmid	Perrett et al.[31]	N/A
Furin pPPI4 plasmid	Brouwer et al.[30]	N/A
12.1F HC, 12.1F LC	Brouwer et al.[30]	N/A
25.10C HC, 25.10C LC	Brouwer et al.[30]	N/A
37.7H HC, 37.7H LC	Brouwer et al.[30]	N/A
8.9F HC, 8.9F LC	Brouwer et al.[30]	N/A
LAVA01 HC, LAVA01 LC	Brouwer et al.[30]	N/A
LAVA05 HC, LAVA05 LC	This study	N/A
LAVA06 HC, LAVA06 LC	This study	N/A
25.10C HC-TwinStrep-tag	This study	N/A
18.5C HC-His-tag	This study	N/A
8.9F HC-His-tag	This study	N/A
8.9F HC-TwinStrep-tag	This study	N/A
Software and algorithms		
GraphPad Prism v8	GraphPad	N/A
UCSF ChimeraX	Pettersen et al.[83]	N/A
CryoSPARC	Punjani et al.[84]	N/A
Leginon	Suloway et al.[85]	N/A
MotionCor2	Zheng et al.[86]	N/A
Relion/3.0	Zivanov et al.[87]	N/A
Coot	Emsley et al.[88]	N/A
Phenix	Liebschner et al.[89]	N/A
EMRinger	Barad et al.[90]	N/A
MolProbity	Chen et al.[91]	N/A
Appion	Lander et al.[92]	N/A
ABodyBuilder	Leem et al.[93]	N/A
Epitope-Analyzer	Montiel-Garcia et al.[94]	N/A
Privateer	Agirre et al.[95]	N/A
FlowJo	BD Lifesciences	N/A
ModelAngelo	Jamali et al.[70]	N/A
Others		
DMEM/F-12	Gibco	Cat# 31330-038
Magpix	Luminex	N/A
MagPlex®-C Microspheres	Luminex	MC100xx-01
Protein G resin	Cytiva	Cat# 17061802
CaptureSelect IgG-Fc resin	Thermo Fisher Scientific	Cat# 2942852010
Q5 Site-directed mutagenesis kit	New England Biolabs	Cat# E0554S
Superdex200 10/300GL Column	GE Healthcare Life Sciences	Cat# 28990944
Ni-NTA agarose	QIAGEN	Cat# 30210
Amicon® Ultra-4 Centrifugal Filter Unit (100 kDA MWCO)	Millipore Sigma	SKU# UFC810024
Amicon® Ultra-4 Centrifugal Filter Unit (30 kDA MWCO)	Millipore Sigma	SKU# UFC803024
Amicon® Ultra-4 Centrifugal Filter Unit (10 kDA MWCO)	Millipore Sigma	SKU# UFC801024
Amicon Ultra-0.5 Centrifugal Filter Unit (100 kDa MWCO)	Millipore Sigma	SKU# UFC5100BK
Amicon Ultra-0.5 Centrifugal Filter Unit (30 kDa MWCO)	Millipore Sigma	SKU # UFC5030BK
Amicon Ultra-0.5 Centrifugal Filter Unit (10 kDA MWCO)	Millipore Sigma	SKU# UFC5010BK
Prometheus NT.Plex nanoDSF Grade High Sensitivity Capillary Chips	Nanotemper	Cat# PR-AC006
400-mesh copper grids	Electron Microscopy Sciences	Cat# 0400-Cu
Octet Red96 system	Sartorius (FortéBio)	N/A

(*Continued on next page*)

Continued

REAGENT or RESOURCE	SOURCE	IDENTIFIER
Fetal calf serum	Gibco	Cat# 10270/106
Fetal bovine serum	Omega Scientific Inc.	FB-02
L-glutamine	Gibco	Cat# 25030-081
Octet Biosensors: Anti-Human Fc Capture (AHC)	Sartorius (FortéBio)	Cat# 18-5060
Octet Bionsensors: NiNTA	Sartorius (FortéBio)	Cat# 18-5101
FreeStyle 293 Expression medium	Thermo Fisher Scientific	Cat# 12338018
OptiMEM	Gibco	Cat# 31985-070
DMEM	Gibco	Cat# 21969-035
BXT Buffer (10X)	IBA Lifesciences	Cat# 2-1042-025
Steritop Filter Units	Merck Millipore	Cat# C3239
Glomax reader	Turner BioSystems	Model# 9101-002
UltrAuFoil R1.2/1.3 grids (300-mesh)	Quantifoil Micro Tools GmbH	N/A
Graphene Oxide on Quantifoil R1.2/1.3 Cu (400 mesh)	Electron Microscopy Sciences	Cat# GOQ400R1213Cu50
Cu-grids (400-mesh)	Electron Microscopy Sciences	Cat# EMS400-Cu
Strep-TactinXT 4flow resin	IBA Life Sciences	Cat# 2-5010-025
NanoDrop 2000C	Thermo Fisher Scientific	Cat# ND-2000C
Prometheus NT.48 NanoDSF	NanoTemper Technologies	N/A
Tecnai Spirit electron microscope	FEI	N/A
Vitrobot mark IV	Thermo Fisher Scientific	N/A
Solarus 950 plasma system	Gatan	N/A
PELCO easiGlow	Ted Pella Inc.	N/A
FEI Titan Krios	Thermo Fisher Scientific	N/A
Glacios cryo-TEM	Thermo Fisher Scientific	N/A
Glacios II cryo-TEM	Thermo Fisher Scientific	N/A
K2 Summit direct electron detector camera	Gatan	N/A
Falcon 4i direct electron detector camera	Thermo Fisher Scientific	N/A
Falcon 4 direct electron detector camera	Thermo Fisher Scientific	N/A

EXPERIMENTAL MODEL AND STUDY PARTICIPANT DETAILS

Cell lines

FreeStyle 293F (Thermo Fisher Scientic) and HEK293T (ATCC) are human embryonic kidney cell lines optimized for enhanced production of recombinant proteins or retroviruses. Freestyle 293F cells are adapted for suspension culture and are grown at 37°C with 8% CO2, shaking at 125 rpm, in 293FreeStyle expression medium (Thermo Fisher Scientic). HEK293T cells are maintained in Dulbecco's Modified Eagle's Medium (DMEM; Gibco) supplemented with 10% fetal calf serum (FCS) (Gibco), penicillin (100 U/mL), and streptomycin (100 mg/mL), and are cultured statically at 37°C with 8% CO2. The TZM-bl cell line is an indicator cell line highly sensitive to diverse isolates of HIV-1, allowing for quantitative analysis of HIV infection using either β-galactosidase or luciferase as reporters. The parental cell line (JC.53) stably expresses large amounts of CD4 and CCR5 and constitutively expresses CXCR4. TZM-bl cells were maintained in DMEM, supplemented with 10% FCS, penicillin (100 U/mL), and streptomycin (100 mg/mL). VeroE6 cells were cultured at 37°C with 5% CO2 in DMEM (Gibco) supplemented with 10% FCS (Gibco), 50 U/mL pen/strep (Gibco), and 2 mM L-glutamine (Gibco). Cells were passaged twice a week to maintain a density of 1–1.3 × 10^6 cells/mL.

METHOD DETAILS

Construct design

The GPCysR4-I53-50A, and GPCysR4 monomer constructs, which contain a C-terminal Strep-tag were generated as previously described and feature the strain Josiah GPC sequence (GenBank: NP_694870.1).[30] GPCysR4-I53-50A containing a His-Avi-tag was generated by substituting the TwinStrep-tag with the following sequence by Q5-site directed mutagenesis: HHHHHHGGLNDIFEAQKIEWHE. To generate the GPCysRRLL-I53-50A plasmid the R258L and R259L mutations were introduced in GPCysR4-I53-50A (both the Strep-tag as the His-Avi-tagged construct) by Q5 site-directed mutagenesis. Plasmids of 37.7H, 12.1F, 25.10C, and 8.9F were generated using Gibson assembly, which involved inserting genes encoding the variable regions of the corresponding heavy and light chains into plasmids containing the constant regions of the human IgG1 for the heavy or light chain.

To express Fabs (see below) we generated plasmids encoding TwinStrep- or His-tagged 8.9F HC, His-tagged 18.5C HC, and TwinStrep-tagged 25.10C HC by introducing the respective tags (directly followed by a stop codon) by Q5 site-directed mutagenesis in the hinge region, as previously described.[30,31] The GPC mutant 228K229Y for pseudovirus neutralization assays was generated by introducing the E228K and D229Y mutations in the full-length native Josiah GPC plasmid (kind gift from Robin Shattock) by Q5 site-directed mutagenesis.

Protein expression and purification

GPCysRRLL, GPCysR4 trimers and GPCysR4 monomer

GPCysRRLL-I53-50A, GPCysR4-I53-50A, and GPCysR4 were transiently expressed in Freestyle 293F cells at a density of 1.0 × 10^6 cells/mL. Ratios of 1:3 DNA to PEImax were used to facilitate co-transfection of plasmids encoding Strep-tagged GPCysRRLL-I53-50A, GPCysR4-I53-50A, or GPCysR4 with plasmids encoding S1P (in the case of GPCysRRL-I53-50A) or furin (in the case of GPCysR4-I53-50A or GPCysR4). A ratio of 2:1 GPC to protease plasmid DNA was used. Freestyle 293F cells were cultured and kept shaking at 125 rpm for five days at 37°C and 8% CO2. Cultures were harvested after six days and GPCysRRLL/R4-I53-50As and GPCysR4 were purified via Strep-tag purification using gravity columns and StrepTactin 4Flow resin (IBA Life Sciences), following the manufacturer's protocol. The proteins were eluted using 1X BXT buffer (IBA Life Sciences) and buffer exchanged to 1X TBS using 100 kDa MWCO concentrators for trimers and 10 kDa MWCO concentrators for monomers (Millipore). As a final purification step, size exclusion chromatography was performed using Superdex 200 increase 10/300 GL columns (Sigma-Aldrich) with TBS as the running buffer. Fractions associated with the elution volume corresponding to trimeric (in the case of GPCysRRLL/R4-I53-50A) or monomeric GPC (in the case of GPCysR4) were collected and concentrated using either 100 kDa or 10 kDa MWCO concentrator, respectively (Millipore).

8.9F, 18.5C, and 25.10C Fab

8.9F, 18.5C and 25.10C Fabs were expressed in Freestyle 293F cells (Thermo Fisher Scientic) using a transfection ratio of 2:1 LC to HC and a 3:1 PEI to DNA ratio. 8.9F HCs featured either a His-tag or a TwinStrep-tag, 18.5C HC encodes a His-tag, while 25.10C HC encodes a TwinStrep-tag. Cells were cultured for six days as mentioned above before being harvested by centrifugation. Depending on the tag, Fabs were purified using StrepTactin 4Flow resin (IBA Life Sciences) or Ni-NTA resin (Thermo Fisher Scientic). TwinStrep-tagged 8.9F and 25.10C Fab were purified via gravity column before being eluted by 1X BXT (IBA Life Sciences) while His-tagged 8.9F and 18.5C Fab culture supernatants were rolled overnight with Ni-NTA beads at 4°C before using gravity columns to capture the resin. This resin was washed with 20 mM imidazole, 50 mM NaCl, pH 7.0 buffer before being eluted with a 500 mM imidazole, 50 mM NaCl, pH 7.0 buffer. All Fabs were buffer exchanged to TBS using 10 kDa MWCO concentrators (Millipore).

IgG

8.9F, 12.1F, 25.10C, 37.7H, LAVA05, and LAVA06 IgGs were expressed as previously described.[30,31] In brief, IgG plasmids were transfected at an HC to LC ratio of 1:1 in Freestyle 293F cells (Thermo Fisher Scientic). Cells were cultured and harvested by centrifugation five days post-transfection. The IgGs were purified by adding Protein G (Cytiva) or CaptureSelect IgG-Fc resin (Thermo Fisher Scientic) to the supernatant and rolling overnight at 4°C. Resin was captured by gravity column and IgG eluted with 0.1 M glycine at pH 2.0 into 1 M Tris pH 8.0. All IgGs were immediately buffer exchanged to TBS using 30 kDa MWCO concentrators (Millipore).

GPCysRRLL-I53-50A + 8.9F Fab complexes

Complexes of 8.9F Fab and GPCysRRLL-I53-50A were expressed and purified directly to remove the need for final size exclusion chromatography steps. In these cases, 500 μg purified 8.9F Fabs with a TwinStrep-tag were added to transfected Freestyle 293F cells (see above; Thermo Fisher Scientic) expressing GPCysRRLL-I53-50A with a His-Avi-tag at day three post-transfection. After harvesting cells at day six post-transfection, 8.9F-GPCysRRLL-I53-50A complexes were purified using StrepTactin 4Flow resin as mentioned previously and then buffer exchanged to TBS using 100 kDa MWCO concentrators (Millipore).

Complexes of 8.9F Fab and GPCysRRLL-I53-50A were also generated using parallel Freestyle 293F (Thermo Fisher Scientic) transfections. In these cases, Freestyle 293F cells were split in a 1:4 ratio and grown to 1.0 × 10^6 cells/mL. The smaller batch of cells were transfected with a 2:1 ratio of 8.9F LC and HC plasmid DNA. The HC plasmid DNA featured a TwinStrep-tag (as described above in *Construct design*). The larger batch of cells was transfected with GPCysRRLL-I53-50A with a His-Avi-tag as previously described. On day three post-transfection, Freestyle 293F cells expressing 8.9F Fab were added to the culture expressing GPCysRRLL-I53-50A. Cultures were harvested at day six post-transfection and complexes were purified using StrepTactin 4Flow resin (Thermo Fisher Scientic) and eluted with 1X BXT. Complexes were immediately buffer exchanged to TBS using 100 kDa MWCO concentrators (Millipore).

GPCysRRLL-I53-50A + 18.5C Fab and GPCysR4+25.10C Fab complexes

Complexes of 18.5C Fab and GPCysRRLL-I53-50A were made by mixing purified GPCysRRLL/R4-I53-50A with 18.5C Fab in a 1:20 M ratio, followed by an incubation at 4°C for 1 h. Complexes of 25.10C Fab and GPCysR4 were generated by incubating purified GPCysR4 with purified 25.10C Fab in a 1:20 M ratio for 1 h at 4°C.

Isolation of GPC-specific B cells

Rabbit peripheral blood mononuclear cells (PBMC), obtained from a previously described immunization study,[30] were thawed in cold DMEM/F12 medium (Gibco) containing 8% FBS and pen/strep. Next, B cells were stained with mouse-*anti*-rabbit IgG-PE (Southern

Biotech, diluted 500x in ice-cold PBS with 1% FBS) after which they were isolated by flow cytometry (BD, FACSAria). Since the rabbits were vaccinated with recombinant GPCysR4-I53-50A[30] we excluded I53-50A-binding B cells by including a SARS-CoV-1 Spike probe containing I53-50A-biotin which was labeled with streptavidin AF647 and streptavidin BV421 (both Biolegend). Sorted cells (I53-50A-negative) were then cultured for 2 days in DMEM/F12 media supplemented with interleukin (IL)-21 and irradiated mouse fibroblast (L cells) expressing CD40-ligand.[82] B cells were then immortalized by retroviral transduction with BcL6, Bcl-XL and the marker gene GFP, as previously described.[96,97] Subsequently, the cells were cultured at 1 cell per well in 96 well plates. After 10 days, culture supernatant containing secreted monoclonal antibody were screened for binding to GPCysR4-I53-50A (strain Josiah) using a custom Luminex assay similar to previously described.[98] In short, GPCysR4-I53-50A was covalently coupled to Luminex Magplex beads with a two-step carbodiimide reaction at a ratio of 75 μg protein to 12.5 million beads. GPCysR4-I53-50A coupled beads and B cell culture supernatant were incubated overnight, washed and incubated with mouse-*anti*-rabbit IgG-PE (Southern Biotech) to detect antibodies in culture supernatant that bound GPCysR4-I53-50A on the beads. Readout was performed on a Magpix (Luminex). Positive clones were collected, and a cell pellet was stored for VH and VL sequence determination and cloning.

Antibody cloning

First, according to the Jena Bioscience protocol a combined reverse transcription (RT) and first PCR reaction was performed using either heavy chain or kappa chain primers on 1 μL of a 100x diluted freeze-thawed cell pellet of the B cell clone of interest. In the SCRIPT HF RT-PCR Kit (Jena Bioscience), 0.5 μL of primers from a 10 mM stock as described by McCoy et al. were included.[99] The RT reaction was performed for 1 h at 60°C and 5 min at 5°C after which the first PCR reaction was started immediately for 40 cycles at [95°C for 10 s, 20 s at 55°C, 1 min at 72°C] and ending with 2 min at 72°C. Subsequently a second heavy and kappa chain PCR was performed which included 0.25 μM of primer mix. The primers all include a Gibson overhang which is necessary for cloning of the VH and VL into our rabbit IgG1 expression vectors which are based on the pFUSE-rIgG-Fc (InvivoGen) vectors.[99,100] For this second PCR, 0.4 μL of the first PCR was used in a reaction using HotStarTaq Plus polymerase (Qiagen) for 5 min at 95°C and 30 cycles of [30 s at 94°C, 30 s at 60°C, and 1 min at 72°C], and 10 min at 72°C. Gibson cloning was then used to integrate the amplified heavy and light chain V(D)J variable regions in mammalian cell expression vectors containing the rabbit constant regions. This was done by mixing 1 μL of expression vector, 1 μL of the second PCR product, and 2 μL of home-made Gibson mix (T5 exonuclease (0.2U; New England Biolabs), Phusion polymerase (12.5U; New England Biolabs), TaqDNA ligase (2000U; New England Biolabs), Gibson reaction buffer (0.5 g PEG-8000; Sigma Life Sciences), 1 M Tris/HCl pH 7.5, 1 M MgCl2, 1 M DTT, 100 mM dNTPs, 50 mM NAD + (New England Biolabs) MQ) and incubating the mix for 60 min at 50°C.

Antibody binding to membrane expressed GPC

HEK293T cells were transfected with 8 μg of full-length native GPC (strain Josiah) expressed in the pPPI4 vector using a 1:3 ratio of DNA to PEImax. A day prior, $3x10^6$ HEK293T cells per Petri dish were seeded in 8 mL DMEM (Gibco) supplemented with 8% FBS and pen/strep. Cells were harvested after approximately 36 h and frozen at $2x10^6$ per vial. For the flow cytometry assay non-transfected HEK293T cells were labeled with the CFSE CellTrace while the Josiah expressing cells were not labeled. Next, cells were incubated with a dilution range starting at 10 μg/mL of purified monoclonal antibodies either from human or rabbit origin. Antibody binding was detected either with mouse anti-human-PE or anti-rabbit IgG-PE (Southern Biotech) and detected using the FACS Symphony (BD). Analysis was performed using FlowJo software.

Antibody binding by biolayer interferometry

All assays were performed at 25°C with dilutions made in the manufacturer-recommended running buffer (PBS, 0.1% BSA, 0.02% Tween 20, pH 7.4). Data was analyzed using the Octet Data Analysis software where running buffer references were subtracted from all data.

GPCysRRLL-I53-50A characterization

Ab binding to recombinant GPCyRRLL-I53-50A was determined using an Octet Red96 instrument (ForteBio). 8.9F, 12.1F, 25.10C, and 37.7H IgG were diluted to concentrations of 10 μg/mL and loaded onto Protein A sensors (Sartorius) to a binding signal of 1.0 nM. Sensors were dipped into wells containing 120 nM GPCysRRLL-I53-50A for 600 s then dipped into running buffer to measure dissociation for 600 s.

Screening of isolated monoclonal antibodies

To screen for base-specific monoclonal antibodies His-tagged 8.9F Fab at 10 μg/mL was loaded onto NiNTA sensors, after which sensors were dipped into wells containing 120 nM of GPCysRRLL-I53-50A for 600s. Next, sensors were dipped into wells containing 50 μg/mL of 25.10C Fab until a plateau was reached. This was done to shield off as much of any non-base epitope as possible. The sensors were then transferred to running buffer for 100 s to ensure there was no off-rate. After this short dissociation step, sensors were dipped into 10 μg/mL of purified monoclonal antibody isolated from rabbits (see *Isolation of GPC-specific B cells* and *Antibody cloning* above) for 600 s. Antibodies that bound were selected for screening by nsEM.

Antibody binding by LAVA05 and LAVA06

To measure binding by base-specific mAbs, His-tagged 8.9F Fab at 10 μg/mL were loaded onto NiNTA sensors, after which sensors were dipped into wells containing 120 nM of GPCysRRLL-I53-50A for 600 s. After a dissociation step of 600 s (only a minor off-rate

was observed which reached a plateau within 600 s), the sensor was dipped into a well containing 10 μg/mL LAVA05 or LAVA06 for 600s and then dipped into running buffer to measure dissociation for 300 s.

SDS-PAGE analysis

SDS-PAGE were conducted according to previously established protocols. 2.5 μg of or GPCysR4-I53-50A or GPCysRRLL-I53-50A was combined with loading dye, with or without β-mercaptoethanol, denatured, and then loaded onto a 10–20% Tris-Glycine gel (Thermo Fisher Scientic). Next, the gel was washed several times with MQ and developed using SimplyBlue SafeStain (Thermo Fisher Scientic).

Differential scanning fluorimetry

Thermostability of GPCysR4-I53-50A, GPCysRRLL-I53-50A, and GPCysRRLL-I53-50A in complex with 8.9F was determined with a nano-DSF NT.48 (Prometheus). GPC proteins or complexes were diluted to 0.5 mg/mL and loaded into high sensitivity capillaries. The assay was run with a linear scan rate of 1 °C/min and 80%–100% excitation power. The first derivative of the ratio of tryptophan fluorescence wavelength emissions at 350 and 330 nM were analyzed to determine the denaturation (T_m) temperatures using the Prometheus NT software.

Fab preparation for EMPEM studies

For the EMPEM studies described here, material from previously described studies Brouwer et al.[30] (rabbit serum) and Müller et al.[35] (purified polyclonal rabbit IgG) was used. In the case of the former, first, IgGs were isolated from sera using 2 mL CaptureSelect IgG-Fc resin slurry (Thermo Fisher Scientic) per 1 mL rabbit sera. Sera and resin were gently mixed at 4°C for a minimum of two overnights. After, resin was collected by a gravity-flow column and washed three times with 1X PBS, pH 7.4 to remove IgG-depleted sera. IgGs were eluted from resin twice with 9 mL of 0.1 M glycine, pH 2.0 buffer and immediately neutralized with 1mL 1 M Tris, pH 8.0. The elutions were then buffer exchanged to PBS using a 30 kDa MWCO concentrator (Millipore). Purified polyclonal IgGs as well as purified monoclonal IgGs were digested to Fab following previously described protocols.[54,55,57] In summary, IgGs were digested by incubating the IgGs with papain (Sigma-Aldrich) in freshly prepared digestion buffer (20 mM sodium phosphate, 10 mM EDTA, 20 mM L-cysteine, pH 7.4). IgGs were digested at 37°C for 4–5 h before the reaction was quenched using iodoacetamide at a final concentration of 0.03 M. Two mL of CaptureSelect IgG-Fc resin slurry was added to digested IgG samples and incubated for a minimum of 2 h at 4°C. CaptureSelect IgG-Fc resin bound to Fc were separated from Fab mixtures by a gravity-flow column. The Fc-depleted flow-through was buffer exchanged to TBS using 10 kDa MWCO concentrators (Millipore). For sera from rabbits immunized with GPCysR4-I53-50A, to remove I53-50A-specific antibody responses, the polyclonal Fabs were incubated with BG505 SOSIP scaffolded on I53-50 (BG505-I53-50A; construct design and expression described previously[101]) at a ratio of 1 mg purified Fab to 400 μg BG505-I53-50A. The mixture was incubated overnight at room temperature and run through a 100 kDa MWCO concentrators (Millipore) so the flow-through was depleted of Fabs specific to the I53-50A scaffold. The flow-through was finally concentrated using a 10 kDa MWCO concentrator (Millipore).

Preparation of GPC immune complexes with polyclonal rabbit Fabs

For nsEMPEM as well as cryo-EM with graphene oxide grids, immune complexes were generated by mixing 20 μg of GPCysRRLL-I53-50A pre-complexed with 8.9F or 18.5C Fabs, as described above, with 600 μg of purified polyclonal Fab (in the case of GPCysR4-I53-50A sera, already depleted of I53-50A-specific Fabs). To generate immune complexes for cryo-EM where UltrAuFoil R1.2/1.3 grids were used (collections for Base-1 and Base-2) a higher concentration of immune complex was required to freeze grids. For these experiments we therefore complexed 200 μg of GPCysRRLL-I53-50A, pre-complexed with 8.9F Fab, with 2000 μg of purified polyclonal Fab (already depleted of I53-50A-specific Fabs). Complexes were incubated for 4 h at 4°C after which they were purified from unbound/excess Fab by size exclusion chromatography using a Superose 200 increase column with TBS as the running buffer. Fractions corresponding to immune complexes (~9.8 mL–11.2 mL) were pooled and concentrated using a 100 kDa MWCO centrifugal filter (Millipore). To generate immune complexes for EMPEM experiments with GPC monomers, 20 μg of GPCysR4 pre-complexed with 25.10C Fab was complexed with 600 μg of purified polyclonal Fab (for cryo-EM this was pooled polyclonal Fab from rabbits 187–192). Excess Fab was removed by size exclusion chromatography using a Superose 200 increase column with TBS as the running buffer. Fractions corresponding to immune complexes (~11 mL–13 mL) were pooled and concentrated using a 10 kDa MWCO centrifugal filter (Millipore).

Preparation of GPC immune complexes with LAVA05 and LAVA06

To generate complexes, 50 μg of GPCysRRLL-I53-50A, pre-complexed with 8.9F Fab, was incubated with 200 μg of LAVA05 or LAVA06 Fab for 2 h at 4°C. Next, the mixture was run through a 100 kDa MWCO centrifugal filter to remove excess Fab. The filter was then buffer exchanged several times with TBS to remove any residual uncomplexed Fab. Concentrated complex was then collected for nsEM experiments.

Negative stain electron microscopy

Carbon-coated Cu-grids (400-mesh) were glow-discharged for 25 s at 15 mA using a PELCO easiGlow instrument (Ted Pella, Inc.). Samples were diluted to 15 μg/mL in TBS and loaded onto the grid for 30 s. After blotting off the sample, the grid was immediately stained with 2% (w/v) uranyl formate for 20–40 s. After blotting off excess stain, grids were dried for at least 5 min. Imaging was performed on a Tecnai Spirit electron microscope (operating at 120 keV, nominal magnification was 52,000×, resulting pixel size at the specimen plane was 2.05 Å). Electron dose was set to 25 e−/Å2 and the nominal defocus for imaging was −1.50 μm. The Leginon automated imaging software[85] was used for data acquisition.

Negative stain electron microscopy data processing

Particles were picked using Appion,[92] after which Relion 3.0 was used for particles extraction, 2D/3D classification and 3D refinement.[87] Extracted particles were subjected to one to two rounds of 2D classification to select for GPCysRRLL-I53-50A-8.9F Fab or GPCysRRLL/GPCysR4-I53-50A-18.5C Fab complexes with a pFab bound. As none of the GPCysR4-I53-50A-vaccinated rabbits showed any pFab binding to GPCysR4-I53-50A-18.5C complexes we did not continue to generate 3D maps from these 2D classes. In addition, as we directly pursued cryo-EM, we also did not generate 3D maps of the immune complexes from sera of rabbit 350. The following therefore only applies to GPCysRRLL-I53-50A-8.9F Fab complexed with pFab. Once a minimum of 30,000 particles were selected from 2D classification, 3D classification was performed using a GPCysRRLL-I53-50A-8.9F complex as an initial model. This model was generated in ChimeraX[83] by low-pass filtering the following ensemble: a GPCysRRLL-8.9F complex (PDB: 9CJ7) to which we docked in a full Fab (PDB: 4ZTP[65]) so that the complex would contain the constant domain) and a correctly positioned I53-50A scaffold (PDB: 7SGE[30]). After a first round of 3D classification, output maps were aligned to the initial model in ChimeraX using the Volume Fit tool.[83] Correct alignment of the, at this resolution, almost featureless GPC is aided by the asymmetrical binding of 8.9F to GPC and the lobe-like structures of the protomers. Particles of classes that showed pFab(s) bound to the same epitope clusters were combined and subjected to another round of 3D classifications. This process was repeated until a well-defined GPC complexed with 8.9F and a pFab was visible. The combined particle stack was then further processed using 3D auto-refinement. Composite models of GPC with pFabs bound, as seen in Figures 2 and 3, were generated in ChimeraX by fitting both the refined 3D map as well as a low-pass filtered smoothened map of GPC to the initial model. All density apart from the pFab was then erased from the refined 3D map. For more information on generated maps and EMDB IDs see Table S2.

Generation of LASV pseudovirus

LASV pseudoviruses were generated as previously described.[30] HEK 293T cells were grown in DMEM with 10% FCS, penicillin (100 U/mL), and streptomycin (100 mg/mL) in 6-well plates to 80% confluence. For each well, a 1:25 dilution of Fugene in OptiMEM (250 μL) was mixed with 0.6 μg of a full-length GPC expression plasmid and 2.4 μg of SG3env in OptiMEM (250 μL). After a 20 min incubation at RT, the mix was added to the well. After 72 h, the supernatant was harvested, sterile filtered, aliquoted, and stored at −80°C. Virus titers were determined via a TCID50 assay. TZM-bl cells were grown in DMEM with 10% FCS, penicillin (100 U/mL), and streptomycin (100 mg/mL) in 96-well plates to 70–80% confluence. Pseudovirus stocks were serially diluted, incubated at RT for 1 h, and added to the cells with DEAE-dextran (40 μg/mL) and Saquinavir (400 nM). After a 72 h incubation at 37°C, cells were lysed with Reporter Lysis Buffer (Promega) and luciferase signal was measured using Bright Glo Luciferase buffer (Promega) and a Glomax plate reader. Pseudovirus input for neutralization assays was determined as the dilution yielding luciferase counts of 500,000 (>10x above background).

Pseudovirus neutralization assay

TZM-bl cells were maintained in DMEM (Gibco) supplemented with 10% FCS, penicillin (100 U/mL), and streptomycin (100 mg/mL), and grown overnight in a 96-well plate to 70–80% confluence. The next day, 3-fold serial dilutions of rabbit serum (1:40 start concentration) or mAbs (20 μg/mL start concentration) were incubated with pseudovirus (strain Josiah or Josiah 228K229Y) for 1 h. All dilutions were performed in DMEM (Gibco) supplemented with 10% FCS, penicillin (100 U/mL), and streptomycin (100 mg/mL). The virus/mAb mixture was then added to TZM-bl cells, which had been pre-supplemented with DEAE-dextran and Saquinavir (Sigma-Aldrich), as described for the TCID50. After a 72 h incubation at 37°C, cells were lysed with Reporter Lysis Buffer (Promega) and luciferase signal was measured using Bright Glo Luciferase buffer (Promega) and a Glomax plate reader. Luciferase counts were normalized to those obtained from cells infected with pseudovirus in the absence of serum/mAbs.

Authentic neutralization assay

Authentic LASV (strain Josiah) neutralization assays were conducted in the BSL-4 laboratory at the Institute of Virology, Philipps University Marburg, Germany. Rabbit sera were inactivated with complement for 30 min at 56°C and subjected to a 2-fold dilution series, starting at a dilution of 16. The diluted sera were then incubated with 100 TCID50 virus for 60 min at 37°C. Subsequently, Vero E6 cell (ATCC) suspension was added, and the plates were incubated at 37°C with 5% CO_2. Cytopathic effects (CPE) were assessed seven days post-infection, and neutralization titers were determined as the geometric mean titer (GMT) of four replicates.

Cryo-EM grid preparation and data collection

For cryo-EM of GPCysRRLL-I53-50A with 8.9F Fab as well as GPCysRRLL-I53-50A-8.9F Fab complexes with pFab from rabbit 190 (maps of Base-1 and Base-2), UltrAuFoil R1.2/1.3 grids (Au, 300-mesh; Quantifoil Micro Tools GmbH) were plasma discharged using a PELCO easiGlow (Ted Pella Inc.) for 25 s at 15 mA. Fluoro-octyl maltoside or lauryl maltose neopentyl glycol (Anatrace) were added to protein samples immediately before their applications to grids at final concentrations of 0.02 w/v% and 5 μM, respectively. Grids were plunge-frozen using a Vitrobot Mark IV (Thermo Fisher Scientific) with chamber temperature set to 4°C and humidity to 100%. Blot times between 3 and 5 s, blot force of 1 and a wait time of 10 s were used for all samples. Sample concentrations were between 0.6 and 4.0 mg/mL. For all other complexes described, Quantifoil R1.2/1.3 grids (Cu, 400-mesh; Electron Microscopy Sciences) coated with graphene oxide were used. Sample, at a concentration of 0.1 mg/mL was loaded directly on the grid (not plasma discharged) in the absence of detergent and plunge-frozen using a Vitrobot Mark IV (Thermo Fisher Scientific) with chamber temperature set to 4°C and humidity to 100%. Blot time of 2.5 s, blot force of 0 and a wait time of 30 s were used.

Grids were loaded into a 300 kV FEI Titan Krios or 200 kV Thermo Fisher Scientific Glacios (II). The Titan Krios was equipped with a K2 Summit direct electron detector camera (Gatan) while the Glacios and Glacios II featured a Falcon 4 and Falcon 4i detector, respectively (Thermo Fisher Scientific). Data collection was automated using Leginon (for Titan Krios)[85] and EPU software (for Glacios (II)). Additional information is noted in Table S3.

Cryo-EM data processing

Image preprocessing was performed either with the Appion software package[92] or CryoSPARC Live software.[84] For the former, micrograph movie frames were aligned and dose-weighted with UCSF MotionCor 2 software[86] before being transferred to CryoSPARC.[84] When CryoSPARC Live software was used, image motion corrections and initial CTF estimations were performed directly within that software package before transfer to CryoSPARC for further processing. Particles were picked and classified into 2D classes. Good initial 2D classes were chosen for template picking of particles. This particle stack was iteratively 2D classified to remove bad particle picks.

GPCysRRLL-I53-50A bound to 8.9F

We used an initial model generated in UCSF ChimeraX[83] from low-pass filtered GPC structure PDB: 7SGD.[30] Particles were initially refined using homogeneous and heterogeneous refinements to select for 3D classes with a good distribution of orientations. After initial alignments, we performed a focused 3D classification of the 8.9F antibody and selected particles based on their resolution at the epitope-paratope interface. Iterative rounds of global and local CTF refinements paired with local refinements were used to obtain a high resolution map.[102] All refinements were performed with C1 symmetry due to the asymmetric engagement of 8.9F Fab.

GPCysRRLL-I53-50A bound to pFabs from GPCysR4-I53-50A-vaccinated rabbits

A low pass filtered structure of the 8.9F-GPCysRRLL-I53-50A complex was used as the initial model or an initial model was generated from ab initio refinement of the 2D classified particle stack. Particles were first aligned to their shared GPC features through homogeneous refinements, in some cases followed by an NU-refinement job.[103] Aligned particles were symmetry expanded along the C3 axis in accordance with the trimeric nature of GPC and to simplify further processing. As such, further alignments were restrained by using only local refinement (with C1 symmetry) and 3D classification/3D variability analyses to prevent symmetry-related particle copies from aligning to themselves.[104] 3D classification/variability analyses employing sphere masks around putative epitope clusters were based on nsEMPEM data. Clusters with pFab densities were further refined using iterative local refinements paired with 3D classification/variability analyses to obtain particle stacks with the best epitope-paratope density. Soft solvent masks encompassing GPC and the pFab were used for local refinements post-3D classification/variability analysis. Finally, one or two rounds of global CTF refinement followed by local refinement were performed to obtain a high resolution map.

GPCysRRLL-I53-50A bound to pFabs from GPC-derived VLP-vaccinated rabbits

Heterogeneous refinement was first performed to classify and refine a homogeneous stack of particles, using an initial model generated from ab initio refinement of the 2D classified particle stack. The particle stack corresponding to a GPCysRRLL-I53-50A-18.5C complex with three pFabs bound (2x GP1-A-, 1x GPC-C-like response) was then subjected to a round of non-uniform refinement after which 3D classification was performed using a sphere mask around the epitope-paratope interface.[103] This was done separately for both the GP1-A-like epitope-paratope interface and the GPC-C-like epitope-paratope interface. 3D classes with the highest resolution particles were selected and subjected to multiple rounds of non-uniform refinement, separately for both pFab responses, using a soft solvent mask around the GPCysRRLL in complex with the pFab in question. All refinements were performed with C1 symmetry due to the asymmetric engagement of 8.9F Fab.

GPCysR4 monomer bound to pFabs

After initial 2D classification, we used Topaz particle picking to identify monomeric GPC particles bound to pFabs.[105] We used initial models generated from ab initio refinements of the refined particle stack. Heterogeneous refinements enabled us to determine a class of immune complexes bound by three Fabs, which we further refined using iterative NU-refine and local refine jobs.[103] For the final refinement, we employed an initial model and mask that contained only a low-pass filtered monomeric GPC protomer with Fv regions of bound Fabs based on PDB: 7TYV.[40]

Atomic model refinement

Sharpened maps were used to build all final atomic models. All initial GPC models (derived from PDB: 7SGD,[30] PDB: 8EJD,[30,31] or 9CJ7) were manually fit using Coot.[88,106] For the generation of the GPCysRRLL-8.9F structure, the 8.9F initial model was generated using ABodyBuilder.[93] For the structures of GPC with pFabs, model building of the stabilizing Fab (8.9F or 18.5C) was not performed (the density was generally too diffuse anyway due to masking of merely the GPC and pFab in the final refinement). The initial models for pFabs were derived from PDB: 7RA7. The model 7RA7 was manually fit into densities for pFabs and based on the experimental density, residues were added or deleted from CDR loops. Conserved residues were maintained in the initial pFab models for early rounds of Rosetta refinement[107] followed by manual corrections in Coot using additional models (PDB: 4ZTP[65] PDB: 4ZTO; [65] and PDB: 7SGF[30]) to guide placement of loops. Once the fit of pFabs with conserved side chains was feasible, we re-defined all amino acids in the antibody sequence to alanine. Model fit to map was validated using Molprobity and EMRinger in the Phenix software package.[89–91] Final rounds of refinement were performed in Phenix (version 1.21) to improve the statistics for geometry restraints. Orientations of all glycans was assessed and corrected using Privateer.[95] The epitope-paratope interactions between GPCysRRLL and 8.9F were determined using Epitope-Analyzer.[94] To identify epitope footprints, we used an algorithm, generated in-house, that lists all atoms (and their respective residues) in a specified chain within a certain angstrom radius to any atom of another specified chain. A cut-off of 4 Å was used for peptidic contacts between GPCysR4/GPCysRRLL and mAbs (i.e., 8.9F, 12.1F, 25.10C, 37.7H), whereas for the pFabs (poly-A models) a cutoff of 6 Å was used to compensate for the absence of atoms past Cβ. Cα RMSD values comparing our 8.9F-GPCysRRLL-I53-50A structure to PDB: 7UOT was performed using UCSF ChimeraX's MatchMaker tool.[83] Values denoted in the text represent the pruned and unpruned pairs of all GP1 protomers with the 8.9F HC and LC and exclude glycans. Final atomic models were submitted to the Protein DataBank with accession codes found in Table S3. Figures including atomic models were all produced using UCSF ChimeraX, except for supplemental figures showing model and map overlays which were generated in Coot.[88]

ModelAngelo

ModelAngelo was installed from GitHub and model_angelo build_no_seq was used to build models from density maps of GP1-A-1 and GPC-C-1.[70] The output model (which consists of the most probable residue for each position) was then superimposed on the poly-alanine model. Residues in the poly-alanine model that seem to make interactions with GPC were then mutated in Coot to the most probable ModelAngelo prediction.

Cell Reports, Volume *43*

Supplemental information

Defining bottlenecks and opportunities for Lassa virus neutralization by structural profiling of vaccine-induced polyclonal antibody responses

Philip J.M. Brouwer, Hailee R. Perrett, Tim Beaumont, Haye Nijhuis, Sabine Kruijer, Judith A. Burger, Ilja Bontjer, Wen-Hsin Lee, James A. Ferguson, Martin Schauflinger, Helena Müller-Kräuter, Rogier W. Sanders, Thomas Strecker, Marit J. van Gils, and Andrew B. Ward

A

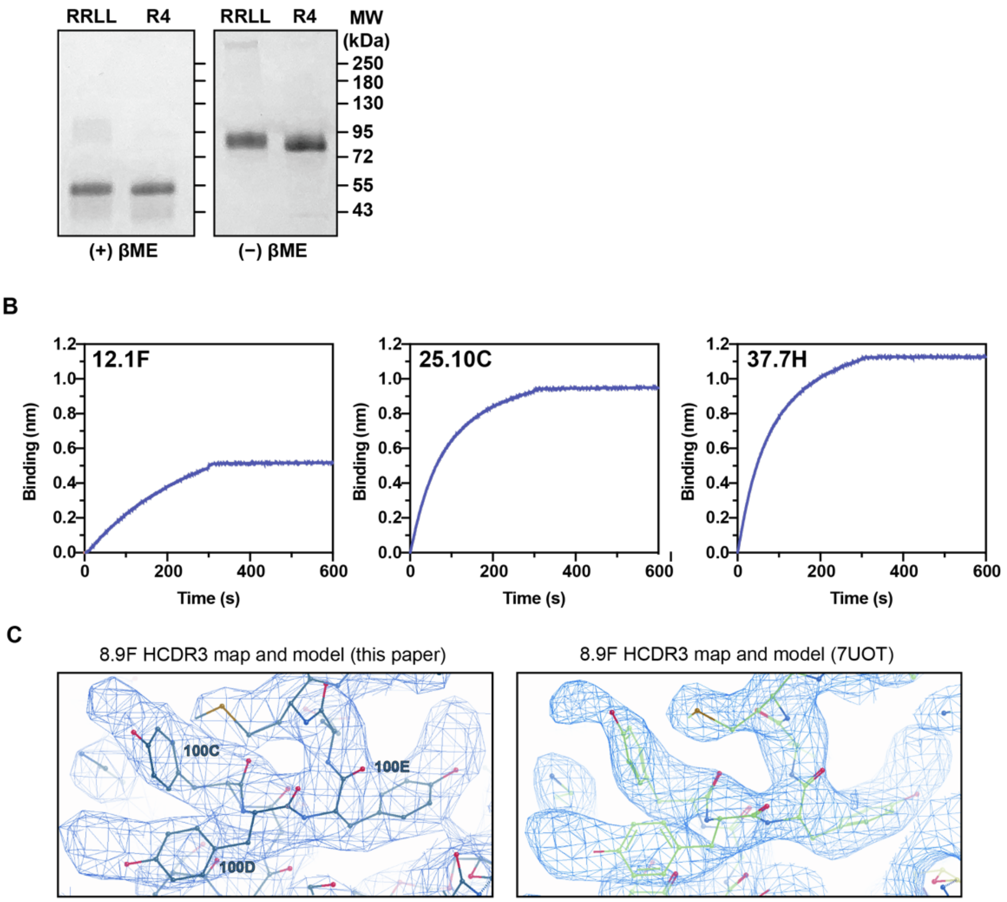

D

Protein / sequence name	Position	E-value	Sequence
8.9F HC	100C	[52]	KVADDYYYEMD
			+ ddY ++
	100D	[54]	VADDYY-YEMD
			dd Y ++
	100E	[54]	ADDYYYE---M
			+d Y+ +
	100Q	[15]	QGTDDYSYAMD
			+ +ddY a+

E

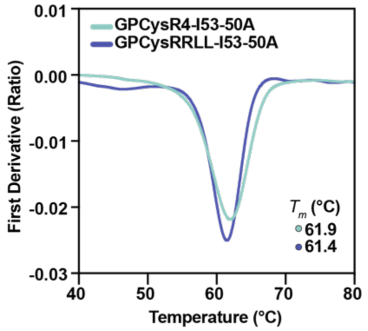

Figure S1. Characterization and comparison of GPCysRRLL-I53-50A and its interaction with 8.9F, related to Figure 1. (A) SDS-PAGE comparison of cleavage between GPCysRRLL-I53-50A co-transfected with S1P and GPCysR4-I53-50A co-transfected with furin in reducing (left) and non-reducing (right) conditions. Coomassie staining was used for protein visualization. **(B)** BLI sensorgrams indicating immobilized 12.1F, 25.10C, and 37.7H IgGs binding to GPCysRRLL-I53-50A at a concentration of 120 nM. **(C)** Close up of the model and maps described here (left) and 7UOT[1] (right), focusing on the HCDR3 of 8.9F around residues 100C, 100D, and 100E. **(D)** Result from Expasy's Sulfinator**.**[2] Presence of sulfated tyrosines is predict based on four different Hidden Markov models (HMM).[2] The position of predicted sulfated tyrosines are depicted in the second column. Lower case letters in the fourth column indicate direct matches with the HMM

chain and pluses mark positions with a positive score. **(E)** Thermostability of GPCysRRLL-I53-50A and GPCysR4-I53-50A assessed by nanoDSF. Melting temperatures (T_m) are calculated as the inflection point (circles) of the ratio of signal at 350 and 330 nM. Each melting curve is a representative of triplicate curves with T_m within ±0.1°C.

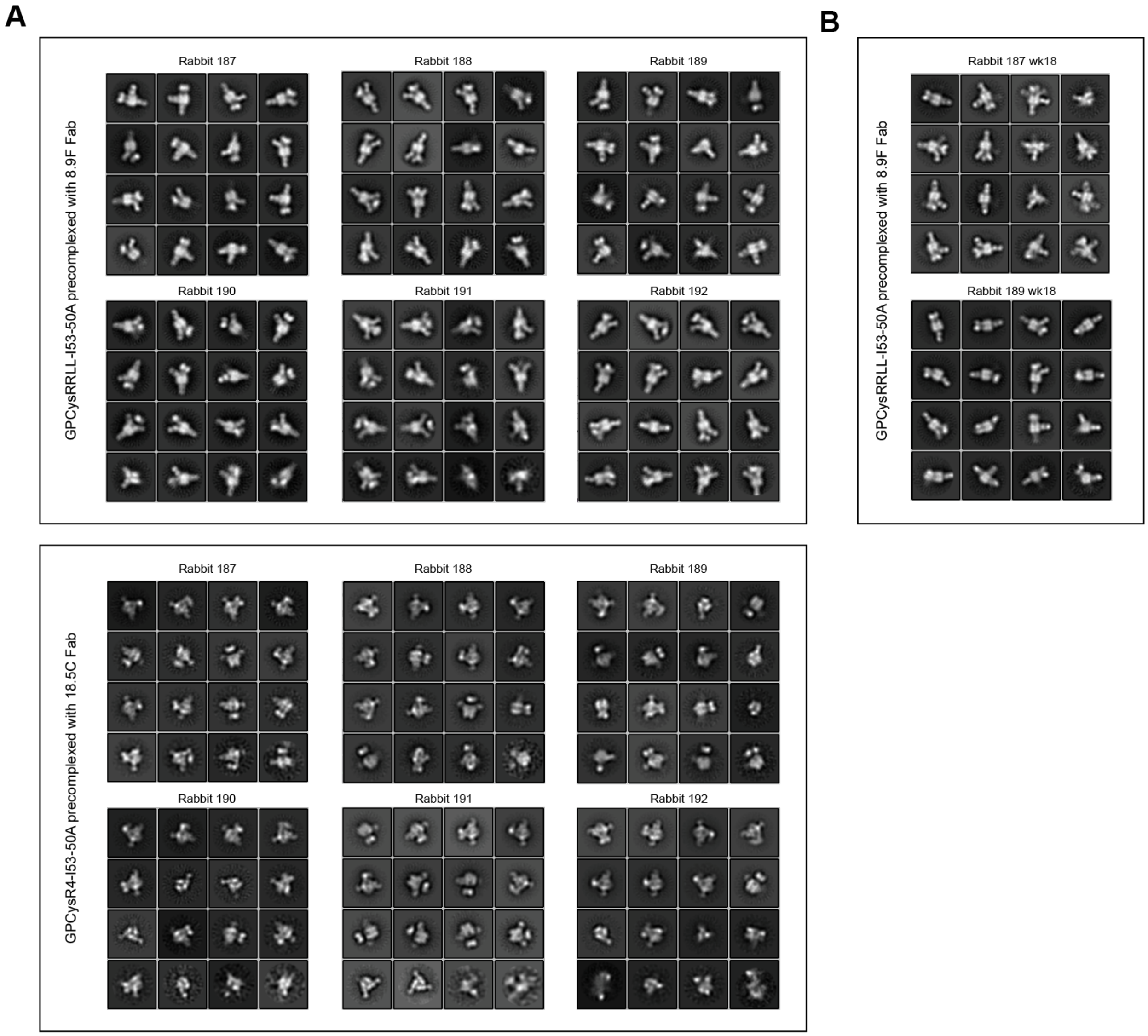

Figure S2. 2D classes of selected particles for the generation of 3D maps, related to Figure 2. (A) 2D class averages of nsEMPEM experiments with week 30 pAbs from rabbit 187-192 using GPCysRRLL-I53-50A pre-complexed with 8.9F (top) and GPCysR4-I53-50A pre-complexed with 18.5C (bottom). Classes shown are the selected classes that were selected for 3D classification, reclassified to 16 classes. **(B)** 2D class averages of nsEMPEM experiments with week 18 pAbs from rabbit 187 and 189 using GPCysRRLL-I53-50A pre-complexed with 8.9F. Classes shown are the selected classes that were selected for 3D classification, reclassified to 16 classes.

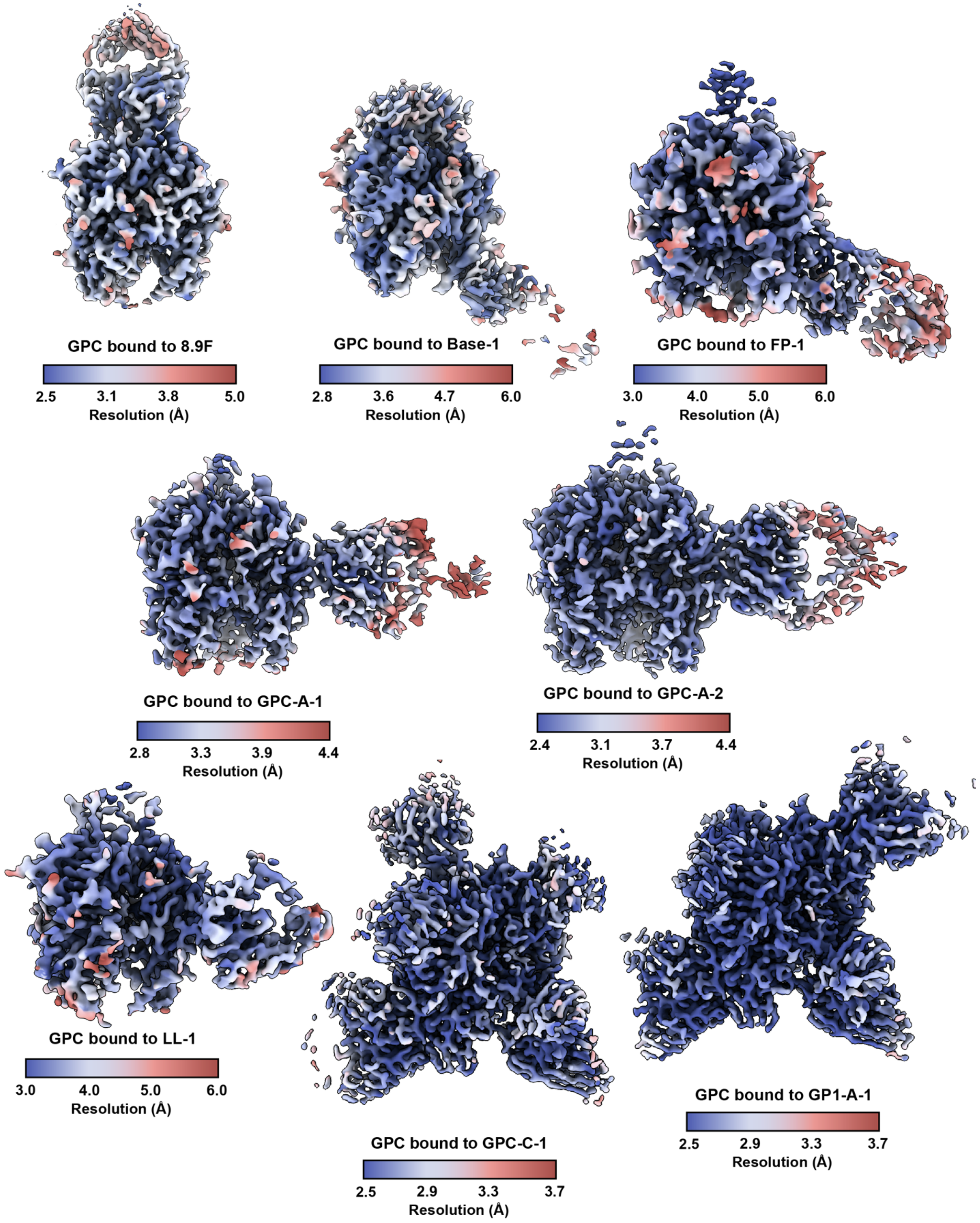

Figure S3. Local resolution for EM maps of LASV GPC and 8.9F, Base-1, FP-1, GPC-A-1, GPC-A-2, LL-1, GPC-C-1, and GP1-A-1, related to Figures 3, 4, and 6. Local resolutions calculated using cryoSPARC v4.5.3[3] using an FSC threshold of 0.143. Maps visualized in ChimeraX.[4]

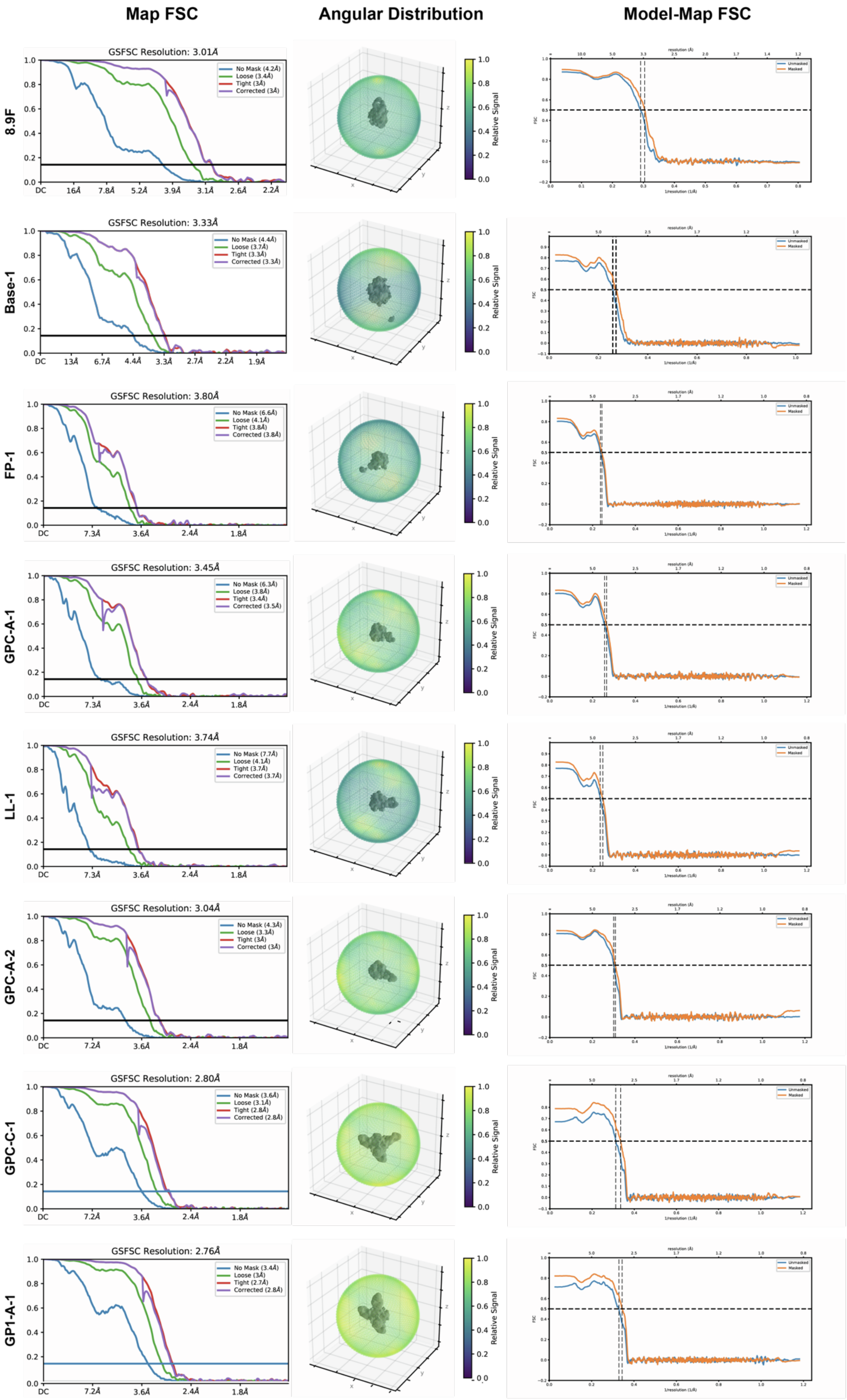
Map FSC
Angular Distribution
Model-Map FSC
8.9F
GSFSC Resolution: 3.01Å
No Mask (4.2Å)
Loose (3.4Å)
Tight (3Å)
Corrected (3Å)
Base-1
GSFSC Resolution: 3.33Å
No Mask (4.4Å)
Loose (3.7Å)
Tight (3.3Å)
Corrected (3.3Å)
FP-1
GSFSC Resolution: 3.80Å
No Mask (6.6Å)
Loose (4.1Å)
Tight (3.8Å)
Corrected (3.8Å)
GPC-A-1
GSFSC Resolution: 3.45Å
No Mask (6.3Å)
Loose (3.8Å)
Tight (3.4Å)
Corrected (3.5Å)
LL-1
GSFSC Resolution: 3.74Å
No Mask (7.7Å)
Loose (4.1Å)
Tight (3.7Å)
Corrected (3.7Å)
GPC-A-2
GSFSC Resolution: 3.04Å
No Mask (4.3Å)
Loose (3.3Å)
Tight (3Å)
Corrected (3Å)
GPC-C-1
GSFSC Resolution: 2.80Å
No Mask (3.6Å)
Loose (3.1Å)
Tight (2.8Å)
Corrected (2.8Å)
GP1-A-1
GSFSC Resolution: 2.76Å
No Mask (3.4Å)
Loose (3Å)
Tight (2.7Å)
Corrected (2.8Å)
Relative Signal
Unmasked
Masked

Figure S4. FSC plots for cryo-EM maps, angular distribution plots and model-map FSC plots for the maps that were used model building, related to Figures 3, 4, and 6. Reported resolutions of maps coincide with an FSC cutoff of 0.143. FSCs are presented from local refinement jobs except for GPC-C-1 and GP1-A-1 which come from a global refinement. FSC plots for maps (left) and angular distribution plots (center) were generated using cryoSPARC v4.5.3.[3] The model-map FCS plots (right) were generated using Phenix 1.21.[5]

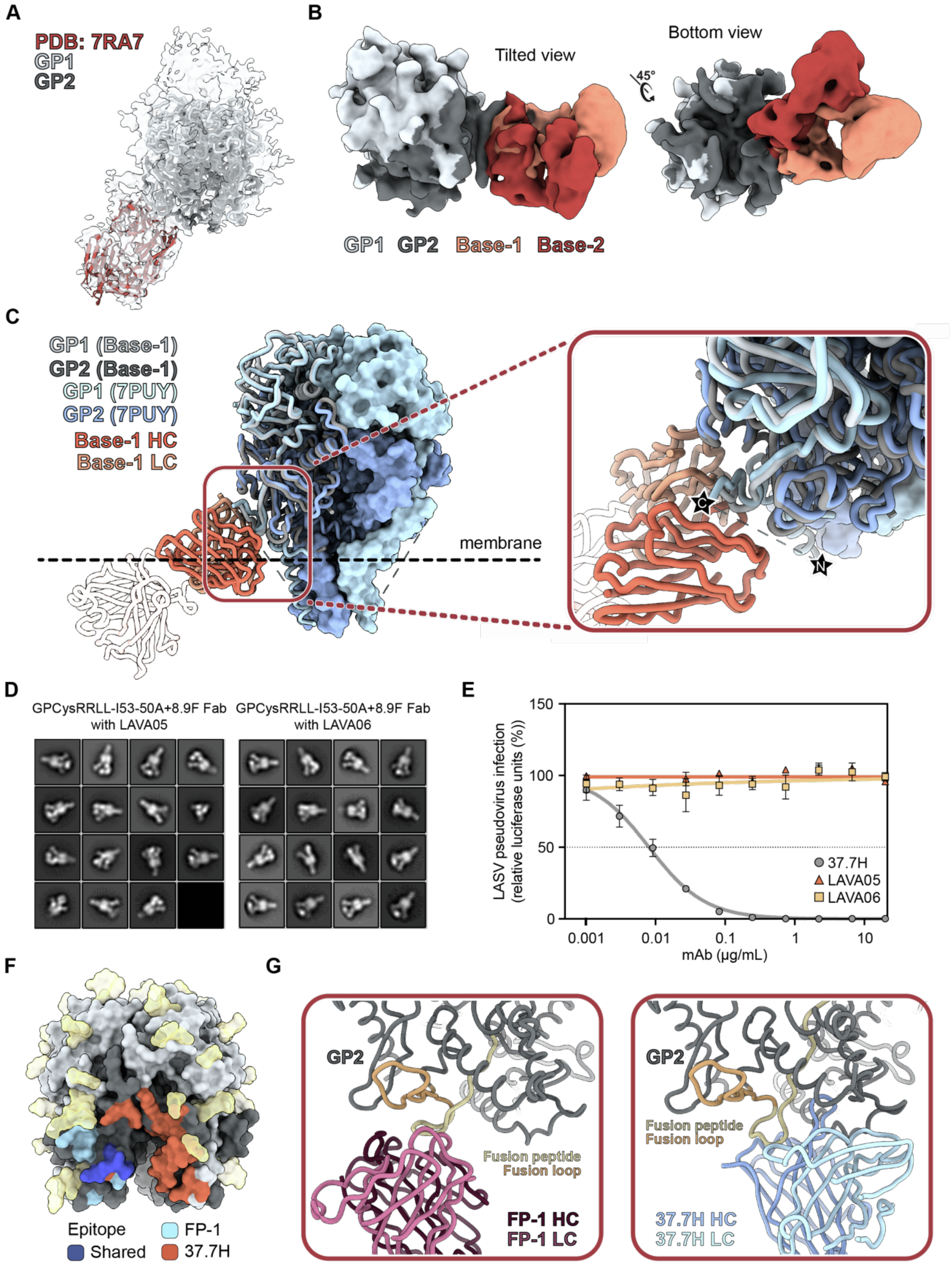

Figure S5. Structural differences between Base-1 and Base-2 responses, their inability to bind membrane-embedded GPC, neutralization of LAVA05 and LAVA06, and structural characterization of the FP-1 response, related to Figure 3. (A) Cryo-EM map of Base-2 with a rabbit Fab (PDB: 7RA7) docked in the pFab density. **(B)** Overlaid Gaussian-filtered maps of base antibodies demonstrating the distinct angles of approach taken by Base-1 (orange) and

Base-2 (red) to engage the GPC. **(C)** Overlay of full-length GPC (PDB: 7PUY[6]) with the Base-1 model revealing accessibility issues for Base-1 binding by the membrane and/or SSP. The constant region of the Fab (transparent) was generated by docking a model of a full rabbit Fab (PDB: 7RA7) into the density and removing the variable domain. The inset shows the unmodelled part of the SSP (displayed as pseudobond), referred to by Katz et al.[6] as "hydrophobic 2" (residues 36-58), with its N terminus (right) and C terminus (left) depicted as stars.[6] It is probable that the 22 amino acid "hydrophobic 2" structure resides in this area, sterically blocking binding of base-targeting pAb responses. **(D)** 2D class averages of nsEMPEM experiments with LAVA05 and LAVA05 using GPCysRRLL-I53-50A pre-complexed with 8.9F. Classes shown are the selected classes that were selected for 3D classification, reclassified to 16 classes. **(E)** Pseudovirus neutralization of LAVA05 and LAVA06. The mAb 37.7H was used as a positive control. Shown are the mean and SD of three technical replicates. **(F)** Comparison of the epitope footprint of FP-1 with prototypical GPC-B mAb 37.7H. To determine the footprint of 37.7H, all GPC residues within a radius of 4 Å from 37.7H were highlighted. For the footprint of FP-1, all GPC residues within a radius of 6 Å from FP-1 were highlighted to compensate for the absence of modeled sidechains past Cβ. **(G)** Close up of GP2 engagement by FP-1 (left) and 37.7H (right), illustrating the difference in fusion loop/fusion peptide engagement.

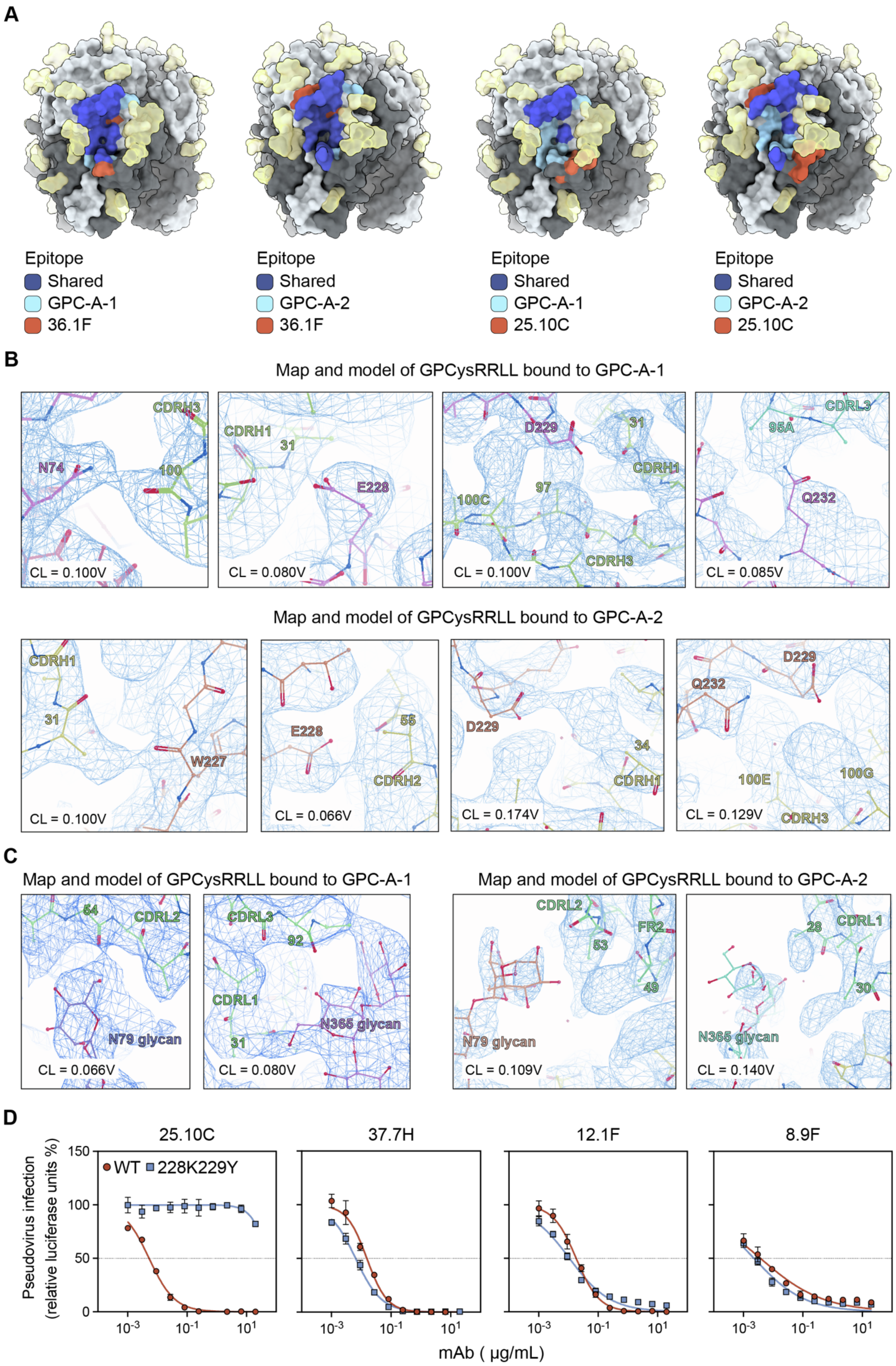
A
Epitope
Shared
GPC-A-1
36.1F
Epitope
Shared
GPC-A-2
36.1F
Epitope
Shared
GPC-A-1
25.10C
Epitope
Shared
GPC-A-2
25.10C
B
Map and model of GPCysRRLL bound to GPC-A-1
CDRH3
N74
100
CL = 0.100V
CDRH1
31
E228
CL = 0.080V
D229
31
CDRH1
100C
97
CDRH3
CL = 0.100V
CDRL3
95A
Q232
CL = 0.085V
Map and model of GPCysRRLL bound to GPC-A-2
CDRH1
31
W227
CL = 0.100V
E228
55
CDRH2
CL = 0.066V
D229
34
CDRH1
CL = 0.174V
D229
Q232
100E
100G
CDRH3
CL = 0.129V
C
Map and model of GPCysRRLL bound to GPC-A-1
54
CDRL2
N79 glycan
CL = 0.066V
CDRL3
92
CDRL1
31
N365 glycan
CL = 0.080V
Map and model of GPCysRRLL bound to GPC-A-2
CDRL2
FR2
53
49
N79 glycan
CL = 0.109V
28
CDRL1
30
N365 glycan
CL = 0.140V
D
25.10C
37.7H
12.1F
8.9F
WT
228K229Y
Pseudovirus infection
(relative luciferase units %)
150
100
50
0
10^{-3}
10^{-1}
10^{1}
mAb (μg/mL)

Figure S6. Characterization of the GPC-A-1 and GPC-A-2 response and effect of the 228K229Y mutation on the neutralization potency of representative NAbs from the various competition clusters, related to Figure 4. (A) Epitope footprints of GPC-A-1 and GPC-A-2 compared to NAbs 36.1F and 25.10C.[7] . To determine the footprint of 36.1F and 25.10C, all GPC residues within a radius of 4 Å from the mAb were highlighted. For the footprint of GPC-A-1 and GPC-A-2, all GPC residues within a radius of 6 Å from the pAb were highlighted to compensate for the absence of modeled sidechains past Cβ. **(B)** Close up of map and model where the density suggests contacts for GPC-A-1 (top) and GPC-A-2 (bottom) which are also shared contact residues for 36.1F and 25.10C on GPC. Contour level (CL) is indicated in each panel. **(C)** Close up of map and model where the density demonstrates interactions between glycans on GPC and the pAb GPC-A-1 (left) and GPC-A-2 (right). Contour level (CL) is indicated in each panel**. (D)** Neutralization curves for mAbs 25.10C (GPC-A competition cluster), 37.7H (GPC-B competition cluster), 12.1F (GP1-A competition cluster), and 8.9F (GPC-C competition cluster), against Josiah pseudovirus (WT) or Josiah pseudovirus with the 228K229Y mutation. Shown are the mean and SD of three technical replicates.

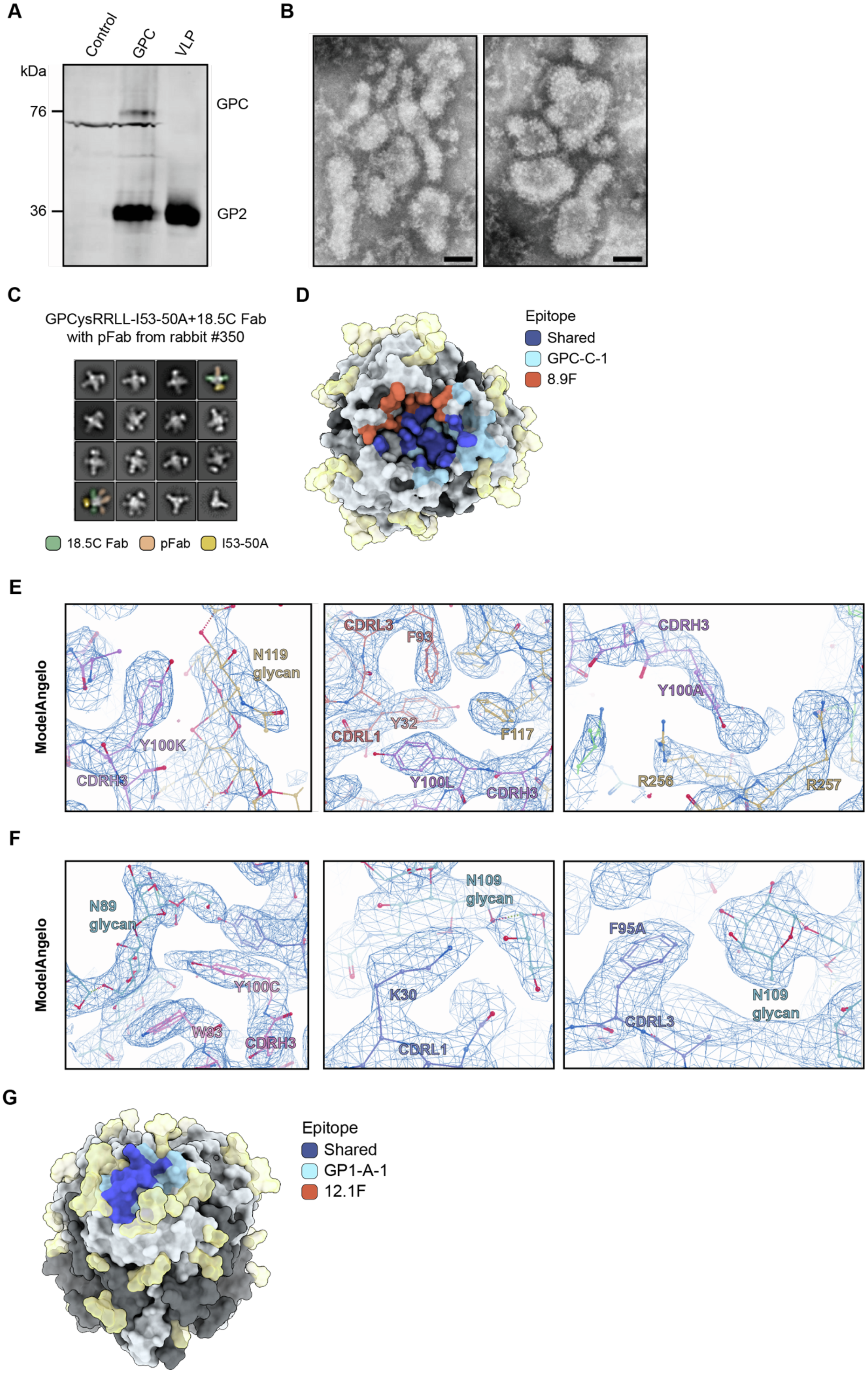

A
Control
GPC
VLP
kDa
76
36
GPC
GP2
B
C
GPCysRRLL-I53-50A+18.5C Fab
with pFab from rabbit #350
18.5C Fab
pFab
I53-50A
D
Epitope
Shared
GPC-C-1
8.9F
E
ModelAngelo
N119
glycan
Y100K
CDRH3
CDRL3
F93
Y32
CDRL1
F117
Y100L
CDRH3
CDRH3
Y100A
R256
R257
F
ModelAngelo
N89
glycan
Y100C
W93
CDRH3
N109
glycan
K30
CDRL1
F95A
N109
glycan
CDRL3
G
Epitope
Shared
GP1-A-1
12.1F

Figure S7. Characterization of a GPC-derived VLP and the humoral immune responses it elicited in rabbit 350, related to Figure 6. (A) Western blot analysis of GPC-derived VLPs purified from the cell culture supernatant of MDCK II cells stably expressing GPC. GPC/GP2 were stained with a rabbit anti-LASV GP2 antibody (α4) and an anti-rabbit antibody conjugated with Alexa Fluor 680 and visualized using the Odyssey Infrared Imaging System (LI-COR Biosciences). As a control untransfected cells were used (Control; lane 1), as well as cell lysates stably expressing GPC (GPC; lane 2). **(B)** Transmission electron microscopy was performed with purified GPC VLPs after fixation with 1% PFA for 1 h. VLP suspension was applied to formvar-coated nickel grids and incubated for 10 min. After brief washing in water, the samples were negatively stained with 1% phosphotungstic acid. Micrographs were acquired at 120 kV using a JEOL JEM1400 equipped with a TVIPS TemCam F416 camera. Scale bar indicates 50 nm. **(C)** 2D class averages of nsEMPEM experiments with pAbs from rabbit 350 using GPCysRRLL-I53-50A pre-complexed with 18.5C. Classes shown are the selected classes from two iterations of 2D classification that have been reclassified to 16 classes. Two classes are pseudo-colored according to the legend below to indicate the stabilizing Fab (18.5C Fab), pFab and I53-50A. **(D)** Epitope footprints of GPC-C-1 and NAb 8.9F.[1] To determine the footprint of 8.9F, all GPC residues within a radius of 4 Å from 8.9F were highlighted. For the footprint of GPC-C-1, all GPC residues within a radius of 6 Å from GPC-C-1 were highlighted to compensate for the absence of modeled sidechains past Cβ. **(E)** Close up of map and model highlighting presumed contacts between the CDRH3 of GPC-C-1 and GPC. The highest probability residue as determined by ModelAngelo was modeled in the poly-alanine backbone and indicated with a label. **(F)** Same as (E) but now for GP1-A-1. **(G)** Epitope footprints of GP1-A-1 and NAb 12.1F.[1] To determine the footprint of 12.1F, all GPC residues within a radius of 4 Å from 12.1F were highlighted. For the footprint of GP1-A-1, all GPC residues within a radius of 6 Å from GP1-A-1 were highlighted to compensate for the absence of modeled sidechains past Cβ.

Table S1. 8.9F antibody interactions with GP1, related to Figure 1. Amino acid interactions at the 8.9F epitope-paratope region were determined using the online-based Epitope-Analyzer platform[8]. Since Epitope-Analyzer does not consider heteroatoms, interactions between sulfated tyrosines (TYS) and GPC were inferred from the structure using an overall distance cutoff of <4.0 Å and a distance cutoff of <3.5 Å for hydrogen bond interactions in particular.

Residue # (GPC)	Chain	Amino acid	Atom	Residue # (8.9F)	Chain	Amino acid	Distance (Å)	Predicted interaction
117	A	PHE	CE1-OH	100E	H	TYS	3.7	Van-der-Waals
119	A	ASN	OD1-CE2	100E	H	TYS	3.6	Van-der-Waals
121	A	SER	CB-CB	100D	H	TYS	3.7	Van-der-Waals
125	A	LYS	NZ-OD1	100A	H	ASP	2.4	Salt-Bridge
125	A	LYS	NZ-OD2	100A	H	ASP	3.3	Salt-Bridge
150	A	TYR	CE2-O	100H	H	ASP	3.9	Van-der-Waals
150	A	TYR	CE1-CG2	100I	H	VAL	3.9	Hydr-Phbc
256	A	ARG	NH1-OE1	100L	H	GLN	3.1	Hydro-Bond
256	A	ARG	NH1-CE	100G	H	MET	3.2	Van-der-Waals
150	B	TYR	OH-OD2	100O	H	ASP	3.4	Hydro-Bond
256	B	ARG	NE-OH	100C	H	TYS	3.5	Hydro-Bond
256	B	ARG	NH2-O1	100C	H	TYS	3.1	Salt-Bridge
256	B	ARG	NE-O1	100C	H	TYS	3.6	Salt-Bridge
256	B	ARG	CD-CE2	100D	H	TYS	3.9	Van-der-Waals
257	B	ARG	N-O1	100D	H	TYS	2.6	Hydro-Bond
257	B	ARG	NE-O1	100D	H	TYS	3.8	Salt-Bridge
257	B	ARG	NE-O2	100D	H	TYS	3.0	Salt-Bridge
257	B	ARG	NH2-O2	100D	H	TYS	2.9	Salt-Bridge
258	B	LEU	CB-CE2	100D	H	TYS	3.9	Hydr-Phbc
258	B	LEU	CB-CZ	100D	H	TYS	3.9	Hydr-Phbc
258	B	LEU	N-OH	100D	H	TYS	3.5	Hydro-Bond
150	C	TYR	OH-OD1	100A	H	ASP	2.4	Hydro-Bond
150	C	TYR	CE1-CB	100	H	ALA	3.5	Hydr-Phbc
150	C	TYR	CZ-CB	100	H	ALA	3.9	Hydr-Phbc
256	C	ARG	CG-OD1	100H	H	ASP	3.2	Van-der-Waals
256	C	ARG	CD-CB	100G	H	MET	3.3	Van-der-Waals
257	C	ARG	N-OD2	100H	H	ASP	2.6	Hydro-Bond
114	C	ASN	O-CA	30A	L	GLY	3.23	Van-der-Waals
114	C	ASN	ND2-CB	30	L	ILE	3.44	Van-der-Waals
115	C	HIS	CB-OD1	31	L	ASN	3.72	Van-der-Waals
116	C	LYS	N-CB	93	L	SER	3.95	Van-der-Waals
117	C	PHE	CZ-CE2	91	L	TYR	3.43	Hydr-Phbc
117	C	PHE	CZ-CD2	91	L	TYR	3.46	Hydr-Phbc
117	C	PHE	CE1-CE2	91	L	TYR	3.58	Hydr-Phbc
117	C	PHE	CE2-ND2	31	L	ASN	3.67	Van-der-Waals
117	C	PHE	CE2-CB	93	L	SER	3.72	Van-der-Waals
117	C	PHE	CZ-CE2	32	L	PHE	3.84	Hydr-Phbc
117	C	PHE	CE1-CE2	32	L	PHE	3.93	Hydr-Phbc
117	C	PHE	CE1-CD2	91	L	TYR	3.97	Hydr-Phbc

Table S2: Electron Microscopy Data Bank deposition information, related to Figures 2 and 3. Maps are accessible at emdataresource.org using the listed codes. Additional maps can be found on the "Download" tab of each entry.

	EMDB ID	Time point	Specificity	# particles (composite)	Map	Microscope	Pixel size
**Rabbit 187**	EMD-43174	30	Base	7,212	additional map	Tecnai Spirit	2.06
	EMD-43174	30	FP	10,038	additional map	Tecnai Spirit	2.06
	EMD-43174	30	GPC-A	16,689	main map	Tecnai Spirit	2.06
	EMD-43175	18	Base	8,882	additional map	Tecnai Spirit	2.06
	EMD-43175	18	FP	8,905	additional map	Tecnai Spirit	2.06
	EMD-43175	18	GPC-A	12,945	main map	Tecnai Spirit	2.06
**Rabbit 188**	EMD-43176	30	Base	9,484	main map	Tecnai Spirit	2.06
**Rabbit 189**	EMD-43177	30	Base	12,037	additional map	Tecnai Spirit	2.06
	EMD-43177	30	GPC-A	13,250	main map	Tecnai Spirit	2.06
	EMD-43178	18	Base	5,061	additional map	Tecnai Spirit	2.06
	EMD-43178	18	GPC-A	2,937	main map	Tecnai Spirit	2.06
**Rabbit 190**	EMD-43179	30	Base	6,805	additional map	Tecnai Spirit	2.06
	EMD-43179	30	FP	11,371	main map	Tecnai Spirit	2.06
**Rabbit 191**	EMD-43180	30	Base	11,393	main map	Tecnai Spirit	2.06
**Rabbit 192**	EMD-43181	30	Base	15,413	additional map	Tecnai Spirit	2.06
	EMD-43181	30	FP	15,136	main map	Tecnai Spirit	2.06
**LAVA05**	EMD-43182	30	Base	17,515	main map	Tecnai Spirit	2.06
**LAVA06**	EMD-43183	30	Base	19,440	main map	Tecnai Spirit	2.06

Table S3: Cryo-EM data collection and atomic model refinement, related to Figures 3, 4, 5, and 6. Model refinement and validation was performed in Phenix 1.21.[5]

	GPC bound to mAb 8.9F	GPC bound to pAb GPC-A-2 (r189)	GPC bound to pAb GPC-A-1 (r187)	GPC bound to pAb LL-1	GPC bound to pAb Base-1
**Access codes**					
PDB	9CJ7	9CK8	9CK7	9CJ8	8TYC
EMDB	EMD-45624	EMD-45644	EMD-45643	EMD-45625	EMD-41713
Genbank	NP_694870.1	NP_694870.1	NP_694870.1	NP_694870.1	NP_694870.1
**Data collection and processing**					
Microscope	Titan Krios	TFS Glacios II	TFS Glacios	TFS Glacios	Titan Krios
Magnification	130,000	190,000	190,000	190,000	105,000
Voltage (kV)	300	200	200	200	300
Electron exposure ($e^-/Å^2$)	50.0	41.21	43.68	43.68	50.35
Defocus range (μm)	-0.7 to -2.0	-0.8 to -2.0	-0.8 to -2.0	-0.8 to -2.0	-0.7 to -2.0
Camera	Gatan K2 Summit	TFS Falcon 4i	TFS Falcon 4	TFS Falcon 4	Gatan K3 Bioquantum
Pixel size (Å)	1.045	0.718	0.725	0.725	0.833
Imposed Symmetry	C1	C1	C1	C1	C1
Final particle number	73,222	86,915 (symmetry expanded)	53,020 (symmetry expanded)	22,783 (symmetry expanded)	61,338 (symmetry expanded)
Map resolution (Å)	3.0	3.0	3.5	3.7	3.3
FSC Threshold	0.143	0.143	0.143	0.143	0.143
Map sharpening B-factor ($Å^2$)	-35	-69.9	-79.8	-57.5	-60
**Model refinement and validation**					
Total Residues	1389	1284	1293	1258	1353
Amino-acids	1300	1225	1235	1210	1273
Carbohydrates	89	59	58	48	80
RMSD Bonds	0.01	0.01	0.01	0.01	0.01
RMSD Angles	0.95	0.84	0.91	1.03	1.00
**Ramachandran**					
Outliers (%)	0	0	0	0	0
Allowed (%)	4.18	4.53	4.83	4.69	4.48
Favored (%)	95.82	95.47	95.17	95.31	95.52
Rotamer outliers (%)	0	0	0.11	0	0
Clash score	2.9	3.5	4.7	4.0	2.8
Molprobity score	1.4	1.5	1.6	1.5	1.4
FSC model (0/0.143/0.5)	2.7/3.0/3.3	2.9/3.0/3.2	3.3/3.4/3.8	3.6/3.7/4.0	3.0/3.3/3.7
EMRinger score	4.17	4.82	3.38	2.04	2.83

	GPC bound to pAb Base-2	GPC bound to pAb FP-1	GPC monomer bound to pAbs Int-1 and Int-2	GPC bound to pAb GPC-C-1	GPC bound to pAb GP1-A-1
**Access codes**					
PDB	NA	8TYE	N/A	8VCV	8VE8
EMDB	EMD-45905	EMD-41715	EMD-41716	EMD-43141	EMD-43168
Genbank	NP_694870.1	NP_694870.1	NP_694870.1	NP_694870.1	NP_694870.1
**Data collection and processing**					
Microscope	Titan Krios	TFS Glacios	TFS Glacios	TFS Glacios II	TFS Glacios II
Magnification	105,000	190,000	190,000	190,000	190,000
Voltage (kV)	300	200	200	200	200
Electron exposure ($e^-/Å^2$)	50.35	43.3	49.5	40.7	40.7
Defocus range (μm)	-0.7 to -2.0	-0.8 to -2.0	-0.8 to -1.4	-0.8 to -2.0	-0.8 to -2.0
Camera	Gatan K3 Bioquantum	TFS Falcon 4	TFS Falcon 4	TFS Falcon 4i	TFS Falcon 4i
Pixel size (Å)	0.833	0.725	0.725	0.718	0.718
Imposed Symmetry	C1	C1	C1	C1	C1
Final particle number	14,690 (symmetry expanded)	59,236 (symmetry expanded)	20,839	89,195	146,722
Map resolution (Å)	3.8	3.8	8.1	2.8	2.8
FSC Threshold	0.143	0.143	0.143	0.143	0.143
Map sharpening B-factor ($Å^2$)	-80	-80	-896	-58	-64
**Model refinement and validation**					
Total Residues	N/A	1273	N/A	1345	1329
Amino-acids		1212		1270	1260
Carbohydrates		61		75	69
RMSD Bonds		0.01		0.01	0.01
RMSD Angles		0.98		0.85	0.88
**Ramachandran**					
Outliers (%)		0		0	0
Allowed (%)		4.92		2.56	3.39
Favored (%)		95.08		97.44	96.61
Rotamer outliers (%)		0		0	0
Clash score		5.9		2.9	2.3
Molprobity score		1.7		1.2	1.2
FSC model (0/0.143/0.5)		3.7/3.8/4.1		2.7/2.8/3.0	2.7/2.7/2.9
EMRinger score		2.53		6.06	6.08

References

1. Li, H., Buck, T., Zandonatti, M., Yin, J., Moon-Walker, A., Fang, J., Koval, A., Heinrich, M.L., Rowland, M.M., Diaz Avalos, R., et al. (2022). A cocktail of protective antibodies subverts the dense glycan shield of Lassa virus. Sci. Transl. Med. *14*, eabq0991.
2. Monigatti, F., Gasteiger, E., Bairoch, A., Jung, E. (2002). The Sulfinator: predicting tyrosine sulfation sites in protein sequences. Bioinformatics *18*, 769-770
3. Punjani, A., Rubinstein, J.L., Fleet, D.J., and Brubaker, M.A. (2017). cryoSPARC: algorithms for rapid unsupervised cryo-EM structure determination. Nat. Methods *14*, 290–296.
4. Pettersen, E.F., Goddard, T.D., Huang, C.C., Meng, E.C., Couch, G.S., Croll, T.I., Morris, J.H., and Ferrin, T.E. (2021). UCSF ChimeraX: Structure visualization for researchers, educators, and developers. Protein Sci. *30*, 70–82.
5. Liebschner, D., Afonine, P.V., Baker, M.L., Bunkóczi, G., Chen, V.B., Croll, T.I., Hintze, B., Hung, L.W., Jain, S., McCoy, A.J., et al. (2019). Macromolecular structure determination using X-rays, neutrons and electrons: recent developments in Phenix. Acta Crystallogr D Struct Biol *75*, 861–877.
6. Katz, M., Weinstein, J., Eilon-Ashkenazy, M., Gehring, K., Cohen-Dvashi, H., Elad, N., Fleishman, S.J., and Diskin, R. (2022). Structure and receptor recognition by the Lassa virus spike complex. Nature *603*, 174–179.
7. Enriquez, A.S., Buck, T.K., Li, H., Norris, M.J., Moon-Walker, A., Zandonatti, M.A., Harkins, S.S., Robinson, J.E., Branco, L.M., Garry, R.F., et al. (2022). Delineating the mechanism of anti-Lassa virus GPC-A neutralizing antibodies. Cell Rep. *39*, 110841.
8. Montiel-Garcia, D., Rojas-Labra, O., Santoyo-Rivera, N., and Reddy, V.S. (2022). Epitope-Analyzer: A structure-based webtool to analyze broadly neutralizing epitopes. J. Struct. Biol. *214*, 107839.
